# Toward Understanding CB[7]-Based Supramolecular Diels-Alder Catalysis

**DOI:** 10.3389/fchem.2020.587084

**Published:** 2020-11-06

**Authors:** Dénes Berta, István Szabó, Oren A. Scherman, Edina Rosta

**Affiliations:** ^1^Department of Physics and Astronomy, University College London, London, United Kingdom; ^2^Department of Chemistry, King's College London, London, United Kingdom; ^3^Melville Laboratory for Polymer Synthesis, Department of Chemistry, University of Cambridge, Cambridge, United Kingdom

**Keywords:** QM/MM, Diels-Alder (DA) chemistry, catalysis, confinement & solvent effect, quantum chemistry *ab initio*

## Abstract

Cucurbiturils (CBs) are robust and versatile macrocyclic compounds, often used as molecular hosts in complex supramolecular systems. In previous work, remarkable catalytic activity has been observed for asymmetric cycloadditions under very mild conditions. Herein, we investigate the nature of supramolecular catalysis using DFT calculations and QM/MM techniques. We discuss induced conformational changes, electrostatic shielding effects from the highly polar aqueous environment and cooperativity in hydrogen bonding of the substrates in explicit water using QM/MM simulation techniques. Our results show little specificity for the chosen molecules, suggesting an excellent opportunity to expand the scope for catalytic use of these supramolecular macrocyclic containers.

## Introduction

Sustainable catalysis is evolving to limit the environmental impact of chemical industries. Organocatalytic and supramolecular systems are successfully developed offering mild reaction conditions and great selectivity. These are often aimed at mimicking enzymes that achieve the highest catalytic efficiencies as well as specificity. Cucurbit[*n*]urils (CB[*n*], *n* = 5–8), are great supramolecular hosts, creating a microscopic environment while maintaining good solubility in water (Barrow et al., 2015). Besides participating in host-guest chemistry (Zhang et al., 2012; Wu et al., 2017, 2019; Olesińska et al., 2019; Huang et al., 2020), they were successfully utilized in catalytic applications (Wang et al., 2011; Taylor et al., 2013; Zheng et al., 2015; Palma et al., 2017; Ren et al., 2018; Rad et al., 2019), surface chemistry (Wagner et al., 2020), and sensing and detection (Huang et al., 2012; Kasera et al., 2012; Biedermann et al., 2015; de Nijs et al., 2017, 2019).

Substrate binding within CB[*n*]-cavities is usually observed spectroscopically (through a variety of NMR techniques, surface Raman spectroscopy as well as UV/Vis and PL spectroscopies) (del Barrio et al., 2016; Sigle et al., 2016) and using isothermal titration calorimetry (ITC) experiments. However, thermodynamic parameters and binding constants attainable by these experiments do not alone explain the catalytic effect and more structural insights are needed to further explore these phenomena. Computational studies can account for many aspects of the catalysis (Harvey et al., 2019) enhancing our understanding of the origins of these effects. Cucurbiturils are subject to many computational works, often in conjecture with experiments, contributing to interpretation of spectroscopy (Chio et al., 2019), electronic properties (Sin et al., 2020) and in particular binding free energies and structures (Yin et al., 2016; Senthilnathan et al., 2018; Olesińska et al., 2019; Wu et al., 2019). As hosts, CBs were featured in many blind prediction challenges (Muddana et al., 2012, 2014; Lee et al., 2017; Rizzi et al., 2018).

**Figure 1:**
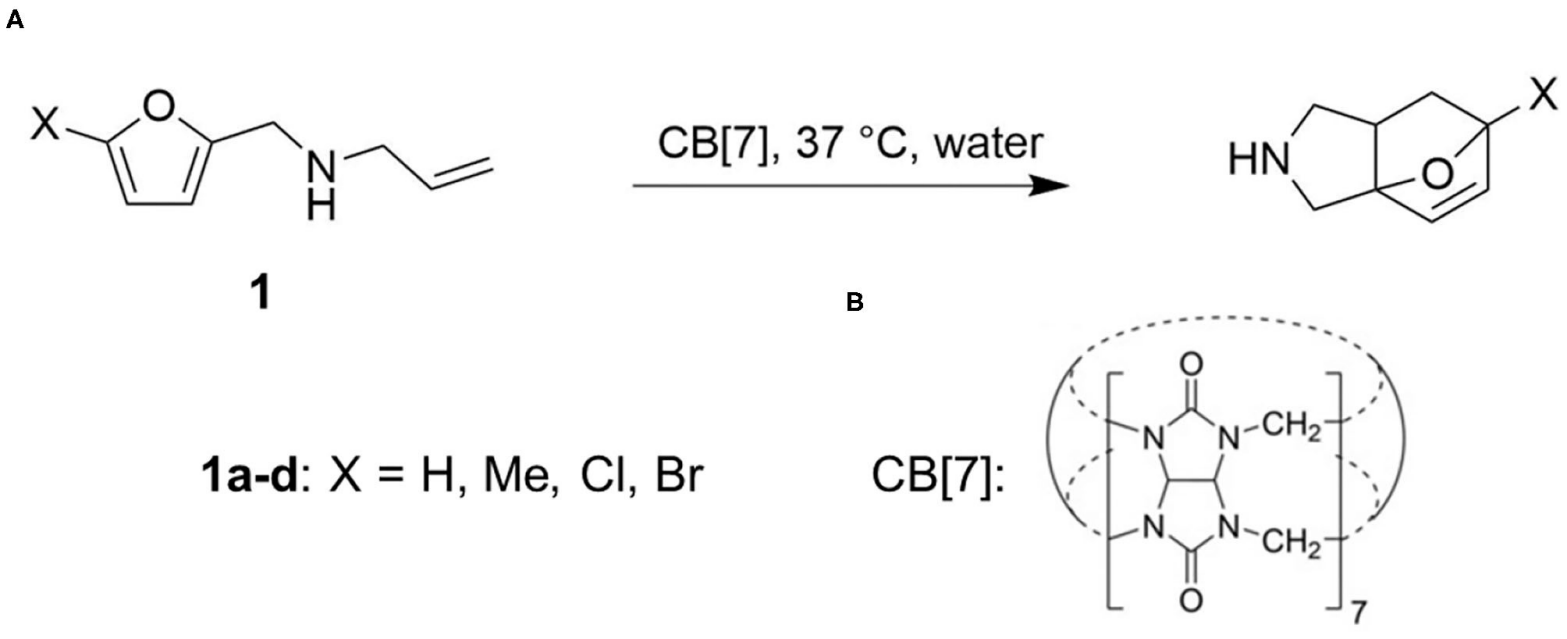
**(A)** CB[7] catalyzed [4+2] cycloaddition of *N*-allyl-2-furfurylamine derivatives. **(B)** Structure of cucurbit[7]uril (CB[7]).

Herein we focus on the CB-modulated catalysis of intramolecular Diels-Alder cycloaddition reactions (Palma et al., 2017) (Figure 1). *N*-allyl-2-furfurylamines are known model substrates for Diels-Alder reactions (Andrés et al., 1997; Gschwend et al., 2002). Based on the cavity size and our previously reported binding studies, CB[7] was found to be the best host homolog to offer enhanced water solubility allowing for higher concentrations, conformational restriction and tight binding. In physiological conditions, low conversion is achieved, while in the presence of CB[7], the cycloaddition rates are significantly sped up, and the reaction goes to completion within minutes. Importantly, at 10 mol% catalyst loading, the reaction still occurred at an increased rate, indicating a catalytic turnover in the order of organocatalysts. This work was the first example of carrying out this reaction in aqueous environment without incorporation of protecting groups and was compared to the activity of natural Diels-Alderase (Ose et al., 2003; Byrne et al., 2016).

We have previously found that binding in CB favors the closed conformation of the reactants, in agreement with experimental NMR measurements. The confinement of the reacting molecules have also been taken into account by computations (Chakraborty and Chattaraj, 2018). However, these binding effects amounting to 1–2 kcal/mol, only partially explain the catalytic effects of 4–5 kcal/mol, which have so far been unresolved. We now investigate the reaction mechanism determining detailed underlying electronic structures to assess catalysis. Using continuum electrostatic model to account for the water environment, we obtained a good agreement between experimental and calculated reaction barriers without CB. However, in CB, the catalytic rate enhancement is not reproduced without taking into account explicit water molecules. Our QM/MM calculations reconcile these differences, and we are able to assess the catalytic effects upon binding in CB. Our results show that the catalytic effects are arising as a combination of confinement, electrostatic shielding and shifting the pK_a_ of the ligand. In view of future design, we also demonstrate that DFT calculations can provide detailed electrostatic maps that elicit an optimal environment for efficient catalysis.

## Materials and Methods

Classical MD simulation were carried out using CHARMM36 force field (Best et al., 2012) as implemented in NAMD 2.12 (Phillips et al., 2005). A box of 3855 TIP3 waters solvating the complex was minimized in 20,000 steps, followed by 0.1 ns of NVT equilibration constraining the heavy atoms of **1a** and CB[7]. Independent replicas of 1 ns unbiased MD were run with 2 fs timestep in NTP ensemble, coupled to a Langevin thermostat at a temperature of 303.15 K and a Langevin barostat at standard pressure. Non-bonded interactions were modeled with scaled 1–4 setting in NAMD and a cutoff of 12 Å with switching distance of 10 Å. The Particle Mesh Ewald method was used to evaluate long range electrostatic interactions. Subsequently, conformations for DFT calculation were chosen by conformation clustering done by a standalone utility shipped with Schrödinger Suite.

Intermediates and transition states have been optimized with B3LYP hybrid functional (Becke, 1993) and 6-31G^*^ Pople basis set (Ditchfield et al., 1971; Hehre et al., 1972) using Grimme's D3 dispersion correction (Grimme, 2012) as implemented in Gaussian 09 Revision E (Frisch et al., 2009). Vibrational analysis and PCM solvent correction with Thrular's SMD parametrization was also carried out at the aforementioned level of theory (Marenich et al., 2009). For the sake of accuracy, final single point energies were calculated with 6-311++G(3df,3pd) basis set. Numerical integrals were computed on ultrafine grid. The nature of transition states was verified by Intrinsic Reaction Coordinate (IRC) calculations.

QM/MM calculations were done to study explicit solvation using TIP3P solvation model. The CB[7] and **1a** was parametrized by CGenFF (Vanommeslaeghe and MacKerell, 2012), the guest was treated at B3LYP/6-31+G^*^ level of theory. Instead of periodic boundary conditions, the system was trimmed to a sphere of 25 Å from the substrate, resulting in 2,907 or 2,866 explicit water molecules without or with CB[7], respectively. Atoms further than 20 Å away from the QM region were kept frozen throughout the simulations. Minimizations were run by CHARMM (Brooks et al., 2009) interfacing with Q-Chem 4.3 (Shao et al., 2015). The reaction path was sampled at discrete steps 0.2 Å apart along the reaction coordinate defined by the sum of the distances of the forming two sigma bonds. Quadratic constrains were defined with a force constant of 1,000 kcal mol^−1^ Å^−2^, path scans were run until the convergence of the potential energy surface, then energies were recalculated using the 6-311++G(3df,3pd) basis set.

The cubic grid for the point charge analysis was made with a 0.5 Å spacing around the aligned reactant and transition state of the substrate **1a**, to a maximum distance of 3 Å from the molecule. Points closer than 1.5 Å to any atom were removed. Barriers were recalculated with B3LYP/6-31+G^*^ using Q-Chem 4.3.

## Results

We considered the cycloaddition reaction in four substrates (**1a–d**) obtained from substitution from *N*-allyl-2-furfurylamine molecule (Figure 1). To assess the catalytic effects, we compared reaction rates between water and in the presence of one equivalent of CB[7] (Figure 1B), which presents a 1:1 binary complex. To obtain the barriers, we identified reactant, product and transition states (RS, PS, and TS). We did not investigate the binding affinity of the substrates in CB[7], since the complexation is experimentally confirmed. As experimental reference results for the catalytic effects, we used barriers derived based on first order kinetics presented in reference (Palma et al., 2017). The introduction of CB[7] induces a ca. 5 kcal/mol drop in the activation free energy (Table 1).

**Table 1:** Summary of experimental kinetic data from Palma et al. (2017), barriers are calculated based on first order kinetics to allow direct comparison with calculations.

**X**	**H**	**Me**	**Br**	**Cl**
Time to half conversion/h	-	-	240	288
Barrier/kcal mol^−1^	28.7	28.6	26.8	26.7
Time to half conversion/h with 1 eq CB[7]/h	1.2	0.2	0.3	0.5
Barrier/kcal mol^−1^ with 1 eq CB[7]	23.6	22.5	22.7	23.0

### Protonation of the Substrates

First, we considered the effect of protonation on the amine moiety on the rate of cycloaddition. Since positively charged compounds are generally the most potent to bind into CB structures, due to the favorable interaction with the carbonyl groups, the protonation equilibrium is likely to be upshifted in the presence of CB[7].

In experiments, pH was ~7.4, while the pK_a_ of the amine is estimated ~10 based on the DFT free energies and empirical prediction (details in Supplementary Material). Henceforth, we consider a protonated substrate for all calculations. Nevertheless, we compared the neutral **1a** compound in our preliminary calculations obtaining that it undergoes the cycloaddition with a barrier of 30.6 kcal/mol, while after protonation decreases this to 28.6 kcal/mol. This points to the beneficial effects due to the increased pK_a_ of the amine in the CB environment, but does not fully account for the catalytic effects observed.

### Reaction Without CB[7]

**Figure 2:**
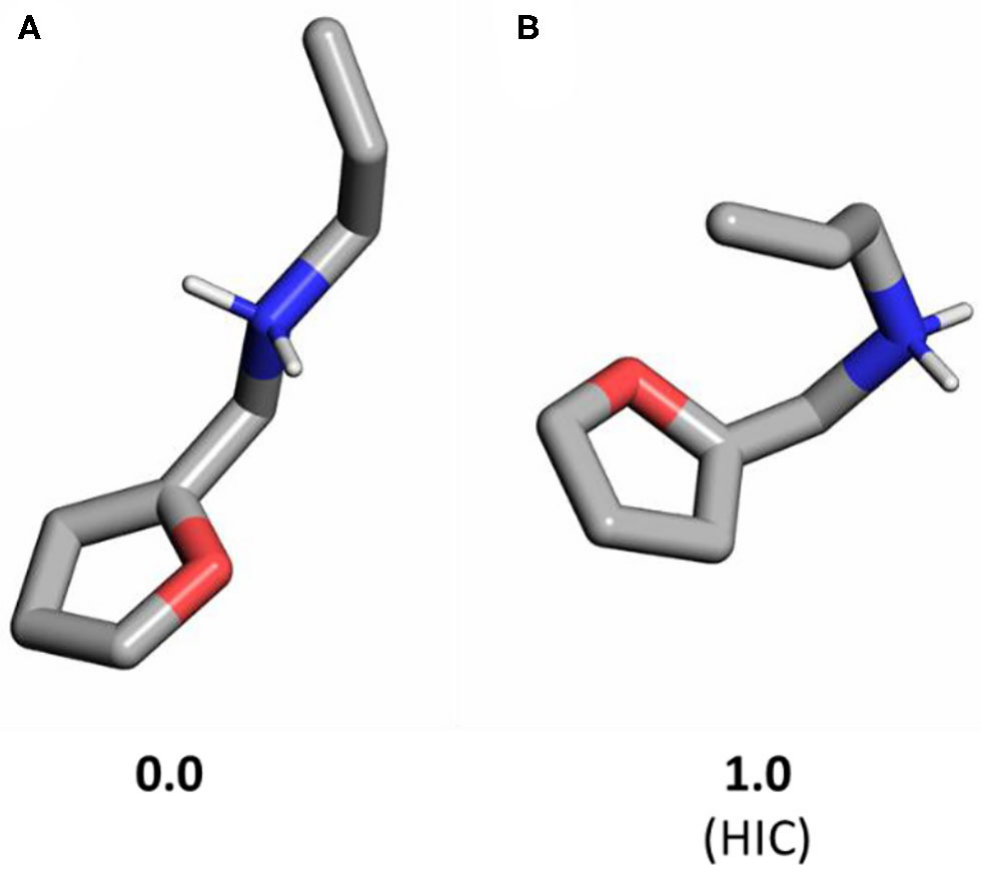
Conformers of substrate **1a** without CB[7]: **(A)** most stable unfolded structure; **(B)** hair-pin induced (HIC) conformer directly preceding the Diels-Alder transition state (yielded by an IRC calculation). Relative Gibbs free energies are shown in kcal/mol. Non-polar hydrogens are hidden for clarity.

We calculated the reactant and transition states for all four differently substituted substrates to obtain the reaction barriers in solution. Geometry optimizations were carried out in the gas phase for the TS and for several reactant (Figure 2) and product conformers, followed by a continuum dielectric model to account for free energies in the water environment.

Considering the binding poses within the CB[7] cavity, the substrate needs to adopt a folded structure, which is readily aligned for cycloaddition (HIC, hair-pin induced conformation) (Palma et al., 2017). Therefore, we calculated the most stable open and HIC conformers of the reactant state for **1a** that show a 1 kcal/mol free energy difference only. This demonstrates that additional effects beyond a HIC starting geometry also stabilize the transition state in the presence of CB.

Our calculated free energy barriers, summarized in Table 2, show a very good agreement with the experimental kinetic rates for all four compounds. The reaction barrier with the Me substituted **1b** substrate is somewhat higher in the calculations as compared with experiment, whereas the other three ligands match the barriers within 0.2 kcal/mol. We also compared the free energy barriers using a range of functionals that all performed similarly. Small improvements can be observed using Minnesota functionals when we compare the barrier heights for the H and Me substituted ligands, **1a** vs. **1b** (Supplementary Table 2).

**Table 2:** Comparison of experimental (as shown in Table 1) and calculated barriers calculated by DFT.

**X**	**H**	**Me**	**Br**	**Cl**
Exp. barrier/kcal mol^−1^	28.7	28.6	26.8	26.7
Comp. barrier/kcal mol^−1^	28.6	29.5	26.9	26.9
Exp. barrier/kcal mol^−1^ with 1 eq CB[7]	23.6	22.5	22.7	23.0
Comp. barrier/kcal mol^−1^ with 1 eq CB[7]	24.6	26.6	22.3	21.7

Without the CB[7], the model considered implicit solvent, while the catalytic system consisted of a single explicit water molecule in addition. Values are in kcal/mol, calculations were done at the B3LYP-D3/6-31G*//B3LYP-D3/6-311++G(3df,3pd) level.

### Reaction in the CB[7] Cavity

Initial MD simulations were used to obtain several conformers of host-guest complexes as starting points in DFT optimization for the stationary reactant, transition, and product states. Most interestingly, the presence of CB[7] leads to similar barriers as obtained in implicit water calculations without CB[7] (see Supplementary Material). To account for the missing catalytic effects, we therefore decided to incorporate explicit water molecules in the DFT calculations.

**Figure 3:**
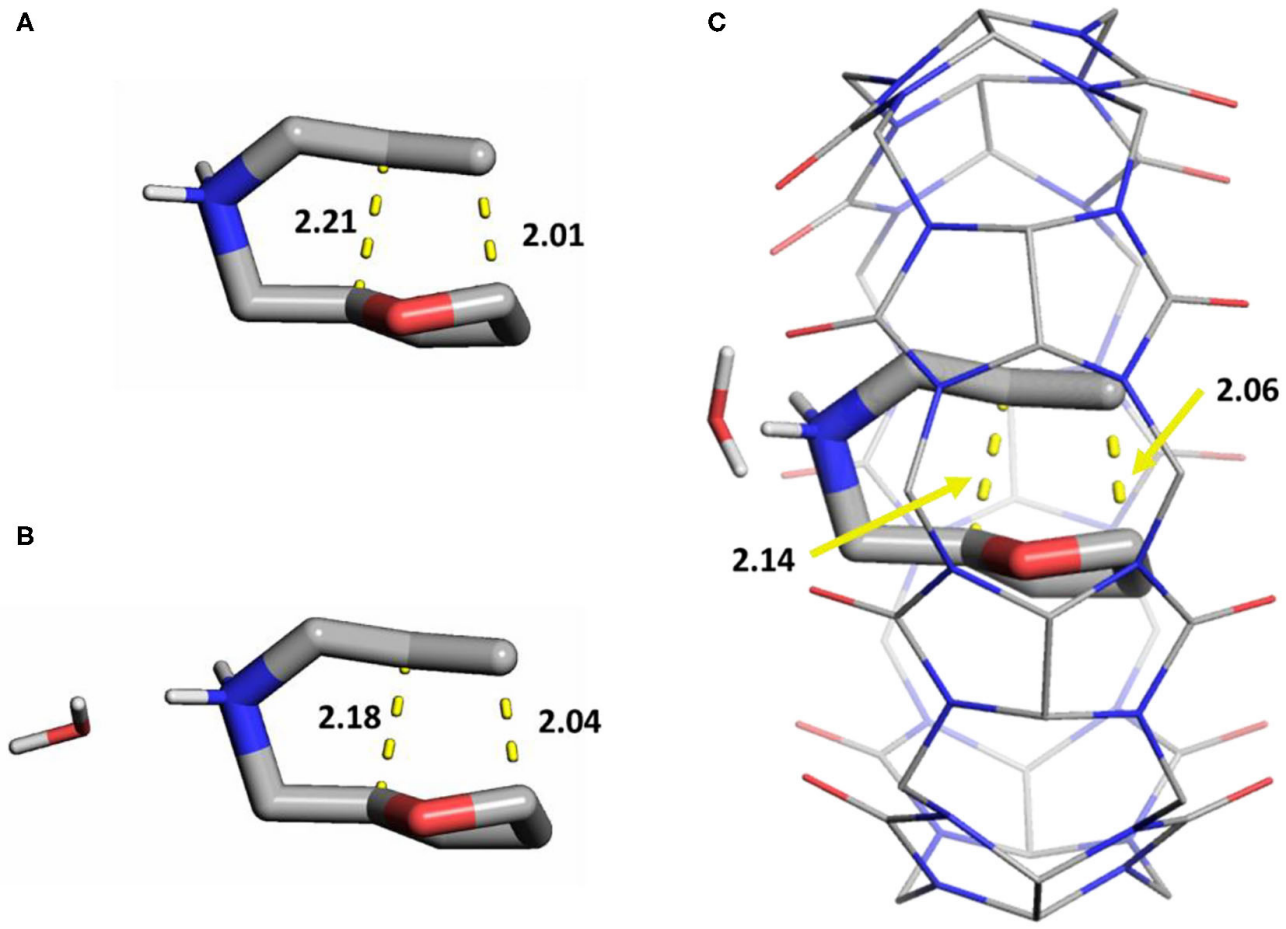
Transition state structures of the cycloaddition in different models for the reaction with **1a** (licorice): **(A)** only implicit water, **(B)** one explicit water molecule, **(C)** one explicit water and **1a** in complex with CB[7] (lines). Distances of the forming sigma bonds (yellow dashes) are shown in Å. Non-polar hydrogens are hidden for clarity.

We initially investigated the effects of a single water molecule for the catalytic barrier (Figures 3A,B). We placed the water molecule near the ammonium cation moiety given their strong interactions, and reoptimized the stationary structures. This resulted in small rearrangements in the binding mode of the substrate in the cavity, as the NH_2_ does not directly interact with the CB[7]'s carbonyl oxygens, but through the explicit water instead (Figure 3C). The free energy barrier of the reaction in this model is 24.6 kcal/mol, which is in satisfactory agreement with the experimental results.

In comparison, analogous geometry optimizations for the non-catalytic system without CB[7], in the presence of a single water molecule does not change either the stationary structures or the barriers significantly (Supplementary Tables 1, 2). This suggests a cooperative influence of the solvent and the CB[7], which can only be incorporated with explicit solvation. Therefore, the stationary points along the reaction were calculated for all four substrates in the presence of one water molecule besides the macrocycle. The results are summarized in Table 2 showing a good agreement between this model and the experiments. The level of theory for this series was benchmarked to cover other functionals and Pople basis sets, maintaining the same trends in all cases (Supplementary Tables 1, 2).

### QM/MM Calculations in Explicit Water

To study the reaction featuring multiple water molecules, the most stable conformers of **1a** were solvated in explicit water and subjected to reaction path scans using QM/MM calculations. The activation energy was 25.9 kcal/mol in solution, and 20.8 kcal/mol in complex with CB[7], minimized at the level B3LYP/6-31+G^*^ and the energies recalculated with the basis set 6-311++G(3df,3pd).

**Figure 4:**
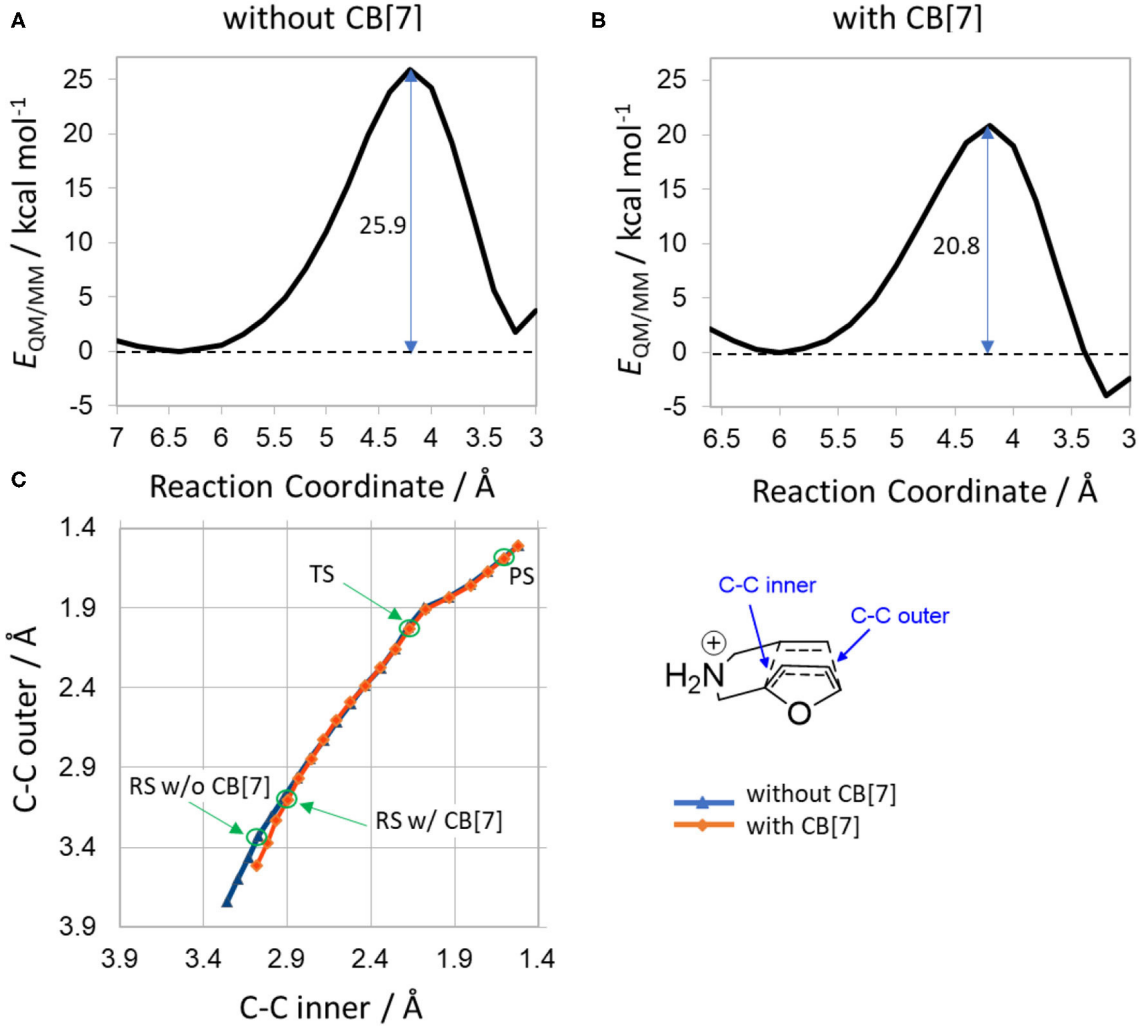
Results of QM/MM potential energy scans **(A)** without CB[7] and **(B)** with CB[7]. The reaction coordinate is the sum of the distances of C-C inner and C-C outer bonds. Reaction barriers are shown in kcal/mol, indicated by vertical arrows in the profiles. **(C)** Change in biased distances along the scan (red/blue symbol with/without CB[7]). Bond definitions are highlighted in the inset. Note that these bonds are not individually constrained, but their sum.

The energy profiles depicted in Figure 4 exhibit a ca. 5 kcal/mol decrease in activation energy when the substrate is bound to CB[7], accounting for the overall catalytic effect fully. The only main difference in the reaction paths is observed for the reactant state regions. The reactant minima are located at reaction coordinate values 6.4 Å in water and 6.0 Å with CB[7]. Therefore, the RS conformations are more contracted inside the cavity before the cycloaddition, underlining the importance of pre-selecting conformers which easily undergo the cyclization. The overall barriers are somewhat lower than the ones presented in Table 2, which can be attributed to the different approach of solvent modeling.

Similarly, we also performed QM/MM calculations on the **1b** substrate with a methyl substituent, as well as a nitro group (**1e**, Supplementary Figure 4) to analyze the trends in the barrier heights and the catalytic effects (Supplementary Figures 3, 5, respectively). We obtained a barrier height of 26.5 kcal/mol in water and 20.5 kcal/mol in CB[7] for 1b, showing a somewhat slower reaction in water, but faster in CB[7]. The QM/MM calculations thus improved the agreement with the experimentally observed catalytic effect for **1b**, as also seen for compound **1a**.

**Figure 5:**
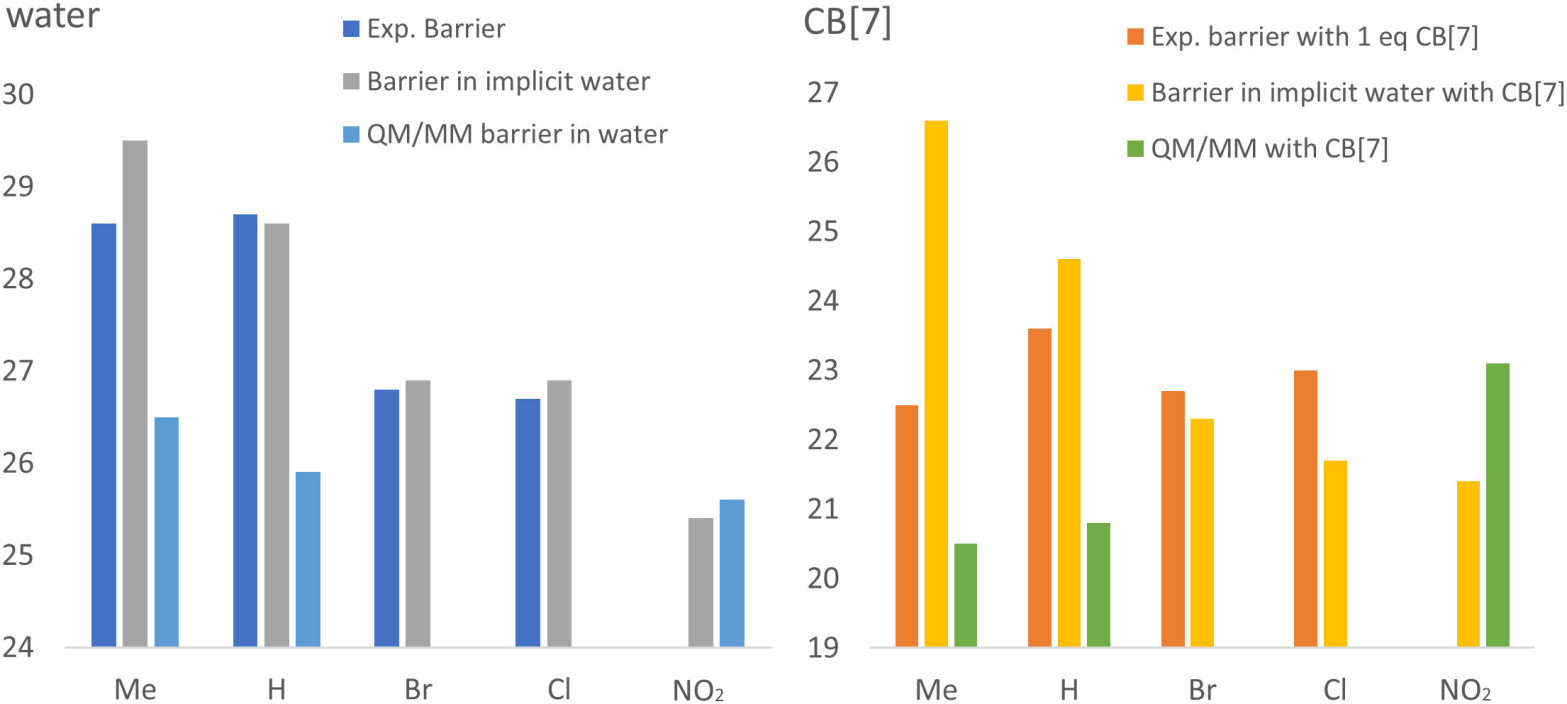
Experimental and calculated trends for reaction barriers obtained in water (left) and catalyzed by CB[7] (right). Compounds **1b**, **1a**, **1c**, **1d**, and **1e** are shown ordered in increasing electron withdrawing ability. The experimental (dark blue in water and red in CB[7]) and calculated (gray and light blue in water and orange and green in CB[7], for implicit water and QM/MM, respectively) energy barriers are shown in kcal/mol.

Overall, a clear trend is observed both in experiments and in calculations in water, without CB[7]. More electron withdrawing groups reduce the barrier more, therefore we predict the fastest reaction in water with the nitro group. Figure 5 interestingly, this trend is absent from the reactions with CB[7]. In this case, experimental barriers are comparable, no clear trend is observed. This is not observed in implicit solvent calculations with CB[7], and the prediction for the methyl substituent is affected the most. Therefore, the lack of explicit solvent in this case probably cannot accurately account for the ligand's geometry and bound position within the CB[7], which leads to the less accurate estimate of the catalytic effects. On the other hand, when using QM/MM, the presence of the water molecules allows us to obtain a significantly closer agreement with experiment for the catalytic effects. In this case, although the barriers both in water and in CB[7]-catalyzed environment are slightly underestimated, the overall catalytic effect is more accurately reproduced.

### Point Charge Analysis

**Figure 6:**
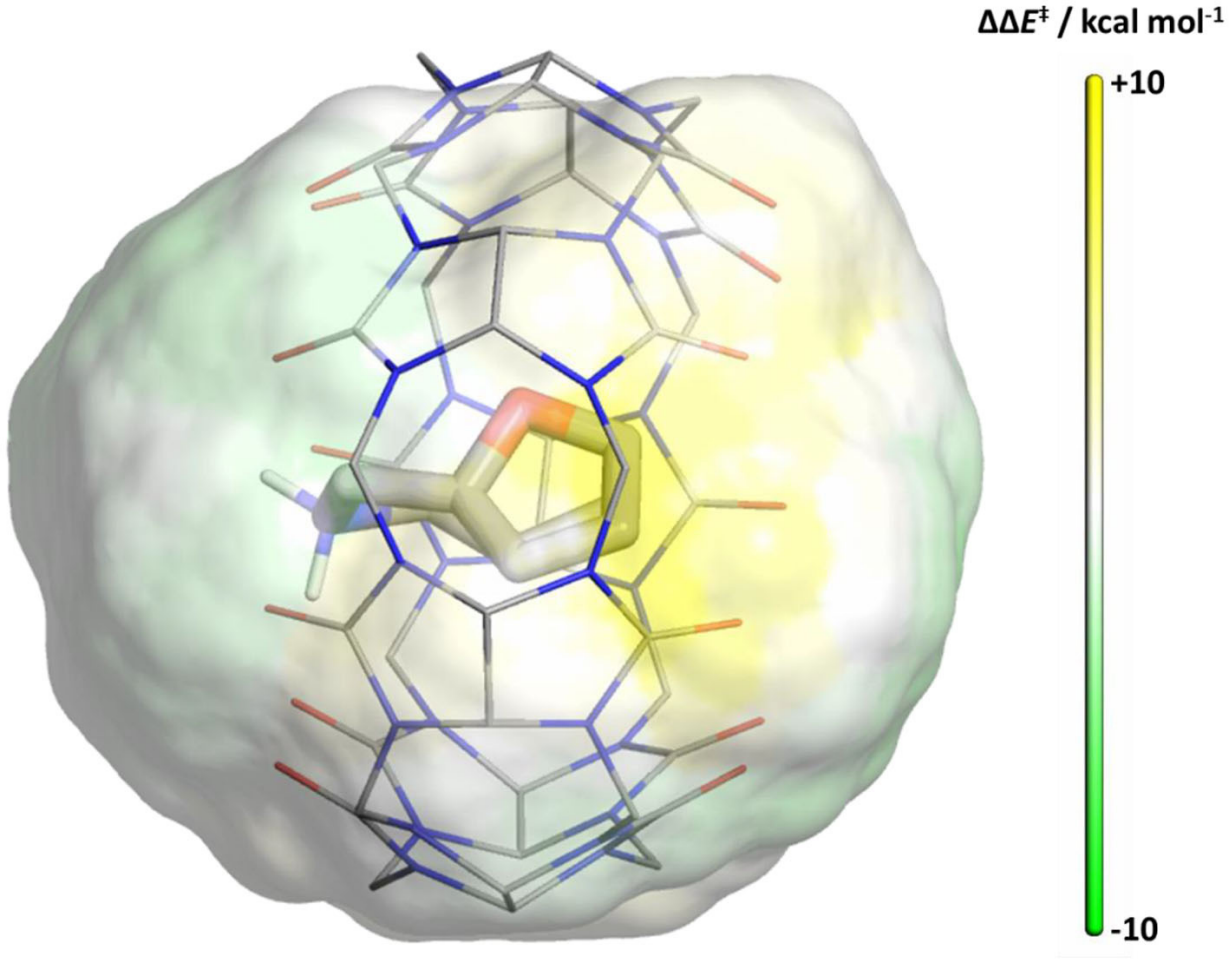
Map of reaction barrier changes upon the addition of a probe −1 point charge (calculated at B3LYP/6-31+G*). Yellow regions indicate an increased barrier height for the reaction, whereas green regions favor nucleophilic groups at these locations for reducing the barrier heights as shown in color scale (capped at ±10 kcal/mol). CB[7] is overlaid with the results to highlight the area shielded from the polar environment.

The well-defined Diels-Alder transition states enable us to study the shielding effect of CB[7] in an environment where molecular details are removed, and only electrostatic interactions are considered more generally. We placed probe point charges around the continuum solvent optimized reactant and transition states on a grid around the molecule and recalculated the energy barriers. We defined a cubic 3D grid up to 3.0 Å around the substrate with a 0.5 Å spacing to evaluate the electrostatic influence on the reaction barrier. Placing a −1 probe charge (given the cationic substrate) around the reacting molecule outlines the pattern on the catalytic effect depicted in Figure 6. The surface was obtained by selecting the grid points that are located at within 2.0–2.3 Å distance from the ligand, where solvent molecules would surround the substrate. Yellow regions indicate an increased barrier height for the reaction when negatively charged groups, such as the oxygen of water molecules would be present, whereas green regions favor electrophilic groups at these locations for reducing the barrier heights. Our results using a +1 probe charge on the grid points results in analogous complementary effects (Supplementary Figure 6).

## Discussion

DFT calculations using continuum solvent models reproduce catalytic barriers to excellent accuracy as compared with experimental values. The results are consistent between tested functionals and basis sets within the acceptable error margin of DFT. However, the reaction barriers in binary complex with CB[7] are not able to capture the catalytic effects observed experimentally. Therefore, we used explicit water molecules as well as QM/MM calculations that successfully resolve the differences. Nevertheless, the absolute barriers are underestimated by the QM/MM minimizations, which can possibly be resolved by free energy calculation methods such as finite temperature string simulations.

Our calculations suggest that the reaction path is slightly modified in CB[7] as there the compressed reactant state conformation is stabilized upon complexation. Explicit water molecules in this case enable us to obtain the correct binding orientation within CB[7], which lead to the decreased reaction barriers. Furthermore, electrostatic effects are also beneficial that shield the substrate from interactions with water.

**Figure 7:**
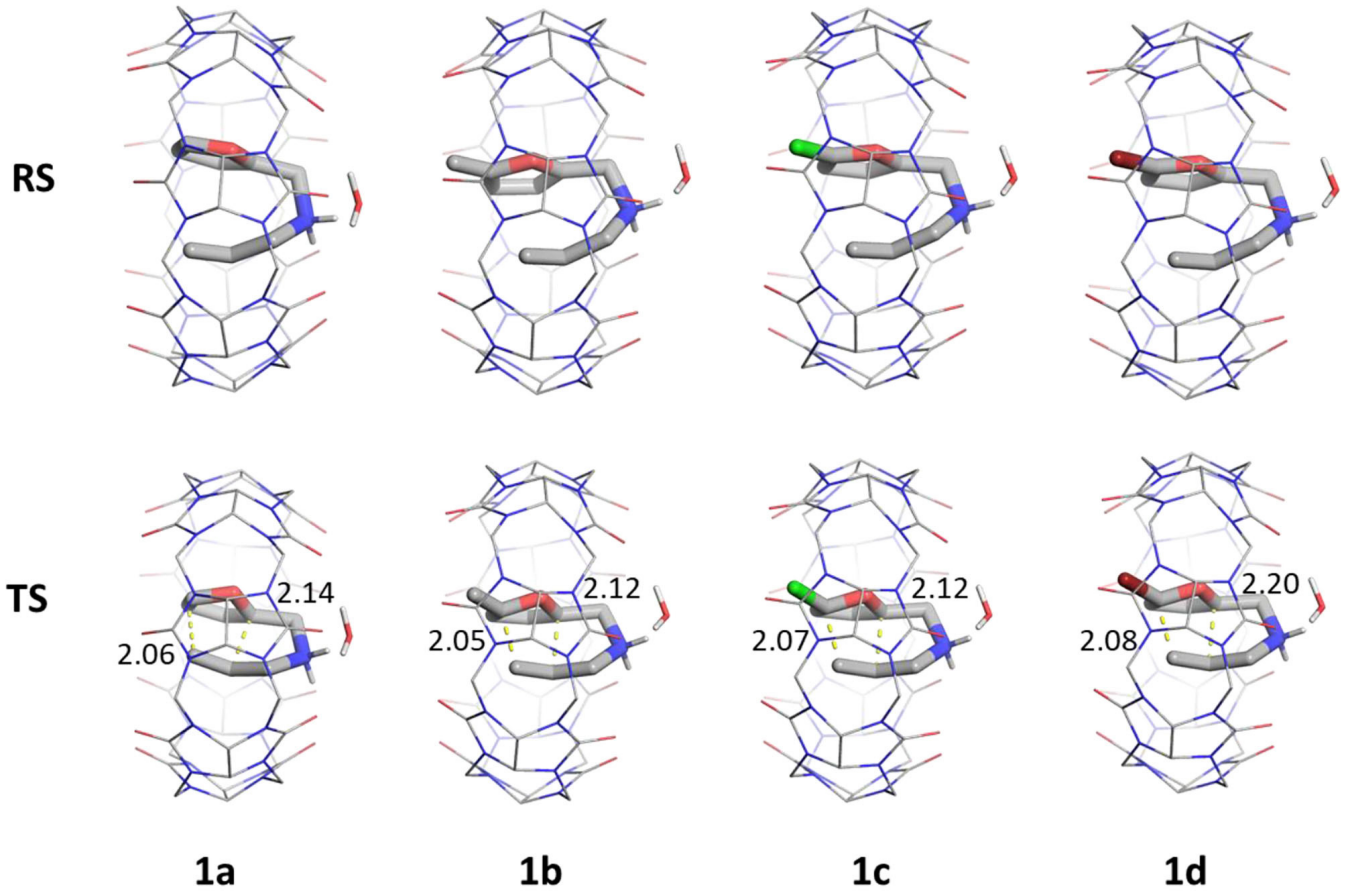
Reactant complexes (RS, top) and transition states (TS, bottom) of ligands **1a–d** in the presence of CB[7] (lines) and a single explicit water. Distances of C-C outer (left) and C-C inner (right) are shown in Å. Non-polar hydrogens are hidden for clarity.

Our results in conjunction with the experimental rates suggest that the four substituents have a similar catalytic effect (Figure 7). This is also seen from the similar geometric distances: C-C inner and C-C outer bonds observed in the stationary states determining the catalytic barrier heights. Therefore, these results can be generally applied to a range of molecules with analogous intramolecular Diels-Alder reaction mechanism that also contain an amine group, facilitating the binding and directing the orientation of the substrate. Extensions might include intermolecular reactions (Jon et al., 2001; Ren et al., 2018), where a larger CB[8] group might be necessary to accommodate both reacting partners.

The increasing electron withdrawing effects of the substrate substituents lead to faster reaction rates in water, as observed both in calculations and in experiment, including our predicted nitro substituent (Figure 5). Interestingly, the catalytic effects also depend on geometric factors of these substituents, as the methyl and nitro groups deviate from the trend observed for the H, Br, and Cl series. The methyl group has a slightly enhanced catalytic effect, whereas the nitro group is relatively slower in CB[7].

To more generally assess electrostatic factors governing the reaction, we probed the catalytic effects of single point charges placed on a 3D grid around the substrate. We found that the regions, where the nearby negative charges unfavorably affect the reaction, largely overlap with the area that the CB[7] shields from solvent upon complexation. These results are consistent upon using positive probe charges that show complementary effects. We envision that these maps quantifying electrostatic effects for the reaction barrier will also enable future design for improving catalysis.

## Conclusions

One of the first catalytic reactions where CBs were successfully used to enhance the rates of are the examples of Diels-Alder reactions. Our computational study elaborates the catalytic effect of cucurbit[7]uril on the cycloaddition reaction. The supramolecular host binds the substrate creating a favorably anisotropic environment preparing the cycloaddition both conformationally and electrostatically.

With DFT calculations and implicit solvation, we are able to reproduce the kinetics of the non-catalytic cycloaddition with great accuracy. However, the supramolecular catalytic effect is only interpretable by describing the environment more precisely, taking the solvent and their interaction with the substrate and the catalyst into account at the atomic level. Our results hence underline the limitations of implicit solvation and the cooperativity between the catalyst and the solvent.

In particular, a clear trend was observed in water for different substituents. The more electron withdrawing substituent used, the lower the barrier. This trend was not observed in the presence of CB[7] experimentally. In this case, the geometric effects due to interactions with solvent molecules likely play an additional important role in determining the reaction barriers, due to the ligand occupying the CB[7] cavity in slightly different positions. This is also supported by the fact that implicit solvent calculations continue to show a similar trend even in the presence of CB[7], whereas QM/MM calculations with explicit water, and computational models with added explicit water molecules more accurately reproduce the barrier heights for several substrates. Overall, both experimental and calculations suggest that the largest catalytic efficiency is achieved for the **1a** and **1b** substrates.

We recognize the natural direction of expanding the scope of this type of supramolecular catalysis based on the utilization the hydrophobic cavity and the H-bonding network formed by the solvent, polar groups and the portal of the CBs. Our general approach of mapping the influence of the electrostatics of the ligand's environment using simple 3D grid of point charges can aid this design of catalysts or additional substrates, where specific host-guest complex-based catalysis can expand to.

## Data Availability Statement

The raw data supporting the conclusions of this article will be made available by the authors, without undue reservation.

## Author Contributions

OS and ER conceptualized the research, IS did the MD simulations, DB carried out the QM and QM/MM simulations and processed the results. All authors contributed to the article and approved the submitted version.

## Conflict of Interest

The authors declare that the research was conducted in the absence of any commercial or financial relationships that could be construed as a potential conflict of interest.

## References

[B1] Andrés C. Maestro G. Nieto J. Pedrosa R. García-Granda S. Pérez-Carreño E. (1997). Diastereoselective intramolecular Diels-Alder reaction of the furan diene. A facile access to enantiopure epoxy tetrahydroisoindolines. Tetrahedron Lett. 38, 1463–1466. 10.1016/S0040-4039(97)00058-0

[B2] Barrow S. J. Kasera S. Rowland M. J. Del Barrio J. Scherman O. A. Jesús Del Barrio J.. (2015). Cucurbituril-based molecular recognition. Chem. Rev. 115, 12320–12406. 10.1021/acs.chemrev.5b00341 26566008

[B3] Becke A. D. (1993). Density-functional thermochemistry. III. The role of exact exchange. J. Chem. Phys. 98, 5648–5652. 10.1063/1.464913

[B4] Best R. B. Zhu X. Shim J. Lopes P. E. M. Mittal J. Feig M.. (2012). Optimization of the additive CHARMM All-Atom protein force field targeting improved sampling of the backbone φ, ψ and side-chain χ _1_ and χ _2_ dihedral angles. J. Chem. Theory Comput. 8, 3257–3273. 10.1021/ct300400x 23341755 PMC3549273

[B5] Biedermann F. Hathazi D. Nau W. M. (2015). Associative chemosensing by fluorescent macrocycle–dye complexes – a versatile enzyme assay platform beyond indicator displacement. Chem. Commun. 51, 4977–4980. 10.1039/C4CC10227D 25622263

[B6] Brooks B. R. Brooks C. L. Mackerell A. D. Nilsson L. Petrella R. J. Roux B.. (2009). CHARMM: the biomolecular simulation program. J. Comput. Chem. 30, 1545–1614. 10.1002/jcc.21287 19444816 PMC2810661

[B7] Byrne M. J. Lees N. R. Han L.-C. van der Kamp M. W. Mulholland A. J. Stach J. E. M.. (2016). The catalytic mechanism of a natural Diels–Alderase revealed in molecular detail. J. Am. Chem. Soc. 138, 6095–6098. 10.1021/jacs.6b00232 27140661

[B8] Chakraborty D. Chattaraj P. K. (2018). Confinement induced thermodynamic and kinetic facilitation of some Diels-Alder reactions inside a CB[7] cavitand. J. Comput. Chem. 39, 151–160. 10.1002/jcc.25094 29094421

[B9] Chio W.-I. K. Peveler W. J. Assaf K. I. Moorthy S.. (2019). Selective detection of nitroexplosives using molecular recognition within self-assembled plasmonic nanojunctions. J. Phys. Chem. C 123, 15769–15776. 10.1021/acs.jpcc.9b02363 31303905 PMC6614880

[B10] de Nijs B. Carnegie C. Szabó I. Grys D.-B. Chikkaraddy R. Kamp M.. (2019). Inhibiting analyte theft in surface-enhanced raman spectroscopy substrates: subnanomolar quantitative drug detection. ACS Sens. 4, 2988–2996. 10.1021/acssensors.9b01484 31565921 PMC6878213

[B11] de Nijs B. Kamp M. Szabó I. Barrow S. J. Benz F. Wu G.. (2017). Smart supramolecular sensing with cucurbit[n] urils: probing hydrogen bonding with SERS. Faraday Discuss. 205, 505–515. 10.1039/C7FD00147A 28932831

[B12] del Barrio J. Ryan S. T. J. Jambrina P. G. Rosta E. Scherman O. A. (2016). Light-regulated molecular trafficking in a synthetic water-soluble host. J. Am. Chem. Soc. 138, 5745–5748. 10.1021/jacs.5b11642 26876686

[B13] Ditchfield R. Hehre W. J. Pople J. A. (1971). Self-consistent molecular-orbital methods. IX. An extended Gaussian-type basis for molecular-orbital studies of organic molecules. J. Chem. Phys. 54, 724–728. 10.1063/1.1674902

[B14] Frisch M. J. Trucks G. W. Schlegel H. B. Scuseria G. E. Robb M. A. Cheeseman J. R. (2009). Gaussian 09 Revision E.01. Wallingford: Gaussian, Inc.

[B15] Grimme S. (2012). Supramolecular binding thermodynamics by dispersion-corrected density functional theory. Chem. A Eur. J. 18, 9955–9964. 10.1002/chem.201200497 22782805

[B16] Gschwend H. W. Hillman M. J. Kisis B. Rodebaugh R. K. (2002). Intramolecular Diels-Alder reactions. Synthesis of 3a-phenylisoindolines as analgetic templates. J. Org. Chem. 41, 104–110. 10.1021/jo00863a023

[B17] Harvey J. N. Himo F. Maseras F. Perrin L. (2019). Scope and challenge of computational methods for studying mechanism and reactivity in homogeneous catalysis. ACS Catal. 9, 6803–6813. 10.1021/acscatal.9b01537

[B18] Hehre W. J. Ditchfield R. Pople J. A. (1972). Self—consistent molecular orbital methods. XII. Further extensions of Gaussian—type basis sets for use in molecular orbital studies of organic molecules. J. Chem. Phys. 56, 2257–2261. 10.1063/1.1677527

[B19] Huang Y. Wang J. Xue S. F. Tao Z. Zhu Q. J. Tang Q. (2012). Determination of thiabendazole in aqueous solutions using a cucurbituril-enhanced fluorescence method. J. Incl. Phenom. Macrocycl. Chem. 72, 397–404. 10.1007/s10847-011-9999-1

[B20] Huang Z. Chen X. Wu G. Metrangolo P. Whitaker D. McCune J. A.. (2020). Host-enhanced phenyl-perfluorophenyl polar-π interactions. J. Am. Chem. Soc. 142, 7356–7361. 10.1021/jacs.0c02275 32248683 PMC7181256

[B21] Jon S. Y. Ko Y. H. Park S. H. Kim H. J. Kim K. (2001). A facile, stereoselective [2 + 2] photoreaction mediated by cucurbit[8]uril. Chem. Commun. 1, 1938–1939. 10.1039/b105153a 12240228

[B22] Kasera S. Biedermann F. Baumberg J. J. Scherman O. A. Mahajan S. (2012). Quantitative SERS using the sequestration of small molecules inside precise plasmonic nanoconstructs. Nano Lett. 12, 5924–5928. 10.1021/nl303345z 23088754

[B23] Lee J. Tofoleanu F. Pickard F. C. König G. Huang J. Damjanović A.. (2017). Absolute binding free energy calculations of CBClip host–guest systems in the SAMPL5 blind challenge. J. Comput. Aided. Mol. Des. 31, 71–85. 10.1007/s10822-016-9968-2 27677749 PMC5241186

[B24] Marenich A. V. Cramer C. J. Truhlar D. G. (2009). Universal solvation model based on solute electron density and on a continuum model of the solvent defined by the bulk dielectric constant and atomic surface tensions. J. Phys. Chem. B 113, 6378–6396. 10.1021/jp810292n 19366259

[B25] Muddana H. S. Varnado C. D. Bielawski C. W. Urbach A. R. Isaacs L. Geballe M. T.. (2012). Blind prediction of host-guest binding affinities: a new SAMPL3 challenge. J. Comput. Aided. Mol. Des. 26, 475–487. 10.1007/s10822-012-9554-1 22366955 PMC3383923

[B26] Muddana H. S. Yin J. Sapra N. V. Fenley A. T. Gilson M. K. (2014). Blind prediction of SAMPL4 cucurbit[7]uril binding affinities with the mining minima method. J. Comput. Aided Mol. Des. 28, 463–474. 10.1007/s10822-014-9726-2 24510191 PMC4053532

[B27] Olesińska M. Wu G. Gómez-Coca S. Antón-García D. Szabó I. Rosta E.. (2019). Modular supramolecular dimerization of optically tunable extended aryl viologens. Chem. Sci. 10, 8806–8811. 10.1039/C9SC03057C 31803453 PMC6849629

[B28] Ose T. Watanabe K. Mie T. Honma M. Watanabe H. Yao M.. (2003). Insight into a natural Diels-Alder reaction from the structure of macrophomate synthase. Nature 422, 185–189. 10.1038/nature01454 12634789

[B29] Palma A. Artelsmair M. Wu G. Lu X. Barrow S. J. Uddin N.. (2017). Cucurbit[7]uril as a supramolecular artificial enzyme for Diels–Alder reactions. Angew. Chem. Int. Ed. 56, 15688–15692. 10.1002/anie.201706487 29048713

[B30] Phillips J. C. Braun R. Wang W. Gumbart J. Tajkhorshid E. Villa E.. (2005). Scalable molecular dynamics with NAMD. J. Comput. Chem. 26, 1781–1802. 10.1002/jcc.20289 16222654 PMC2486339

[B31] Rad N. Danylyuk O. Sashuk V. (2019). Reversing chemoselectivity: simultaneous positive and negative catalysis by chemically equivalent rims of a cucurbit[7]uril host. Angew. Chemie Int. Ed. 58, 11340–11343. 10.1002/anie.201905027 31206979

[B32] Ren X. Yu Z. Wu Y. Liu J. Abell C. Scherman O. A. (2018). Cucurbit[7]uril-based high-performance catalytic microreactors. Nanoscale 10, 14835–14839. 10.1039/C8NR02900H 30051893 PMC6088369

[B33] Rizzi A. Murkli S. McNeill J. N. Yao W. Sullivan M. Gilson M. K.. (2018). Overview of the SAMPL6 host–guest binding affinity prediction challenge. J. Comput. Aided. Mol. Des. 32, 937–963. 10.1007/s10822-018-0170-6 30415285 PMC6301044

[B34] Senthilnathan D. Solomon R. V. Kiruthika S. Venuvanalingam P. Sundararajan M. (2018). Are cucurbiturils better drug carriers for bent metallocenes? Insights from theory. J. Biol. Inorg. Chem. 23, 413–423. 10.1007/s00775-018-1547-7 29502216

[B35] Shao Y. Gan Z. Epifanovsky E. Gilbert A. T. B. Wormit M. Kussmann J. (2015). Advances in molecular quantum chemistry contained in the Q-Chem 4 program package. Mol. Phys. 113, 184–215. 10.1080/00268976.2014.952696

[B36] Sigle D. O. Kasera S. Herrmann L. O. Palma A. de Nijs B. Benz F.. (2016). Observing single molecules complexing with cucurbit[7]uril through nanogap surface-enhanced raman spectroscopy. J. Phys. Chem. Lett. 7, 704–710. 10.1021/acs.jpclett.5b02535 26766205

[B37] Sin K. R. Ko S. G. Kim C. J. Pak S. H. Kim H. C. Kim C. U. (2020). Quantum chemical investigation on interaction of 5-fluorouracil with cucurbiturils. Monatsh. Chem. 151, 721–727. 10.1007/s00706-020-02599-1

[B38] Taylor R. W. Coulston R. J. Biedermann F. Mahajan S. Baumberg J. J. Scherman O. A. (2013). *In situ* SERS monitoring of photochemistry within a nanojunction reactor. Nano Lett. 13, 5985–5990. 10.1021/nl403164c 24188432 PMC3883114

[B39] Vanommeslaeghe K. MacKerell A. D. Jr. (2012). Automation of the CHARMM General Force Field (CGenFF) I: bond perception and atom typing. J. Chem. Inf. Model. 52, 3144–3154. 10.1021/ci300363c 23146088 PMC3528824

[B40] Wagner A. Ly K. H. Heidary N. Szabó I. Földes T. Assaf K. I. (2020). Host–guest chemistry meets electrocatalysis: cucurbit[6]uril on a Au surface as a hybrid system in CO_2_ reduction. ACS Catal. 10, 751–761. 10.1021/acscatal.9b04221 31929948 PMC6945685

[B41] Wang Y.-H. Cong H. Zhao F.-F. Xue S.-F. Tao Z. Zhu Q.-J. (2011). Selective catalysis for the oxidation of alcohols to aldehydes in the presence of cucurbit[8]uril. Catal. Commun. 12, 1127–1130. 10.1016/j.catcom.2011.03.029

[B42] Wu G. Olesińska M. Wu Y. Matak-Vinkovic D. Scherman O. A. (2017). Mining 2:2 complexes from 1:1 stoichiometry: formation of cucurbit[8]uril–diarylviologen quaternary complexes favored by electron-donating substituents. J. Am. Chem. Soc. 139, 3202–3208. 10.1021/jacs.6b13074 28198190

[B43] Wu G. Szabó I. Rosta E. Scherman O. A. (2019). Cucurbit[8]uril-mediated pseudo[2,3]rotaxanes. Chem. Commun. 55, 13227–13230. 10.1039/C9CC07144J 31631210

[B44] Yin H. Wang R. Wan J. Zheng Y. Ouyang D. Wang R. (2016). Molecular encapsulation of histamine H_2_-receptor antagonists by cucurbit[7]uril: an experimental and computational study. Molecules 21:1178. 10.3390/molecules21091178 27608003 PMC6274153

[B45] Zhang J. Coulston R. J. Jones S. T. Geng J. Scherman O. A. Abell C. (2012). One-step fabrication of supramolecular microcapsules from microfluidic droplets. Science 335, 690–694. 10.1126/science.1215416 22323815

[B46] Zheng L. Sonzini S. Ambarwati M. Rosta E. Scherman O. A. Herrmann A. (2015). Turning cucurbit[8]uril into a supramolecular nanoreactor for asymmetric catalysis. Angew. Chemie Int. Ed. 54, 13007–13011. 10.1002/anie.201505628 26383272 PMC4643185

