# Supplementary material for: Toward Understanding CB[7]-Based Supramolecular Diels-Alder Catalysis
Source: Front Chem. 2020 Nov 6;8:587084. doi: 10.3389/fchem.2020.587084 (PMC7677497; doi:10.3389/fchem.2020.587084)
Supplement: Supplementary file 1 [file Data_Sheet_1.pdf]

Toward Understanding CB[7]-Based Supramolecular Diels-Alder Catalysis

Supporting Information

Dénes Berta[1,2], István Szabó[2, ‡], Oren A. Scherman[3], Edina Rosta[1,2]

[1] Department of Physics and Astronomy, University College London; London WC1E 6BT (UK)

[2] Department of Chemistry, King's College London; London SE1 1DB (UK)

[3] Melville Laboratory for Polymer Synthesis, Department of Chemistry, University of Cambridge; Lensfield Road, Cambridge, CB2 1EW (UK)

‡ Present Address: ChemPass Ltd., 7 Záhony street, Budapest H-1031, Hungary.

CALCULATION OF GIBBS FREE ENERGIES

The reported relative stabilities are obtained from Gibbs free energies defined as

$G = E_0' + (G_0 - E_0) + (G_{sol} - E_0)$

where E_0' and E_0 are electronic energies computed with the 6-311++G(3df,3pd) and the 6-31G* basis sets, G_0 and G_{sol} denote gas-phase and solution-phase Gibbs free energies obtained from B3LYP-D3/6-31G* calculations.

ESTIMATION OF pK_a

We used Epik from the Schrödinger Suite to obtain an empirical estimate for the basicity of the amine moiety of substrates, predicting was 9.4±1. Based on the most stable conformers of **1a** with and without protonation, one can also estimate the pK_a:

$pK_a = \log(\exp(-\Delta G/RT))$

where $\Delta G = G(\mathbf{1a}H^+) - G(\mathbf{1a}) - G(H^+)$ is the reaction change in free energies. The solvated proton's free energy is taken from (D. Tissandier et al., 1998) as a value of -263.98 kcal/mol. Using this approach, the obtained pK_a is 10.8. Considering the experimental pH of 7.4, and assuming that the pK_a of the four substrates are similar, they are protonated under reaction conditions.

TESTING BASIS SETS AND FUNCTIONALS IN DFT CALCULATIONS

For benchmark purposes to evaluate how different functionals perform at different basis sets, we carried out calculations for the following models:

- Substrates **1a-d** in implicit solvent (RS, TS, PS)
- Substrate **1a** with a single explicit water (+implicit solvent) (RS, TS)
- Substrate **1a** complexed with CB[7] in implicit water (RS, TS)
- Substrates **1a-d** complexed with CB[7], with a single explicit water (+implicit solvent) (RS, TS, PS)

Results are reported in Tables S1 and S2.

Basis sets

We tested the variation in electronic energies (E_0') with a series of Pople basis sets using B3LYP hybrid functional. The optimizations, thermochemical and implicit solvent corrections were added in all cases based on the B3LYP-D3/6-31G* calculations. The obtained barriers are summarized in Table S1.

Table S1. Reaction free energy barriers ($\Delta G^‡$) in kcal/mol based on electronic energies (E_0') calculated with different basis sets.

model	**1a**	**1b**	**1c**	**1d**	**1a**+water	**1a**+CB[7]	**1a**+CB[7]	**1b**+CB[7]	**1c**+CB[7]	**1d**+CB[7]
experimental	28.7	28.6	26.8	26.7	28.7	23.6	23.6	23.0	22.5	22.7
6-311++G(3df,3pd)	28.6	29.5	26.9	26.9	28.4	28.6	24.5	26.6	22.2	21.7
6-31G*	26.2	27.2	24.5	23.8	25.9	25.5	22.7	23.5	19.5	19.7
6-31G**	26.3	27.3	24.6	23.8	26.1	25.8	22.9	23.7	19.7	19.9

6-31+G*	26.8	27.7	24.9	24.5	26.5	26.0	22.6	24.4	19.7	20.0
6-31+G**	27.0	27.8	25.0	24.6	26.8	26.4	23.0	24.7	20.0	20.3
6-31++G*	26.8	27.8	24.9	24.7	26.5	26.1	22.7	24.5	19.7	20.0
6-31++G**	27.1	27.9	25.1	24.8	26.8	26.5	22.9	24.8	20.0	20.2

Functionals and semiempiricals

We also tested a few Minnesota functionals (Zhao and Truhlar, 2008; Peverati and Truhlar, 2011a, 2011b, 2012) available in Gaussian 09 to calculate electronic energies, using the 6-31G** basis. They produce slightly lower barriers in general, although the behavior of **1b** with CB[7] is more correctly described (Table S2). In an attempt to speed up calculations, we expanded the benchmark to semiempirical methods am1 and pm6.

Table S2. Reaction free energy barriers ($\Delta G^‡$) in kcal/mol based on electronic energies (E_0') calculated with different DFT functionals or semiempirical methods.

model	**1a**	**1b**	**1c**	**1d**	**1a**+water	**1a**+CB[7]	**1a**+CB[7]	**1b**+CB[7]	**1c**+CB[7]	**1d**+CB[7]
experimental	28.7	28.6	26.8	26.7	28.7	23.6	23.6	23.0	22.5	22.7
M06-2X	24.6	25.1	22.9	22.2	24.5	24.8	22.4	21.8	20.0	20.3
M11L	23.0	24.0	21.8	20.9	23.1	23.2	21.9	19.9	18.2	18.1
MN12L	25.4	26.3	24.0	23.2	24.9	26.2	24.7	22.6	21.9	22.2
M11	24.0	24.5	22.3	21.5	23.7	24.6	22.0	21.1	20.2	20.5
MN12SX	24.4	25.6	23.0	22.2	25.4	25.4	24.1	22.4	20.7	20.9
am1	33.7	36.3	34.0	35.1	31.9	28.2	23.1	32.8	25.3	24.9
pm6	30.7	32.4	29.3	29.5	31.0	32.0	23.3	32.2	24.8	25.0

CALCULATIONS WITH 2 WATER MOLECULES

We tested the effect of a few explicit water molecules on the barrier of the cycloaddition of **1a**. The issue with adding a defined number of explicit water molecules is that the number of possible arrangements in geometry is quickly increasing. We have tested several possible conformers and have a standard deviation of 3.1 kcal/mol for the barrier depending on the water placement. The best conformer has a barrier of 26.0 kcal/mol. The ambiguity is similar adding further water molecules to the CB[7] caged system, thus we decided to eliminate the problem using a water box in QM/MM calculations as discussed in the main text.

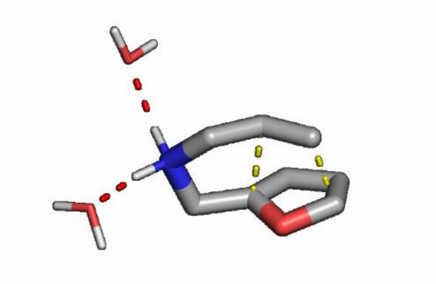

Figure S1. Example TS of **1a** accompanied by two water molecules.

STRUCTURES WITHOUT EXPLICIT WATER

The complexation of the substrates (studied on the example of **1a**) is driven by the H-bonding interaction between the protonated amine and the carbonyl moieties of the CB[7], slightly turning the substrate in the TS as depicted in Figure S1.

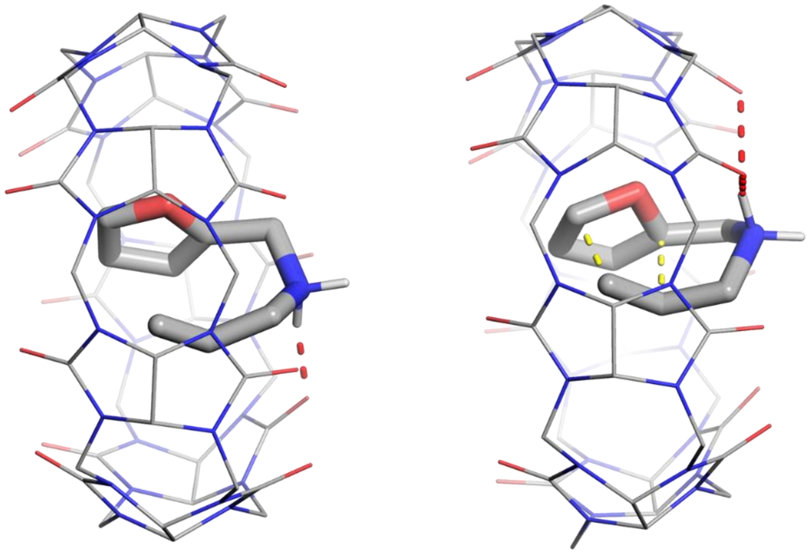

Figure S2. RS (left) and TS (right) of **1a** in complex with CB[7], without explicit water. Non-polar hydrogens are hidden for clarity.

QM/MM RESULTS OF **1b**

The catalytic effect on different substrates are reproduced satisfactorily by DFT as shown in Table 2, apart from **1b**, for which the calculated effect (2.9 kcal/mol) underestimates the experimental value (6.1 kcal/mol). We employed the same QM/MM minimization approach as discussed in the manuscript (Figure 4). The effect of CB[7] is again more precisely reproduced by the QM/MM calculations (6.0 kcal/mol, Figure S3).

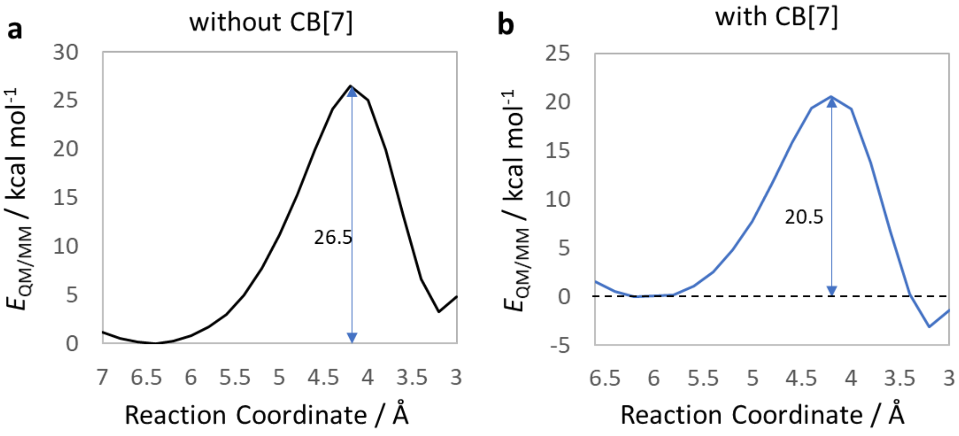

Figure S3. Results of QM/MM potential energy scans **a)** without CB[7] and **b)** with CB[7] for the reaction of **1b**. The reaction coordinate is the sum of the distances of C-C inner and C-C outer bonds. Reaction barriers are shown in kcal/mol, indicated by vertical arrows in the profiles.

RESULTS FOR NITRO-FURILAMINE **1e**

We performed calculations in implicit solvent and QM/MM calculations for an additional substrate **1e** featuring a nitro group in the 5 position of the furan ring.

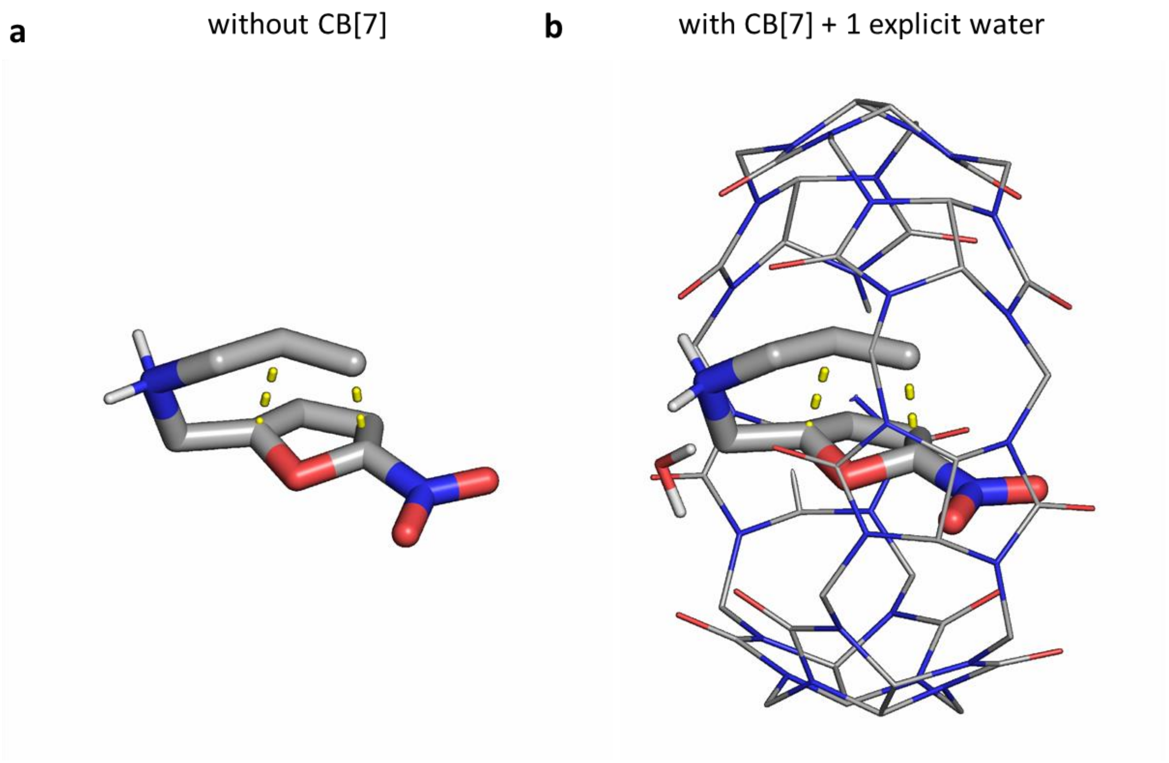

Figure S4. TS geometries of the Diels-Alder reaction of substrate **1e a)** without CB[7] and **b)** with CB[7] and one explicit water molecule. Non-polar hydrogens are hidden for clarity. Forming bonds are depicted with yellow dashes. Barriers are 25.5 and 21.4 kcal/mol, respectively.

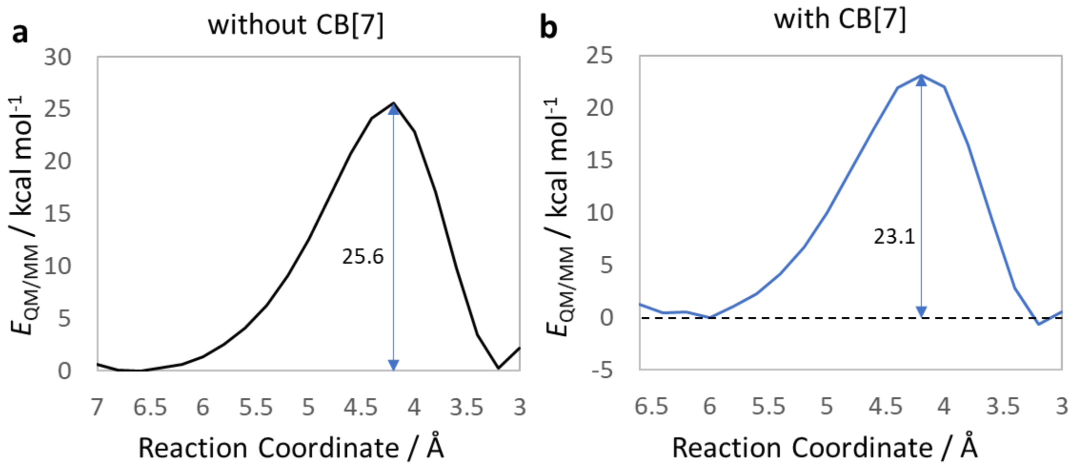

Figure S5. Results of QM/MM potential energy scans **a)** without CB[7] and **b)** with CB[7] for the reaction of **1e**. The reaction coordinate is the sum of the distances of C-C inner and C-C outer bonds. Reaction barriers are shown in kcal/mol, indicated by vertical arrows in the profiles.

POINT CHARGE MAPPING

We carried out the same analysis of the influence of a point charge with a +1 probe. The results are largely complementary as one would expect, however there are regions (i.e. near the furan ring) where both charges are unfavorable, which illustrates the reactions preference for the hydrophobic cavity.

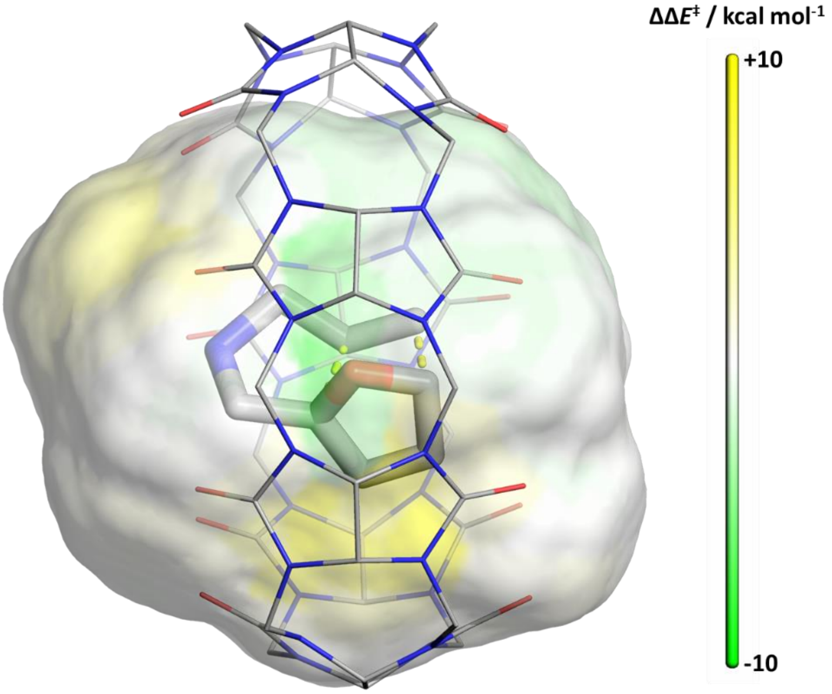

Figure S6. Map of reaction barrier changes upon the addition of a probe +1 point charge (calculated at B3LYP/6-31+G*). CB[7] is overlaid with the results to highlight the area shielded from the polar environment.

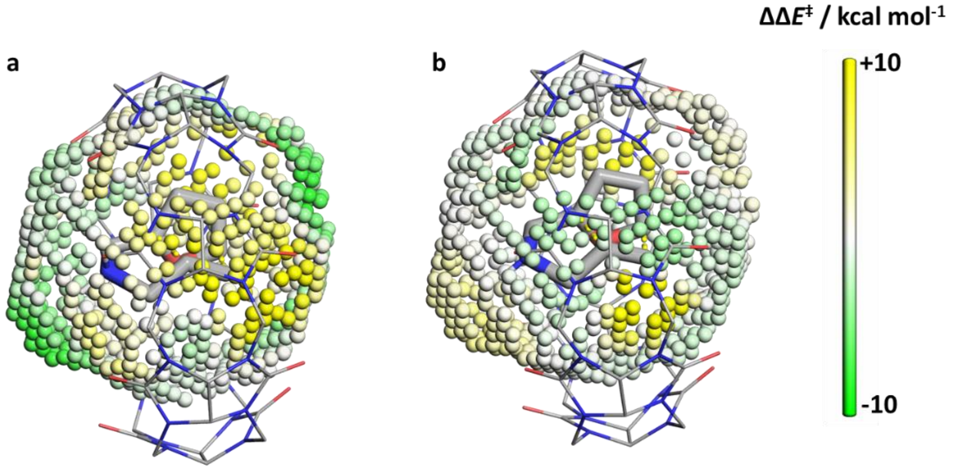

Figure S7. Representation of the point charge analysis by displaying each node as a sphere with a probe charge of **a)** -1 **b)** +1.

REFERENCES

D. Tissandier, M., A. Cowen, K., Yong Feng, W., Gundlach, E., H. Cohen, M., D. Earhart, A., et al. (1998). The proton's absolute aqueous enthalpy and Gibbs free rnergy of solvation from cluster-ion solvation data. *J. Phys. Chem. A* 102, 7787–7794. doi:10.1021/jp982638r.

Peverati, R., and Truhlar, D. G. (2011a). Improving the accuracy of hybrid meta-GGA density functionals by range separation. *J. Phys. Chem. Lett.* 2, 2810–2817. doi:10.1021/jz201170d.

Peverati, R., and Truhlar, D. G. (2011b). M11-L: A local density functional that provides improved accuracy for electronic structure calculations in chemistry and physics. *J. Phys. Chem. Lett.* 3, 117–124. doi:10.1021/jz201525m.

Peverati, R., and Truhlar, D. G. (2012). Screened-exchange density functionals with broad accuracy for chemistry and solid-state physics. *Phys. Chem. Chem. Phys.* 14, 16187–16191. doi:10.1039/c2cp42576a.

Zhao, Y., and Truhlar, D. G. (2008). The M06 suite of density functionals for main group thermochemistry, thermochemical kinetics, noncovalent interactions, excited states, and transition elements: Two new functionals and systematic testing of four M06-class functionals and 12 other functionals. *Theor. Chem. Acc.* 120, 215–241. doi:10.1007/s00214-007-0310-x.

DATA

Table S3. Raw energetic data calculated at B3LYP-D3/6-31G*//B3LYP-D3/6-311++G(3df,3pd) level of theory. Values are in Hartree.

	E_0	G_0	G_{sol}	E_0'
**w/o CB w/o water**				
1a RS	-441.783493	-441.626896	-441.875423	-441.939361
1a TS	-441.742324	-441.580789	-441.838680	-441.894364
1a PS	-441.781740	-441.616551	-441.883425	-441.931282
1b RS	-481.112113	-480.930265	-481.201472	-481.280660
1b TS	-481.069805	-480.882408	-481.163615	-481.234821
1b PS	-481.108329	-480.917288	-481.207634	-481.271060
1c RS	-901.372609	-901.228060	-901.466793	-901.561646
1c TS	-901.332037	-901.182372	-901.432841	-901.517347
1c PS	-901.373995	-901.220892	-901.481829	-901.556389
1d RS	-3012.881598	-3012.738601	-3012.976106	-3015.480004
1d TS	-3012.842072	-3012.694207	-3012.943094	-3015.435526
1d PS	-3012.883935	-3012.732597	-3012.991528	-3015.474631
**w/o CB with water**				
1a+wat RS	-518.220909	-518.045723	-518.309810	-518.425152
1a+wat TS	-518.183414	-518.003577	-518.273141	-518.383776
**with CB w/o water**				
1a+CB[7] RS	-4654.400205	-4653.328430	-4654.621921	-4655.962894
1a+CB[7] TS	-4654.359058	-4653.286451	-4654.582168	-4655.916738
**with CB with water**				
1a+CB[7]+wat RS	-4730.849234	-4729.752425	-4731.065875	-4732.453861
1a+CB[7]+wat TS	-4730.811326	-4729.713778	-4731.030474	-4732.412972
1a+CB[7]+wat PS	-4730.852029	-4729.750588	-4731.073261	-4732.452141
1b+CB[7]+wat RS	-4770.179062	-4769.055765	-4770.399903	-4771.798595
1b+CB[7]+wat TS	-4770.143669	-4769.017200	-4770.365566	-4771.758373
1b+CB[7]+wat PS	-4770.182021	-4769.051748	-4770.407950	-4771.794350
1c+CB[7]+wat RS	-5190.441926	-5189.354741	-5190.661327	-5192.082641
1c+CB[7]+wat TS	-5190.407293	-5189.319482	-5190.630807	-5192.043700
1c+CB[7]+wat PS	-5190.449696	-5189.357676	-5190.678635	-5192.083015
1d+CB[7]+wat RS	-7301.961269	-7300.874265	-7302.176570	-7305.999702
1d+CB[7]+wat TS	-7301.926631	-7300.840650	-7302.144101	-7305.962002
1d+CB[7]+wat PS	-7301.968637	-7300.877411	-7302.190430	-7306.000641

Table S4. Raw energetic data calculated with B3LYP-D3 and the selected basis set. Values are in Hartree.

basis set	6-31G**	6-31+G*	6-31+G**	6-31++G*	6-31++G**
**w/o CB w/o water**					
1a RS	-441.804121	-441.794630	-441.814837	-441.795061	-441.815239
1a TS	-441.762661	-441.752483	-441.772259	-441.752850	-441.772587
1a PS	-441.801704	-441.792063	-441.811402	-441.792416	-441.811703
1b RS	-481.135326	-481.123894	-481.146550	-481.124330	-481.146959
1b TS	-481.092933	-481.080845	-481.103274	-481.081182	-481.103580
1b PS	-481.131193	-481.119596	-481.141682	-481.119936	-481.141986
1c RS	-901.391590	-901.384053	-901.402606	-901.384461	-901.402993
1c TS	-901.350883	-901.342890	-901.361197	-901.343229	-901.361504
1c PS	-901.392565	-901.385026	-901.403015	-901.385364	-901.403314
1d RS	-3012.900608	-3012.917025	-3012.935594	-3012.918756	-3012.937301

1d TS	-3012.861035	-3012.876379	-3012.894791	-3012.877761	-3012.896140
1d PS	-3012.902629	-3012.918456	-3012.936551	-3012.919781	-3012.937839
**w/o CB w/ water**					
1a+wat RS	-518.252416	-518.241052	-518.272524	-518.241483	-518.272872
1a+wat TS	-518.214637	-518.202667	-518.233619	-518.203102	-518.233992
**w/ CB w/o water**					
1a+CB[7] RS	-4654.476145	-4654.520165	-4654.594163	-4654.521838	-4654.595844
1a+CB[7] TS	-4654.434486	-4654.478182	-4654.551516	-4654.479674	-4654.553041
**w/ CB w/ water**					
1a+CB[7]+wat RS	-4730.935775	-4730.973778	-4731.058035	-4730.975451	-4731.059574
1a+CB[7]+wat TS	-4730.897532	-4730.935926	-4731.019669	-4730.937586	-4731.021331
1a+CB[7]+wat PS	-4730.937823	-4730.977682	-4731.060998	-4730.979251	-4731.062575
1b+CB[7]+wat RS	-4770.268271	-4770.305674	-4770.392271	-4770.307547	-4770.393977
1b+CB[7]+wat TS	-4770.232598	-4770.268943	-4770.354992	-4770.270592	-4770.356650
1b+CB[7]+wat PS	-4770.270569	-4770.307566	-4770.393175	-4770.309207	-4770.394826
1c+CB[7]+wat RS	-5190.526821	-5190.568475	-5190.650855	-5190.570113	-5190.652497
1c+CB[7]+wat TS	-5190.491877	-5190.533640	-5190.615555	-5190.535250	-5190.617169
1c+CB[7]+wat PS	-5190.533895	-5190.576254	-5190.657732	-5190.577833	-5190.659195
1d+CB[7]+wat RS	-7302.046323	-7302.124814	-7302.207416	-7302.127873	-7302.210509
1d+CB[7]+wat TS	-7302.011429	-7302.089710	-7302.171874	-7302.092885	-7302.175061
1d+CB[7]+wat PS	-7302.053021	-7302.132454	-7302.214202	-7302.135512	-7302.217272

Table S5. Raw energetic data calculated with different functionals using the 6-31G** basis set. Values are in Hartree.

method	M06-2X	M11L	MN12L	M11	MN12SX
**w/o CB w/o water**					
1a RS	-441.583045	-441.648586	-441.411831	-441.523751	-441.339749
1a TS	-441.544391	-441.612513	-441.371881	-441.486043	-441.301311
1a PS	-441.591465	-441.659567	-441.420872	-441.534368	-441.348115
1b RS	-480.889140	-480.968463	-480.700838	-480.822427	-480.618485
1b TS	-480.850246	-480.931322	-480.659976	-480.784495	-480.578815
1b PS	-480.896367	-480.977384	-480.707667	-480.831521	-480.624213
1c RS	-901.143071	-901.194084	-900.946376	-901.082553	-900.852297
1c TS	-901.105122	-901.157826	-900.906690	-901.045510	-900.814198
1c PS	-901.155238	-901.207324	-900.958337	-901.096725	-900.863639
1d RS	-3012.754601	-3012.382400	-3011.879355	-3012.329678	-3011.392511
1d TS	-3012.717634	-3012.347458	-3011.840803	-3012.293753	-3011.355477
1d PS	-3012.767611	-3012.397323	-3011.892433	-3012.345050	-3011.404734
**w/o CB w/ water**					
1a+wat RS	-517.994796	-518.070073	-517.711185	-517.943454	-517.800865
1a+wat TS	-517.959521	-518.037051	-517.675256	-517.909517	-517.764138
**w/ CB w/o water**					
1a+CB[7] RS	-4652.598610	-4652.793412	-4650.256256	-4652.139819	-4650.857970
1a+CB[7] TS	-4652.558481	-4652.755804	-4650.213909	-4652.100050	-4650.816919
**w/ CB w/ water**					
1a+CB[7]+wat RS	-4729.022762	-4729.220336	-4726.638735	-4728.571392	-4727.255970
1a+CB[7]+wat TS	-4728.985341	-4729.183661	-4726.597670	-4728.534499	-4727.215873
1a+CB[7]+wat PS	-4729.032423	-4729.230625	-4726.644459	-4728.581924	-4727.264747
1b+CB[7]+wat RS	-4768.326280	-4768.536731	-4765.909432	-4767.865093	-4766.540408
1b+CB[7]+wat TS	-4768.293718	-4768.507202	-4765.875529	-4767.833595	-4766.506839
1b+CB[7]+wat PS	-4768.338433	-4768.552993	-4765.919390	-4767.878425	-4766.553517
1c+CB[7]+wat RS	-5188.584971	-5188.767645	-5186.150993	-5188.131762	-5186.791922
1c+CB[7]+wat TS	-5188.549583	-5188.735092	-5186.112613	-5188.096111	-5186.755391

1c+CB[7]+wat PS	-5188.598911	-5188.784909	-5186.161033	-5188.145821	-5186.806611
1d+CB[7]+wat RS	-7300.208024	-7299.966910	-7296.704779	-7299.390542	-7297.736514
1d+CB[7]+wat TS	-7300.172458	-7299.934814	-7296.666187	-7299.354699	-7297.700088
1d+CB[7]+wat PS	-7300.221191	-7299.984613	-7296.714266	-7299.404223	-7297.751032

Table S6. Raw energetic data calculated with semiempirical methods am1 and pm6. Values are in Hartree.

method	am1	pm6
**w/o CB w/o water**		
1a RS	0.281213	0.244996
1a TS	0.334477	0.293473
1a PS	0.278226	0.230915
1b RS	0.269233	0.225270
1b TS	0.326018	0.275743
1b PS	0.271298	0.213612
1c RS	0.282161	0.240963
1c TS	0.337849	0.289150
1c PS	0.282475	0.224287
1d RS	0.300861	0.258740
1d TS	0.358517	0.307464
1d PS	0.304281	0.244212
**w/o CB w/ water**		
1a+wat RS	0.178317	0.142831
1a+wat TS	0.225392	0.188351
**w/ CB w/o water**		
1a+CB[7] RS	0.545954	-0.229719
1a+CB[7] TS	0.591509	-0.178085
**w/ CB w/ water**		
1a+CB[7]+wat RS	0.439981	-0.330705
1a+CB[7]+wat TS	0.478538	-0.291880
1a+CB[7]+wat PS	0.427716	-0.355254
1b+CB[7]+wat RS	0.429918	-0.351822
1b+CB[7]+wat TS	0.480140	-0.302570
1b+CB[7]+wat PS	0.428370	-0.363592
1c+CB[7]+wat RS	0.447677	-0.334771
1c+CB[7]+wat TS	0.491412	-0.291728
1c+CB[7]+wat PS	0.439896	-0.356239
1d+CB[7]+wat RS	0.473699	-0.319112
1d+CB[7]+wat TS	0.516646	-0.276100
1d+CB[7]+wat PS	0.468636	-0.338448

Table S7. QM/MM energetic data calculated in reaction coordinate scans of **1a**, last iteration.

**coordinate**	**w/o CB[7]**	**w/ CB[7]**
7.0	-300246.682	
6.8	-300247.129	
6.6	-300247.433	-300079.834
6.4	-300247.626	-300080.953
6.2	-300247.365	-300081.685
6.0	-300247.006	-300082.013
5.8	-300246.096	-300081.682
5.6	-300244.641	-300080.926
5.4	-300242.688	-300079.426

5.2	-300240.023	-300077.173
5.0	-300236.622	-300074.039
4.8	-300232.378	-300070.206
4.6	-300227.766	-300066.243
4.4	-300223.751	-300062.712
4.2	-300221.743	-300061.197
4.0	-300223.421	-300063.06
3.8	-300228.432	-300068.064
3.6	-300235.282	-300075.065
3.4	-300242.01	-300081.755
3.2	-300245.899	-300085.964
3.0	-300243.856	-300084.384

Table S8. QM/MM energetic data calculated in reaction coordinate scans of **1b**, last iteration.

**coordinate**	**w/o CB[7]**	**w/ CB[7]**
7.0	-325097.570	
6.8	-325098.259	
6.6	-325098.586	-325033.123
6.4	-325098.734	-325034.083
6.2	-325098.514	-325034.591
6.0	-325097.922	-325034.555
5.8	-325097.005	-325034.413
5.6	-325095.727	-325033.562
5.4	-325093.753	-325032.077
5.2	-325091.056	-325029.781
5.0	-325087.569	-325026.863
4.8	-325083.496	-325022.905
4.6	-325078.784	-325018.756
4.4	-325074.608	-325015.185
4.2	-325072.250	-325014.067
4.0	-325073.690	-325015.283
3.8	-325078.842	-325020.879
3.6	-325085.650	-325027.854
3.4	-325092.113	-325034.362
3.2	-325095.524	-325037.764
3.0	-325093.972	-325035.987

CARTESIAN COORDINATES

```
  22
1a RS
 C 0.936356 -0.140954 0.547934
 C 1.369108 1.152208 0.499375
 C 2.598245 1.136128 -0.235856
 C 2.813779 -0.160141 -0.592888
 O 1.809430 -0.960047 -0.123894
 H 0.885620 2.009191 0.948423
 H 3.234396 1.979769 -0.461773
 H 3.592175 -0.661619 -1.147888
 C -0.238350 -0.817096 1.128347
 H 0.030716 -1.648780 1.787385
 H -0.883319 -0.116592 1.659065
 N -1.086467 -1.445777 0.007838
 H -0.463683 -2.053716 -0.538831
 C -1.758854 -0.438917 -0.932995
 H -0.952917 0.186999 -1.321220
 H -2.177814 -1.035303 -1.749467
 C -2.802035 0.355573 -0.209886
 C -2.678861 1.662636 0.030740
 H -1.800679 2.223472 -0.282103
 H -3.465002 2.223746 0.526796
 H -3.703769 -0.177951 0.089474
 H -1.803076 -2.052726 0.423523
  22
1a TS
 C 0.074208 -0.877904 0.056208
 C 1.104470 -1.224068 -0.858954
 C 2.206352 -0.507936 -0.457515
 C 1.793174 0.260052 0.678262
 O 0.640378 -0.325784 1.174116
 H 0.985084 -1.822206 -1.752849
 H 3.158409 -0.416060 -0.962361
 H 2.442240 0.685129 1.434388
 C -1.305720 -1.392928 0.257714
 H -1.537403 -2.263596 -0.357286
 H -1.485127 -1.624504 1.309603
 N -2.299594 -0.276599 -0.124159
 H -3.180025 -0.369671 0.397228
 C -1.675815 1.124143 0.058949
 H -2.433865 1.822018 -0.314818
 H -1.548062 1.264628 1.134422
 C -0.375404 1.156857 -0.671045
 C 0.771863 1.788708 -0.139967
 H 0.667911 2.345530 0.789007
 H 1.473811 2.235095 -0.837269
 H -0.443539 1.042942 -1.751251
 H -2.540987 -0.380489 -1.115681
  22
1a PS
 C -0.009856 -0.641965 -0.024658
 C 1.185635 -1.310383 -0.675540
 C 2.232895 -0.590170 -0.260751
 C 1.666079 0.498752 0.641080
 O 0.517221 -0.165466 1.218208
 H 1.148539 -2.114365 -1.400105
 H 3.268648 -0.671946 -0.566341
 H 2.308714 0.904746 1.420197
 C -1.345729 -1.319783 0.185975
```

H -1.567233 -2.085152 -0.558313
H -1.436181 -1.734985 1.190067
N -2.376970 -0.184279 0.023747
H -3.038513 -0.159479 0.805854
C -1.611442 1.146556 -0.104157
H -2.228503 1.842296 -0.674980
H -1.468047 1.511620 0.913106
C -0.285309 0.740846 -0.739713
C 0.952554 1.556587 -0.280687
H 0.668968 2.436029 0.304331
H 1.574207 1.885846 -1.115058
H -0.374799 0.649303 -1.825738
H -2.936408 -0.342897 -0.819592
25
1b RS
C 0.929742 -0.141172 0.517381
C 1.347105 1.156525 0.465818
C 2.571983 1.152895 -0.272161
C 2.816914 -0.140996 -0.637474
O 1.812941 -0.948993 -0.159520
H 0.855172 2.007948 0.916709
H 3.197695 2.004195 -0.498907
C -0.235160 -0.828377 1.097108
H 0.038771 -1.675702 1.734029
H -0.876057 -0.140318 1.648599
N -1.099770 -1.437313 -0.027795
H -0.480593 -2.031110 -0.593361
C -1.782893 -0.412074 -0.938255
H -0.982165 0.227627 -1.314753
H -2.204962 -0.988784 -1.767187
C -2.824989 0.359374 -0.188804
C -2.706509 1.660531 0.083612
H -1.832945 2.233082 -0.220711
H -3.491673 2.205122 0.599230
H -3.721834 -0.185953 0.103964
H -1.810011 -2.054740 0.383290
C 3.904385 -0.804587 -1.402018
H 4.633634 -0.059992 -1.729324
H 4.421683 -1.549053 -0.785929
H 3.513165 -1.318336 -2.288029
25
1b TS
C 0.071522 -0.854133 0.049735
C 1.111507 -1.201927 -0.857132
C 2.204046 -0.482508 -0.446410
C 1.803039 0.273813 0.709052
O 0.634596 -0.317558 1.178641
H 0.997289 -1.789330 -1.758951
H 3.158036 -0.382444 -0.946908
C -1.300942 -1.391768 0.246942
H -1.522322 -2.255469 -0.381437
H -1.476317 -1.642181 1.295130
N -2.311163 -0.282145 -0.115931
H -3.181081 -0.380021 0.421238
C -1.687056 1.117902 0.059374
H -2.442887 1.817415 -0.315089
H -1.553781 1.262356 1.133651
C -0.387065 1.137362 -0.674001
C 0.755777 1.789830 -0.157507
H 0.650420 2.358209 0.763994
H 1.452276 2.231156 -0.863424

H -0.463656 1.019233 -1.753611
H -2.569960 -0.385465 -1.102943
C 2.708661 0.846281 1.755181
H 3.219157 0.047201 2.303391
H 3.467373 1.476889 1.283179
H 2.145852 1.451977 2.470138
25
1b PS
C -0.013853 -0.642154 -0.022298
C 1.181988 -1.309830 -0.671648
C 2.225127 -0.585265 -0.257638
C 1.671346 0.506514 0.658463
O 0.507189 -0.167812 1.219915
H 1.146839 -2.113372 -1.397124
H 3.261734 -0.663291 -0.563056
C -1.350278 -1.321901 0.180174
H -1.569264 -2.082383 -0.569962
H -1.444798 -1.743288 1.181211
N -2.381798 -0.185604 0.021059
H -3.042095 -0.161611 0.804053
C -1.614946 1.145074 -0.105433
H -2.231561 1.841964 -0.675304
H -1.470803 1.507884 0.912449
C -0.288928 0.738584 -0.739777
C 0.946746 1.551151 -0.279023
H 0.663284 2.433194 0.303659
H 1.571650 1.879436 -1.111593
H -0.378881 0.645799 -1.825917
H -2.942280 -0.342389 -0.821787
C 2.567091 1.065833 1.734881
H 2.960187 0.260803 2.361623
H 3.409876 1.599001 1.283562
H 2.013880 1.766303 2.367691
22
1c RS
C 0.931953 -0.136706 0.543599
C 1.370678 1.154408 0.502559
C 2.598117 1.149018 -0.231771
C 2.805463 -0.149616 -0.596728
O 1.807386 -0.956093 -0.137211
H 0.890374 2.010186 0.957628
H 3.240660 1.986883 -0.456821
C -0.237069 -0.821097 1.122106
H 0.036946 -1.652985 1.779026
H -0.882717 -0.123535 1.656123
N -1.087097 -1.452054 0.003224
H -0.466998 -2.061987 -0.544512
C -1.764128 -0.447964 -0.937698
H -0.960316 0.175987 -1.333449
H -2.187580 -1.047543 -1.749503
C -2.802900 0.349437 -0.211641
C -2.680836 1.658435 0.018809
H -1.806691 2.219338 -0.305088
H -3.464678 2.221360 0.516425
H -3.701476 -0.183688 0.097748
H -1.801812 -2.058635 0.422929
Cl 4.039672 -0.902202 -1.496056
22
1c TS
C 0.074247 -0.859657 0.052317
C 1.112362 -1.207677 -0.855235

C 2.217313 -0.506515 -0.443594
C 1.794804 0.260615 0.692895
O 0.638625 -0.319609 1.182011
H 0.995114 -1.793708 -1.757610
H 3.179266 -0.413125 -0.927271
C -1.301641 -1.386087 0.251961
H -1.523534 -2.253978 -0.370594
H -1.476415 -1.630048 1.301845
N -2.308150 -0.276870 -0.117774
H -3.179170 -0.373927 0.418118
C -1.688076 1.122926 0.058858
H -2.443392 1.822605 -0.315843
H -1.554684 1.268869 1.132968
C -0.388732 1.151949 -0.677458
C 0.749412 1.800613 -0.158341
H 0.660117 2.346500 0.777705
H 1.463333 2.229996 -0.853534
H -0.461356 1.027245 -1.756548
H -2.565754 -0.382808 -1.105065
Cl 2.863161 0.902452 1.894705
22
1c PS
C -0.013119 -0.641386 -0.029903
C 1.184753 -1.314233 -0.672581
C 2.232322 -0.597880 -0.256516
C 1.655815 0.495713 0.635107
O 0.513030 -0.164793 1.216958
H 1.150047 -2.119418 -1.396054
H 3.273642 -0.665092 -0.542894
C -1.348301 -1.319623 0.182067
H -1.568605 -2.081308 -0.566552
H -1.435906 -1.741237 1.183708
N -2.379445 -0.183768 0.027362
H -3.032430 -0.157265 0.816814
C -1.615670 1.147691 -0.107739
H -2.233053 1.840270 -0.681825
H -1.471564 1.518402 0.907409
C -0.288809 0.742132 -0.743380
C 0.945431 1.557810 -0.280487
H 0.669866 2.426716 0.321963
H 1.579262 1.889829 -1.103551
H -0.374330 0.651516 -1.829763
H -2.948630 -0.343424 -0.809410
Cl 2.721255 1.163408 1.871198
22
1d RS
C 0.929696 -0.136151 0.548083
C 1.371098 1.154296 0.510818
C 2.593921 1.147426 -0.230614
C 2.797335 -0.149261 -0.604042
O 1.798677 -0.954638 -0.141410
H 0.895390 2.009305 0.972183
H 3.237412 1.984350 -0.456053
C -0.239270 -0.820790 1.126533
H 0.034230 -1.652495 1.783979
H -0.886554 -0.123775 1.659270
N -1.085569 -1.452770 0.005705
H -0.462392 -2.061390 -0.540055
C -1.760261 -0.449503 -0.937611
H -0.955325 0.173514 -1.332500
H -2.182436 -1.049784 -1.749562

C -2.799897 0.349551 -0.214638
C -2.676981 1.658687 0.014548
H -1.801475 2.218186 -0.308082
H -3.461190 2.222976 0.510041
H -3.699509 -0.182423 0.093709
H -1.800821 -2.060295 0.423026
Br 4.137900 -0.959100 -1.596186
22
1d TS
C 0.073281 -0.863021 0.053458
C 1.111328 -1.211188 -0.854403
C 2.214263 -0.505140 -0.445061
C 1.791216 0.262916 0.689603
O 0.637586 -0.320691 1.182322
H 0.995059 -1.799973 -1.755163
H 3.176409 -0.410456 -0.927967
C -1.303321 -1.387188 0.253058
H -1.526263 -2.256457 -0.367241
H -1.479556 -1.627656 1.303517
N -2.307711 -0.277471 -0.120863
H -3.181211 -0.374403 0.411085
C -1.688304 1.122905 0.057732
H -2.444331 1.821787 -0.317247
H -1.556662 1.267820 1.132204
C -0.388287 1.153789 -0.676338
C 0.750285 1.798539 -0.151712
H 0.657766 2.343789 0.784230
H 1.465760 2.231011 -0.843417
H -0.458494 1.031507 -1.755813
H -2.560696 -0.383420 -1.109333
Br 2.967275 0.974236 1.986171
22
1d PS
C -0.014305 -0.642568 -0.030732
C 1.184696 -1.315487 -0.671864
C 2.231205 -0.596930 -0.255660
C 1.653152 0.493894 0.634091
O 0.511421 -0.166032 1.217717
H 1.151617 -2.121286 -1.394762
H 3.273251 -0.663593 -0.539037
C -1.349752 -1.320291 0.180501
H -1.570532 -2.081472 -0.568486
H -1.437707 -1.742277 1.181973
N -2.380286 -0.183820 0.026365
H -3.033527 -0.157625 0.815583
C -1.615691 1.147327 -0.107614
H -2.232777 1.840705 -0.681061
H -1.471437 1.516944 0.907916
C -0.289090 0.741864 -0.743212
C 0.945545 1.557288 -0.277622
H 0.668443 2.426701 0.323185
H 1.580405 1.889738 -1.099736
H -0.373938 0.652694 -1.829734
H -2.949134 -0.342547 -0.810774
Br 2.822419 1.226555 1.972491
25
1a+wat RS
C 0.526548 0.999384 -1.411985
C -0.747461 0.650208 -0.755894
H 1.048411 1.789335 -0.866394
H 0.370208 1.322761 -2.443988

C -1.931560 0.136944 -1.205696
C -2.757342 -0.038232 -0.052977
H -2.191840 -0.080119 -2.233136
H -3.770457 -0.412843 -0.024686
C -2.017630 0.385195 1.011552
O -0.790498 0.813446 0.603142
H -2.218438 0.471826 2.068673
N 1.511423 -0.172109 -1.487864
C 1.861508 -0.844159 -0.160274
H 2.399868 0.164836 -1.924714
H 1.114567 -0.882960 -2.111206
H 1.861034 -0.061260 0.601053
H 2.886990 -1.203927 -0.295211
C 0.923506 -1.963137 0.177550
C 0.251008 -2.045540 1.325553
H 0.864690 -2.773486 -0.550117
H 0.307314 -1.265883 2.079676
H -0.367867 -2.906889 1.555906
O 4.030104 0.565036 -2.367103
H 4.494770 0.261344 -3.162360
H 4.551731 1.309942 -2.030370
25
1a+wat TS
C -0.926671 -1.168693 -0.302742
C 0.512007 -0.832984 -0.118692
H -1.383688 -1.477003 0.639647
H -1.089690 -1.940627 -1.056196
C 1.687101 -1.252486 -0.798715
C 2.735648 -0.735817 -0.075473
H 1.719103 -1.751098 -1.758631
H 3.785944 -0.739416 -0.333437
C 2.152008 -0.011174 1.010419
O 0.852211 -0.461362 1.157306
H 2.640810 0.266014 1.936496
N -1.679025 0.089901 -0.747517
C -0.951403 1.356729 -0.294482
H -2.654129 0.056035 -0.371161
H -1.732553 0.103650 -1.770047
H -1.057456 1.401108 0.791943
H -1.504362 2.188703 -0.745285
C 0.484174 1.268183 -0.704722
C 1.534361 1.715649 0.125561
H 0.658776 1.236549 -1.778609
H 1.279133 2.211579 1.059609
H 2.430789 2.105815 -0.345575
O -4.030329 -0.303300 0.638016
H -4.787556 -0.844701 0.365103
H -4.368389 0.276219 1.338546
148
1a+CB[7] RS
C -2.125721 5.332798 0.155204
N -2.433349 4.703952 -1.102493
H -2.778441 6.201818 0.317639
C -1.299946 4.371891 -1.824631
N -0.210243 4.914874 -1.153146
O -1.269558 3.769802 -2.882504
C 1.019038 5.155394 -1.873316
N 2.183080 4.504862 -1.334165
H 1.214782 6.237379 -1.906104
H 0.875868 4.775992 -2.886924
C 2.920450 4.962855 -0.181961

N 2.314510 4.698976 1.106833
H 3.154651 6.032132 -0.285210
C 1.301541 5.553396 1.692429
N -0.051825 5.297532 1.264592
H 1.536084 6.604186 1.469970
H 1.336131 5.387138 2.771203
C -0.917344 4.500011 2.001593
N -2.130358 4.463261 1.318602
O -0.671373 3.965536 3.064610
C -3.340121 4.062301 2.005845
N -4.035598 2.946601 1.409595
H -4.023564 4.924716 2.061276
H -3.049523 3.758023 3.014014
C -4.237279 1.768462 2.124564
N -5.199426 1.035950 1.436957
O -3.714512 1.461222 3.174292
C -5.670153 1.693202 0.247360
N -5.230633 1.128412 -1.013134
H -6.766805 1.771603 0.261769
C -5.862516 -0.003471 -1.643532
N -5.353140 -1.294527 -1.248274
H -5.694649 0.091174 -2.718054
H -6.939796 0.015275 -1.432567
C -5.664721 -1.964944 -0.006359
C -4.904997 -3.327382 -0.140101
H -6.752419 -2.074617 0.098263
H -5.562366 -4.184185 -0.341504
N -4.251374 -3.473755 1.132100
C -3.632334 -4.683243 1.626038
H -4.258449 -5.536056 1.334144
H -3.594421 -4.604672 2.714567
N -2.278283 -4.928785 1.185307
C -1.177698 -4.706336 2.011753
O -1.208106 -4.346709 3.166393
N -0.039076 -5.036443 1.270270
C 1.222463 -5.271622 1.947463
H 1.473036 -6.341599 1.892498
H 1.072999 -4.981017 2.989688
N 2.342048 -4.518584 1.443809
C 2.832770 -3.393444 2.101878
O 2.357558 -2.875376 3.089151
N 3.997478 -3.008940 1.441079
C 4.935660 -2.110219 2.077375
H 5.907950 -2.618658 2.171411
H 4.542028 -1.880702 3.070497
N 5.117722 -0.852090 1.396298
C 4.803895 0.345649 2.036543
O 4.192996 0.470088 3.075712
N 5.366063 1.369092 1.280646
C 5.311367 2.749261 1.706844
H 6.267040 3.223473 1.448339
H 5.176992 2.751078 2.790566
N 4.230990 3.536259 1.160855
C 3.145116 3.937054 1.933916
O 2.978227 3.712594 3.111820
C 4.184572 4.041596 -0.183874
N 3.885176 3.065440 -1.216347
H 5.114860 4.573520 -0.429661
C 2.711306 3.359821 -1.897989
O 2.256500 2.746462 -2.849793
C 4.903603 2.240488 -1.825546

N 5.155822 1.002646 -1.136269
H 5.835999 2.822656 -1.889699
H 4.564495 1.979226 -2.830241
C 4.986665 -0.210006 -1.786662
N 5.579153 -1.182332 -0.994331
O 4.461729 -0.380050 -2.870895
C 5.603870 -2.568103 -1.391242
N 4.501451 -3.383556 -0.933750
H 6.538385 -3.014443 -1.031918
H 5.577788 -2.590812 -2.482200
C 3.398749 -3.634667 -1.729411
N 2.536208 -4.443119 -0.992802
O 3.223194 -3.266294 -2.875654
C 1.506653 -5.185729 -1.671405
N 0.169845 -4.984964 -1.163717
H 1.722295 -6.262889 -1.617165
H 1.519603 -4.861574 -2.713691
C -0.745477 -4.160233 -1.773757
N -1.974878 -4.372585 -1.180546
O -0.517766 -3.398715 -2.711459
C -1.908712 -5.377159 -0.125318
C -0.374901 -5.645248 0.001401
H -2.490816 -6.262173 -0.416761
H -0.107765 -6.711036 -0.010772
C -3.201569 -4.078826 -1.892962
N -4.027148 -3.082426 -1.272278
H -3.771736 -5.013972 -2.001269
H -2.931146 -3.699305 -2.880219
C -4.305563 -1.889528 -1.923460
O -3.744361 -1.476845 -2.924043
C 3.111177 -4.854423 0.277649
C 4.371888 -3.936215 0.393578
H 3.346515 -5.928130 0.263029
H 5.288531 -4.477746 0.666046
C 6.019838 -0.661690 0.276484
C 5.966062 0.887019 0.065728
H 7.022959 -1.041945 0.514256
H 6.951389 1.348404 -0.087361
C -4.334061 -2.322997 1.911618
N -5.090017 -1.393345 1.199693
O -3.866056 -2.178407 3.020759
C -5.654475 -0.248267 1.895347
H -6.750102 -0.286266 1.812109
H -5.359259 -0.337174 2.943190
C -4.472584 2.018572 -1.765533
N -4.331097 3.171414 -1.011549
O -4.060282 1.837712 -2.895297
C -4.943578 3.079334 0.290691
H -5.625241 3.927675 0.448551
C -3.756297 4.371107 -1.564725
H -3.693194 4.223983 -2.644550
H -4.419783 5.216827 -1.341239
C -0.614268 5.699174 0.004120
H -0.434140 6.768121 -0.176203
C -1.068187 0.452977 -2.305530
C -1.259426 0.368023 -0.839370
H -0.705978 1.442775 -2.592549
H -1.995982 0.236908 -2.842098
C -1.805916 -0.578730 -0.015939
C -1.601003 -0.128336 1.324584
H -2.267380 -1.504777 -0.324799

H -1.922922 -0.610931 2.236785
C -0.949566 1.066217 1.225021
O -0.735498 1.386183 -0.086780
H -0.621978 1.790346 1.955832
N -0.044586 -0.569494 -2.808794
C 1.370579 -0.483460 -2.239786
H 0.022614 -0.457606 -3.825730
H -0.402983 -1.533085 -2.668421
H 1.699507 0.550044 -2.365733
H 1.980900 -1.128279 -2.879947
C 1.446271 -0.949297 -0.815782
C 1.975309 -0.230139 0.176239
H 1.101592 -1.964735 -0.625535
H 2.336581 0.782704 0.014395
H 2.065533 -0.624054 1.183868
148
1a+CB[7] TS
C 1.062742 -0.141984 -0.876230
C 1.552659 0.650980 0.193055
C 1.178544 -0.002951 1.346317
C 0.456737 -1.165858 0.931847
O 0.804436 -1.406950 -0.395710
H 1.968159 1.643544 0.091837
H 1.284679 0.347572 2.363649
C 1.191992 -0.051656 -2.353689
H 1.266377 0.989708 -2.673287
H 2.017483 -0.638086 -2.764834
N -0.100837 -0.633958 -2.876542
H -0.110998 -1.649737 -2.695567
C -1.333076 0.008884 -2.191519
H -1.562442 0.902146 -2.772933
H -2.136501 -0.716255 -2.322850
C -1.037733 0.381180 -0.758363
C -1.377574 -0.413253 0.363225
H -1.856337 -1.375671 0.186906
H -1.701991 0.069917 1.279548
H -0.964856 1.453125 -0.602051
H -0.166993 -0.531976 -3.893057
C -4.277919 4.078675 -0.018311
N -3.534783 4.191130 1.205001
C -2.720656 5.318457 1.595617
N -1.375243 5.339128 1.071628
C -0.266731 5.117900 1.882651
O -0.274704 4.925703 3.077989
N 0.861879 5.223042 1.065170
C 2.173418 5.380339 1.657130
N 3.151583 4.393734 1.268611
C 3.373830 3.240175 2.014895
O 2.821596 2.933746 3.049851
N 4.382497 2.526144 1.367176
C 5.088023 1.473398 2.062688
N 4.995746 0.176056 1.435958
C 4.399559 -0.888561 2.108794
O 3.723328 -0.821194 3.113040
N 4.778617 -2.050806 1.446914
C 4.381974 -3.354899 1.908367
N 3.255589 -3.946806 1.213661
C 2.120859 -4.328125 1.927714
O 1.835642 -4.004219 3.060078
N 1.396236 -5.195151 1.113960
C 0.225568 -5.891663 1.595238

N -1.050280 -5.361392 1.171945
C -1.800683 -4.517525 1.986325
O -1.526323 -4.180956 3.116693
N -2.952419 -4.176981 1.271753
C -4.108087 -3.657517 1.979552
N -4.607287 -2.394546 1.503964
C -4.268300 -1.191043 2.114560
O -3.537835 -1.054032 3.072483
N -4.966005 -0.184164 1.449651
C -5.148394 1.104283 2.081276
N -4.566394 2.209748 1.358971
H -4.652813 1.064004 3.054267
H -6.226434 1.283714 2.222094
C -5.877845 -0.714823 0.453506
N -5.707629 -0.229763 -0.894265
C -6.094171 1.078088 -1.367876
N -5.219429 2.167839 -1.009774
C -4.142991 2.537021 -1.804442
O -3.822433 2.042277 -2.868665
H -7.097645 1.305931 -0.990617
H -6.111897 1.021637 -2.457727
C -4.999321 -1.109847 -1.695309
O -4.681914 -0.944462 -2.856743
N -4.758861 -2.252957 -0.936249
C -4.407978 -3.479853 -1.607629
N -3.180911 -4.091927 -1.163169
C -1.968378 -3.882107 -1.787155
O -1.752022 -3.128315 -2.728497
N -1.044573 -4.721203 -1.192472
C 0.155234 -5.102052 -1.899997
N 1.375732 -4.680191 -1.275781
C 2.210811 -3.759208 -1.878846
O 1.934563 -3.066623 -2.848145
N 3.426679 -3.829542 -1.228834
C 4.592655 -3.117670 -1.680099
N 4.855600 -1.874445 -0.996700
C 4.943615 -0.697749 -1.730051
O 4.552007 -0.531610 -2.869762
N 5.610211 0.226596 -0.941332
C 5.898357 1.558305 -1.411867
N 4.962386 2.585551 -1.019747
C 3.854515 2.890507 -1.794031
O 3.559033 2.398076 -2.865916
N 3.158034 3.900487 -1.134107
C 2.239467 4.721289 -1.890946
N 0.913022 4.806747 -1.345360
C -0.151092 4.091231 -1.863963
O -0.093697 3.233445 -2.731338
N -1.303824 4.564990 -1.252200
C -2.587915 4.426046 -1.900301
H -3.008846 5.432019 -2.057744
H -2.429607 3.942999 -2.866579
H 2.641449 5.741981 -1.975744
H 2.162357 4.273883 -2.883769
H 6.894172 1.849241 -1.058202
H 5.889641 1.516355 -2.502485
H 5.459530 -3.786032 -1.574730
H 4.443196 -2.874008 -2.733711
H 0.166466 -6.197430 -2.007811
H 0.117765 -4.639829 -2.887933
H -4.294012 -3.236757 -2.665799

H -5.212054 -4.219334 -1.483484
H -6.920407 -0.581845 0.774385
C -5.440095 -2.211711 0.348487
H -6.280980 -2.919492 0.360318
H -4.925748 -4.391271 1.932094
H -3.798183 -3.516064 3.017200
C -3.051767 -4.906315 0.021975
H -3.860369 -5.648697 0.061708
C -1.628127 -5.529494 -0.131691
H -1.635130 -6.588809 -0.422275
H 0.243796 -5.830420 2.685294
H 0.287422 -6.940458 1.278307
C 2.003112 -5.385955 -0.175417
H 2.088583 -6.456451 -0.408665
C 3.379187 -4.654487 -0.044724
H 4.242289 -5.334267 -0.025147
H 5.248367 -4.027763 1.831029
H 4.085473 -3.252149 2.954472
C 5.562250 -1.792054 0.270839
H 6.454156 -2.434391 0.252436
C 5.889607 -0.267259 0.384418
H 6.934874 -0.058896 0.652515
H 6.149061 1.752160 2.161407
H 4.636688 1.387179 3.053942
C 4.945395 3.266156 0.250912
H 5.948069 3.642686 0.494986
C 3.884313 4.388666 0.033489
H 4.318582 5.377671 -0.166867
H 2.037653 5.298554 2.737607
H 2.576305 6.372198 1.407341
C 0.532408 5.684862 -0.266285
H 0.937741 6.692115 -0.439289
C -1.029538 5.622929 -0.294424
H -1.500473 6.559054 -0.626720
H -2.625155 5.295165 2.683174
H -3.241353 6.234180 1.286904
C -3.640573 3.044559 1.987848
O -3.071486 2.834821 3.036649
N -3.530178 3.613756 -1.178797
H -4.801393 5.018227 -0.243082
C -5.227701 2.872261 0.246935
H -6.253039 3.162728 0.514537
H 0.276552 -2.052518 1.528766
151
1a+CB[7]+wat RS
C -1.652727 -5.327161 0.170379
N -1.676713 -4.311801 1.212238
H -2.247252 -6.198360 0.479703
C -0.436719 -4.130512 1.801857
N 0.453502 -4.974421 1.162283
O -0.181307 -3.406087 2.754108
C 1.801413 -5.180277 1.622881
N 2.807900 -4.390135 0.951934
H 2.037891 -6.247628 1.509188
H 1.841973 -4.910224 2.679593
C 3.369412 -4.733033 -0.346947
N 2.540646 -4.444058 -1.483142
H 3.693190 -5.782585 -0.359065
C 1.442733 -5.255422 -1.949528
N 0.187265 -5.042367 -1.267505
H 1.741545 -6.309749 -1.861061

H 1.270976 -5.007797 -2.999183
C -0.957532 -4.705374 -1.989819
N -2.041897 -4.892213 -1.144984
O -0.995640 -4.349488 -3.148993
C -3.403982 -4.741393 -1.610773
N -4.107869 -3.576226 -1.141430
H -3.972428 -5.634214 -1.317523
H -3.356114 -4.672058 -2.699484
C -4.319518 -2.453944 -1.939551
N -5.103133 -1.572935 -1.195752
O -3.939976 -2.301243 -3.080277
C -5.585761 -2.177691 0.033416
N -5.328600 -1.457580 1.251334
H -6.655676 -2.415973 -0.046112
C -6.053034 -0.292290 1.693091
N -5.587027 0.965394 1.162046
H -5.945483 -0.231299 2.777657
H -7.108930 -0.422989 1.430229
C -5.899599 1.502375 -0.141814
C -5.203212 2.908549 -0.122696
H -6.989435 1.554910 -0.272387
H -5.913367 3.747603 -0.098229
N -4.443810 2.925579 -1.347915
C -3.741691 4.069818 -1.878217
H -4.399321 4.948933 -1.808440
H -3.525332 3.852527 -2.926675
N -2.472371 4.375673 -1.239320
C -1.301002 4.454154 -2.003499
O -1.137298 4.014782 -3.119370
N -0.377204 5.186332 -1.259824
C 0.955663 5.468648 -1.727152
H 1.158412 6.539149 -1.575233
H 0.973594 5.236943 -2.794386
N 2.021402 4.693275 -1.107290
C 2.883230 3.964787 -1.937009
O 2.665464 3.639468 -3.083114
N 4.054276 3.738432 -1.223992
C 5.170439 3.024932 -1.801130
H 6.096969 3.553780 -1.546274
H 5.021688 3.035163 -2.882703
N 5.311517 1.642995 -1.406693
C 4.747268 0.613152 -2.153305
O 4.135864 0.727164 -3.192585
N 5.068794 -0.577901 -1.504562
C 4.888992 -1.839654 -2.177017
H 5.869553 -2.313307 -2.344560
H 4.418426 -1.619468 -3.138247
N 4.033385 -2.772242 -1.474024
C 2.867520 -3.236940 -2.087081
O 2.275941 -2.708150 -3.004610
C 4.540472 -3.707176 -0.487886
N 4.685755 -3.189006 0.852464
H 5.483980 -4.157172 -0.825458
C 3.625486 -3.535587 1.672019
O 3.465380 -3.193197 2.831594
C 5.719932 -2.281339 1.287571
N 5.581509 -0.905605 0.868460
H 6.685958 -2.651872 0.925347
H 5.706560 -2.292115 2.378827
C 4.981907 0.054440 1.665432
N 5.118256 1.272835 1.015700

O 4.458237 -0.129186 2.752042
C 4.913690 2.515264 1.721100
N 3.856124 3.341035 1.189428
H 5.849948 3.093313 1.714793
H 4.646170 2.260855 2.748934
C 2.696378 3.559973 1.906108
N 2.065893 4.655224 1.348082
O 2.310625 2.927463 2.877780
C 0.826284 5.148615 1.875905
N -0.357426 4.755833 1.137622
H 0.873387 6.246206 1.911014
H 0.733654 4.753097 2.888891
C -1.372840 4.062884 1.790943
N -2.566459 4.431690 1.204293
O -1.225262 3.302141 2.735519
C -2.378966 5.162769 -0.024362
C -0.869522 5.555882 0.035031
H -3.061391 6.022793 -0.062114
H -0.700152 6.623665 0.231159
C -3.849299 4.035015 1.724102
N -4.426874 2.871716 1.101910
H -4.531315 4.890699 1.620420
H -3.717199 3.794345 2.780806
C -4.715851 1.759010 1.886801
O -4.309134 1.556112 3.012862
C 2.683534 5.099257 0.121768
C 4.026634 4.291525 0.101168
H 2.826875 6.188375 0.140938
H 4.916360 4.910602 0.280834
C 5.922067 1.169787 -0.195568
C 5.994942 -0.374989 -0.408502
H 6.901187 1.644385 -0.044204
H 6.998528 -0.741694 -0.664678
C -4.518017 1.723625 -2.043926
N -5.290328 0.852599 -1.284907
O -4.022533 1.493858 -3.128333
C -5.718084 -0.423493 -1.818711
H -6.812097 -0.497441 -1.725973
H -5.436227 -0.444376 -2.874098
C -4.189799 -1.906628 1.903634
N -3.746303 -3.037160 1.223534
O -3.698989 -1.438242 2.912151
C -4.671704 -3.433874 0.170453
H -5.215586 -4.344685 0.459825
C -2.891649 -3.969706 1.914872
H -2.607731 -3.504362 2.860723
H -3.451311 -4.898198 2.117552
C -0.128522 -5.628942 0.017593
H 0.115843 -6.700581 0.029638
C -0.952900 -0.110619 2.054856
C -1.262175 -0.308125 0.623103
H -0.882873 -1.073698 2.563445
H -1.723233 0.491086 2.540030
C -2.025709 0.371334 -0.280637
C -1.852331 -0.280215 -1.543322
H -2.608874 1.255321 -0.067927
H -2.308113 -0.019447 -2.486425
C -0.993642 -1.313879 -1.320587
O -0.631067 -1.359199 -0.002131
H -0.558882 -2.062107 -1.966068
N 0.389337 0.590337 2.301276

C 1.461008 0.438395 1.244742
H 0.812558 0.124334 3.139193
H 0.237604 1.588013 2.505205
H 1.537551 -0.627418 1.024287
H 2.370671 0.748313 1.760831
C 1.228022 1.252369 0.006789
C 1.485363 0.815316 -1.227689
H 0.892554 2.278509 0.157458
H 1.818357 -0.200480 -1.426216
H 1.380001 1.459485 -2.094835
O 1.657898 -1.227724 3.849190
H 1.314980 -2.102980 3.594125
H 2.609330 -1.274681 3.648095
151
1a+CB[7]+wat TS
C -0.136784 -5.359767 0.095307
N 0.159183 -4.577713 -1.091967
H 0.200319 -6.397297 -0.040467
C -0.980375 -4.119140 -1.727087
N -2.072715 -4.542273 -0.987019
O -1.018060 -3.500954 -2.781286
C -3.420276 -4.537149 -1.507598
N -4.291952 -3.514106 -0.986661
H -3.868911 -5.518106 -1.303961
H -3.360820 -4.378261 -2.585795
C -4.847368 -3.485150 0.348342
N -3.915570 -3.176593 1.414284
H -5.379337 -4.423323 0.557659
C -3.099791 -4.176861 2.071617
N -1.832684 -4.432954 1.436959
H -3.671035 -5.116602 2.133392
H -2.881015 -3.811440 3.077684
C -0.630111 -4.241802 2.107120
N 0.365179 -4.822836 1.332441
O -0.483375 -3.703992 3.186302
C 1.698272 -5.045364 1.845290
N 2.743583 -4.273292 1.225093
H 1.936944 -6.113764 1.738573
H 1.683998 -4.772557 2.902721
C 3.450629 -3.297223 1.920111
N 4.509188 -2.898257 1.103774
O 3.219869 -2.909394 3.045065
C 4.601780 -3.695876 -0.103439
N 4.505650 -2.984534 -1.352762
H 5.515526 -4.306162 -0.087361
C 5.491921 -2.055656 -1.837140
N 5.468147 -0.752879 -1.209289
H 5.293792 -1.907397 -2.900500
H 6.486182 -2.499831 -1.701234
C 6.260391 -0.380725 -0.051949
C 6.105151 1.181479 0.001514
H 7.299232 -0.711580 -0.185951
H 7.063873 1.716662 0.039552
N 5.357084 1.398500 1.225956
C 5.142542 2.690551 1.842559
H 6.037999 3.312523 1.707174
H 4.974446 2.508068 2.906240
N 4.000080 3.428926 1.354346
C 2.785043 3.459775 2.030490
O 2.529422 2.911135 3.080555
N 1.927255 4.285412 1.296899

C 0.767467 4.874855 1.927201
H 0.919367 5.963965 2.012721
H 0.683879 4.438630 2.925179
N -0.490033 4.622728 1.250392
C -1.582782 4.162614 1.995429
O -1.540585 3.653895 3.093578
N -2.729632 4.452726 1.261964
C -4.059682 4.197828 1.766946
H -4.689556 5.064735 1.529244
H -3.974724 4.085383 2.849809
N -4.706058 3.004644 1.276423
C -4.795038 1.855535 2.057269
O -4.389873 1.723228 3.189458
N -5.497466 0.909505 1.306749
C -6.014765 -0.279318 1.950370
H -7.100002 -0.341752 1.795606
H -5.797507 -0.181474 3.016191
N -5.420943 -1.516653 1.506152
C -4.279339 -2.025094 2.115234
O -3.731739 -1.580967 3.100418
C -5.784021 -2.228880 0.312496
N -5.431185 -1.596226 -0.950233
H -6.853953 -2.476067 0.320918
C -4.600693 -2.387805 -1.724622
O -4.246630 -2.162770 -2.870107
C -6.157698 -0.492904 -1.538274
N -5.703575 0.818278 -1.142501
H -7.220621 -0.578496 -1.281508
H -6.033805 -0.567228 -2.620213
C -4.667951 1.451725 -1.805035
N -4.408264 2.638221 -1.132044
O -4.104660 1.061175 -2.814033
C -3.711467 3.714334 -1.794714
N -2.482031 4.117858 -1.152117
H -4.379923 4.586702 -1.865202
H -3.454711 3.368088 -2.798113
C -1.280660 4.078109 -1.837856
N -0.396909 4.906341 -1.177511
O -1.053819 3.446678 -2.861265
C 0.897384 5.243296 -1.715509
N 2.019391 4.567836 -1.122307
H 1.039011 6.326834 -1.604899
H 0.886026 4.978342 -2.774323
C 2.749970 3.615284 -1.816753
N 3.909531 3.382399 -1.089793
O 2.442507 3.110780 -2.881173
C 3.979318 4.174132 0.124390
C 2.590432 4.892157 0.160731
H 4.832799 4.864443 0.077148
H 2.660207 5.982062 0.285844
C 5.074277 2.786915 -1.711083
N 5.417051 1.477557 -1.231274
H 5.933579 3.454631 -1.563556
H 4.858347 2.689065 -2.776811
C 4.991689 0.343890 -1.908141
O 4.360685 0.326467 -2.947035
C -0.917088 5.373883 0.083121
C -2.437007 5.031402 -0.016209
H -0.711839 6.446404 0.206888
H -3.077526 5.903683 -0.206286
C -5.318171 2.851390 -0.014627

C -6.054911 1.476253 0.094924
H -5.989292 3.694622 -0.229449
H -7.146749 1.566252 0.174263
C 5.231676 0.234718 1.982334
N 5.763745 -0.804160 1.234181
O 4.779808 0.156029 3.105756
C 5.632331 -2.174510 1.667175
H 6.560141 -2.709848 1.425310
H 5.491151 -2.147488 2.749759
C 3.259824 -3.098651 -1.948075
N 2.523378 -4.003633 -1.193378
O 2.895060 -2.556042 -2.974025
C 3.284759 -4.540308 -0.079325
H 3.452665 -5.618612 -0.216344
C 1.413736 -4.693665 -1.800012
H 1.268998 -4.263906 -2.792978
H 1.661777 -5.763901 -1.894976
C -1.686834 -5.213462 0.232041
H -2.218167 -6.171873 0.318983
C 1.026566 -0.107335 -2.076084
C 1.201917 -0.149824 -0.593308
H 0.943701 -1.114032 -2.490044
H 1.853479 0.408235 -2.567137
C 2.232300 0.359331 0.245525
C 1.963684 -0.110828 1.510798
H 2.954484 1.108027 -0.045929
H 2.440303 0.165744 2.440301
C 0.774854 -0.893736 1.394855
O 0.621665 -1.223957 0.054222
H 0.428853 -1.643085 2.096829
N -0.267975 0.629885 -2.397851
C -1.184125 0.697011 -1.194840
H -0.811733 0.120024 -3.146904
H -0.076329 1.589695 -2.714947
H -1.601848 -0.305542 -1.073700
H -1.987214 1.374253 -1.485485
C -0.413964 1.115885 0.017053
C -0.666466 0.568486 1.296039
H 0.034683 2.106478 -0.032928
H -1.501967 -0.118992 1.419294
H -0.484627 1.187181 2.169622
O -1.897625 -0.938284 -3.859853
H -1.788963 -1.871597 -3.595921
H -2.828255 -0.736517 -3.656544
151
1a+CB[7]+wat PS
C 0.701527 -5.333465 -0.179714
N 0.361501 -4.668278 1.065454
H 0.451807 -6.402451 -0.116809
C 1.470242 -4.179080 1.726908
N 2.579924 -4.426663 0.934480
O 1.482826 -3.675148 2.840794
C 3.930741 -4.312221 1.433071
N 4.681985 -3.174554 0.958390
H 4.477831 -5.225180 1.165569
H 3.874513 -4.221662 2.519368
C 5.226718 -3.025670 -0.372929
N 4.266076 -2.764339 -1.427672
H 5.854610 -3.891507 -0.623517
C 3.536342 -3.812431 -2.113381
N 2.302941 -4.198655 -1.480136

H 4.187315 -4.695779 -2.209382
H 3.279104 -3.434190 -3.105559
C 1.079848 -4.066091 -2.127826
N 0.140775 -4.754877 -1.372203
O 0.879903 -3.491890 -3.179081
C -1.179335 -5.044499 -1.882721
N -2.263661 -4.374656 -1.209613
H -1.340365 -6.132212 -1.828778
H -1.200301 -4.719123 -2.925106
C -3.131800 -3.532558 -1.898995
N -4.235309 -3.319882 -1.078961
O -2.975941 -3.108085 -3.024740
C -4.149677 -4.038707 0.170479
N -4.000506 -3.232970 1.357065
H -5.010038 -4.714495 0.278433
C -5.048482 -2.426406 1.918343
N -5.262385 -1.157952 1.258032
H -4.767950 -2.210352 2.951408
H -5.985766 -3.000137 1.898461
C -6.169090 -0.951927 0.142414
C -6.240140 0.612852 0.031975
H -7.140555 -1.419313 0.353775
H -7.265667 1.003937 -0.011351
N -5.542583 0.881008 -1.214024
C -5.440185 2.177651 -1.847801
H -6.381396 2.728293 -1.715308
H -5.264777 1.993294 -2.910022
N -4.358083 3.012615 -1.373695
C -3.141865 3.110100 -2.039996
O -2.835217 2.549765 -3.069532
N -2.350756 4.018137 -1.324580
C -1.234697 4.667194 -1.973587
H -1.471491 5.734601 -2.120795
H -1.099593 4.187484 -2.945559
N 0.030256 4.555330 -1.267779
C 1.177183 4.199758 -1.991898
O 1.205318 3.651380 -3.071135
N 2.276933 4.638571 -1.258620
C 3.635468 4.521775 -1.739443
H 4.163395 5.452960 -1.496390
H 3.584892 4.395155 -2.822915
N 4.392367 3.404317 -1.230047
C 4.673029 2.297462 -2.027438
O 4.349129 2.145312 -3.183077
N 5.448594 1.430313 -1.256254
C 6.119229 0.309699 -1.880342
H 7.195644 0.357478 -1.671026
H 5.946569 0.399486 -2.954885
N 5.636537 -0.988438 -1.475177
C 4.543638 -1.575514 -2.105576
O 3.972185 -1.156298 -3.087812
C 6.022861 -1.681076 -0.278750
N 5.547979 -1.117167 0.978909
H 7.112215 -1.814283 -0.242792
C 4.828727 -2.035371 1.725301
O 4.440477 -1.888507 2.873019
C 6.114010 0.056282 1.606244
N 5.524454 1.309213 1.198762
H 7.189963 0.101831 1.396717
H 5.953519 -0.046768 2.681013
C 4.404268 1.819181 1.827863

N 4.049819 2.988911 1.163005
O 3.840423 1.350722 2.802147
C 3.235161 3.981734 1.821924
N 1.990562 4.280257 1.146988
H 3.816432 4.911189 1.928522
H 2.981436 3.595051 2.811189
C 0.785151 4.186098 1.824666
N -0.139208 4.946757 1.141701
O 0.587868 3.561393 2.859496
C -1.464865 5.215954 1.648472
N -2.524213 4.433745 1.074449
H -1.673469 6.281843 1.484828
H -1.454504 5.001349 2.718744
C -3.231990 3.508186 1.830441
N -4.330005 3.123007 1.074957
O -2.953018 3.140916 2.955183
C -4.419954 3.827301 -0.189645
C -3.083313 4.638454 -0.238198
H -5.319395 4.458553 -0.206315
H -3.228466 5.711318 -0.431021
C -5.438816 2.412951 1.674948
N -5.590890 1.049914 1.242337
H -6.370135 2.953379 1.462252
H -5.257921 2.393552 2.751436
C -4.947755 0.019654 1.914813
O -4.271954 0.133660 2.918881
C 0.364842 5.398895 -0.130684
C 1.902814 5.191228 0.008251
H 0.071970 6.442449 -0.310379
H 2.460584 6.114700 0.215285
C 4.971698 3.311435 0.082243
C 5.866366 2.030275 -0.005663
H 5.533859 4.223817 0.325685
H 6.943395 2.245533 -0.019518
C -5.320969 -0.278424 -1.953026
N -5.695433 -1.352430 -1.159701
O -4.913808 -0.334058 -3.095210
C -5.448061 -2.711682 -1.583525
H -6.302837 -3.332116 -1.284863
H -5.369017 -2.687554 -2.672345
C -2.758558 -3.354649 1.955262
N -2.042373 -4.288839 1.221673
O -2.388812 -2.802145 2.975093
C -2.773772 -4.782507 0.076165
H -2.862416 -5.877872 0.125902
C -0.876197 -4.921277 1.773148
H -0.748189 -4.540240 2.788142
H -1.042489 -6.010047 1.804823
C 2.234010 -5.053440 -0.320236
H 2.838816 -5.959891 -0.466896
C -1.013984 0.083471 2.058644
C -1.072658 -0.003606 0.545545
H -1.094994 -0.899072 2.525534
H -1.782042 0.738940 2.473383
C -2.319567 0.243514 -0.276887
C -2.062039 -0.302502 -1.471903
H -3.143152 0.878231 0.017584
H -2.633371 -0.240688 -2.388028
C -0.671974 -0.907035 -1.359001
O -0.616933 -1.278300 0.042792
H -0.436992 -1.750036 -2.005256

N 0.358039 0.677684 2.357945
C 1.132400 0.757945 1.060850
H 0.914922 0.071463 3.026877
H 0.271516 1.623469 2.755338
H 1.656209 -0.194181 0.956861
H 1.854864 1.565834 1.134437
C 0.069361 0.929947 -0.013371
C 0.353692 0.284069 -1.390868
H -0.255589 1.973198 -0.081540
H 1.374672 -0.104083 -1.467342
H 0.189060 0.967590 -2.226732
O 1.925597 -0.989751 3.753412
H 1.821045 -1.936289 3.533151
H 2.867499 -0.806288 3.586653
154
1b+CB[7]+wat RS
C -1.950487 -5.375799 0.004516
N -2.192884 -4.621735 1.211144
H -2.581209 -6.276041 -0.006130
C -1.033801 -4.272546 1.865751
N 0.024588 -4.855061 1.192865
O -0.962593 -3.610175 2.893196
C 1.309385 -5.016640 1.828811
N 2.391922 -4.325298 1.173130
H 1.548047 -6.090408 1.879519
H 1.231059 -4.617300 2.841305
C 3.101314 -4.872654 0.024448
N 2.441519 -4.739902 -1.243120
H 3.373783 -5.920275 0.213659
C 1.383134 -5.597680 -1.726491
N 0.062101 -5.312043 -1.218664
H 1.641827 -6.636246 -1.481199
H 1.337289 -5.471743 -2.810309
C -0.920478 -4.728208 -2.012585
N -2.079883 -4.649042 -1.242570
O -0.799024 -4.398299 -3.172816
C -3.357080 -4.389462 -1.882249
N -4.077509 -3.249399 -1.378438
H -3.999420 -5.276811 -1.783124
H -3.146304 -4.203854 -2.937606
C -4.100826 -2.028761 -2.046120
N -4.947085 -1.183549 -1.328849
O -3.535359 -1.771779 -3.087836
C -5.616287 -1.874778 -0.243081
N -5.423128 -1.329304 1.076466
H -6.689764 -1.975438 -0.457070
C -6.039081 -0.125443 1.572217
N -5.451104 1.114276 1.122011
H -5.948462 -0.149754 2.659794
H -7.097639 -0.127071 1.285938
C -5.748367 1.781196 -0.121126
C -5.052797 3.174557 0.045664
H -6.836213 1.846460 -0.262402
H -5.755644 3.998657 0.231676
N -4.379231 3.359090 -1.211393
C -3.683608 4.552451 -1.623023
H -4.298901 5.424207 -1.361391
H -3.563008 4.494415 -2.706930
N -2.357290 4.725108 -1.062766
C -1.224854 4.722953 -1.879619
O -1.179435 4.391928 -3.044109

N -0.173217 5.224139 -1.118696
C 1.116979 5.523642 -1.696832
H 1.346465 6.584913 -1.524368
H 1.029524 5.332494 -2.768631
N 2.223130 4.734607 -1.207940
C 2.810034 3.739399 -1.983966
O 2.407315 3.331360 -3.052395
N 3.969083 3.335345 -1.326708
C 4.959517 2.535574 -2.014671
H 5.922659 3.066596 -1.997209
H 4.617636 2.421918 -3.045992
N 5.147642 1.208683 -1.483816
C 4.711170 0.084991 -2.173744
O 4.048368 0.071211 -3.189066
N 5.222427 -1.023542 -1.507082
C 5.021375 -2.359398 -1.998008
H 5.980123 -2.897718 -1.963864
H 4.682673 -2.272626 -3.032842
N 4.016061 -3.137608 -1.289869
C 2.957263 -3.700580 -2.010863
O 2.585350 -3.375304 -3.117291
C 4.328833 -3.915758 -0.104369
N 4.314082 -3.195630 1.146260
H 5.286230 -4.440839 -0.226138
C 3.137832 -3.371638 1.844187
O 2.833126 -2.819808 2.892056
C 5.335835 -2.269687 1.574648
N 5.300897 -0.975232 0.939644
H 6.313013 -2.737928 1.394626
H 5.194692 -2.108010 2.645027
C 5.049500 0.165076 1.696850
N 5.465586 1.254101 0.949837
O 4.598607 0.188062 2.825445
C 5.457615 2.589496 1.496454
N 4.353370 3.429571 1.098817
H 6.391383 3.090846 1.213650
H 5.405117 2.488064 2.582150
C 3.204083 3.529167 1.862948
N 2.357582 4.418039 1.212882
O 2.982726 2.980013 2.926086
C 1.231672 4.984620 1.903868
N -0.048564 4.751611 1.271335
H 1.371139 6.072008 2.007303
H 1.204122 4.524084 2.893498
C -1.043220 4.031103 1.906781
N -2.251200 4.410441 1.359171
O -0.884275 3.229139 2.819601
C -2.105531 5.307830 0.233787
C -0.567684 5.598961 0.212345
H -2.724800 6.202813 0.385011
H -0.314540 6.649056 0.415349
C -3.522450 4.018951 1.918964
N -4.204622 2.965115 1.210121
H -4.163848 4.911008 1.959118
H -3.336286 3.652392 2.930419
C -4.491888 1.773638 1.867538
O -4.016162 1.409205 2.926656
C 2.945831 4.983047 0.012272
C 4.254536 4.138175 -0.155108
H 3.128197 6.059198 0.141819
H 5.155351 4.746753 -0.314915

C 5.982916 0.890328 -0.349239
C 5.958558 -0.678270 -0.318465
H 6.988691 1.312341 -0.483969
H 6.959114 -1.132867 -0.339269
C -4.394181 2.213781 -2.004061
N -5.108989 1.246506 -1.308303
O -3.910914 2.105688 -3.110934
C -5.486306 0.008567 -1.949097
H -6.584576 -0.068888 -1.975041
H -5.096219 0.051197 -2.968753
C -4.493732 -2.035445 1.825027
N -4.116793 -3.141213 1.067737
O -4.114771 -1.772977 2.948797
C -4.862005 -3.242488 -0.174353
H -5.532478 -4.113459 -0.146572
C -3.500051 -4.272702 1.712700
H -3.388332 -4.018191 2.768517
H -4.147832 -5.155300 1.610052
C -0.419181 -5.684647 0.081792
H -0.192380 -6.739709 0.291635
C -1.181819 -0.227320 1.885894
C -1.280650 0.065941 0.440586
H -1.090201 -1.300676 2.059100
H -2.048292 0.141773 2.440321
C -1.668095 1.166908 -0.272153
C -1.348664 0.908946 -1.637714
H -2.109577 2.062714 0.136436
H -1.516783 1.560155 -2.483228
C -0.795512 -0.342270 -1.683583
O -0.765195 -0.876193 -0.418470
N 0.036377 0.407625 2.561137
C 1.371396 0.256732 1.866074
H 0.152716 -0.112743 3.463210
H -0.158945 1.406054 2.738079
H 1.522491 -0.813907 1.714033
H 2.113130 0.606023 2.592472
C 1.484506 1.025767 0.585346
C 1.960736 0.503700 -0.545800
H 1.200655 2.075110 0.622921
H 2.240546 -0.545592 -0.611820
H 2.075781 1.086950 -1.452553
O 0.590528 -1.629121 4.278235
H -0.012632 -2.342227 3.997824
H 1.465353 -1.944588 3.984761
C -0.297094 -1.183475 -2.801895
H -1.017965 -1.959920 -3.073118
H -0.140285 -0.552010 -3.680190
H 0.653580 -1.668614 -2.559708
154
1b+CB[7]+wat TS
C -0.909605 -5.630978 -0.062292
N -0.293567 -5.019284 -1.213720
H -0.758458 -6.719522 -0.091314
C -1.120671 -4.117180 -1.843421
N -2.355410 -4.169167 -1.218549
O -0.828758 -3.443802 -2.822944
C -3.539808 -3.684886 -1.884129
N -4.210528 -2.611359 -1.194745
H -4.241969 -4.523159 -2.015591
H -3.243881 -3.309656 -2.864900
C -5.146451 -2.832244 -0.096977

N -4.567054 -3.137067 1.179586
H -5.887534 -3.592528 -0.379852
C -4.071733 -4.432110 1.589376
N -2.742881 -4.768798 1.135104
H -4.773421 -5.198186 1.235934
H -4.038747 -4.432767 2.680871
C -1.638059 -4.714612 1.981987
N -0.527994 -5.094092 1.228839
O -1.651548 -4.431298 3.159909
C 0.714907 -5.433600 1.893658
N 1.879321 -4.737121 1.410728
H 0.899781 -6.514137 1.801154
H 0.583126 -5.172545 2.945842
C 2.413616 -3.633837 2.067291
N 3.600301 -3.302155 1.414195
O 1.958572 -3.098472 3.056850
C 3.944724 -4.264231 0.383479
N 4.130060 -3.734483 -0.943226
H 4.823090 -4.853028 0.683429
C 5.295301 -3.019498 -1.397217
N 5.387557 -1.635496 -0.993393
H 5.269626 -3.033663 -2.488349
H 6.190609 -3.540711 -1.038436
C 5.855109 -1.163086 0.285117
C 5.967966 0.379507 0.069977
H 6.808171 -1.645055 0.542862
H 6.994177 0.728397 -0.108757
N 5.454320 0.917554 1.297556
C 5.470213 2.308859 1.674192
H 6.450035 2.725131 1.403425
H 5.333720 2.356075 2.756584
N 4.421164 3.118143 1.091750
C 3.489853 3.764238 1.906861
O 3.328803 3.591784 3.093892
N 2.827104 4.689233 1.105857
C 1.840313 5.592609 1.647193
H 2.102003 6.622277 1.369724
H 1.882157 5.478977 2.732477
N 0.477327 5.352725 1.237153
C -0.405327 4.600357 2.006175
O -0.149416 4.037812 3.048791
N -1.649241 4.660200 1.376195
C -2.842847 4.329940 2.124568
H -3.452754 5.238268 2.253630
H -2.514374 3.965605 3.100709
N -3.661095 3.299023 1.535583
C -3.859126 2.083883 2.183343
O -3.240896 1.664182 3.139258
N -4.935826 1.462038 1.560764
C -5.491114 0.228333 2.051962
H -6.587978 0.310579 2.041087
H -5.141450 0.097264 3.078464
N -5.087189 -0.959447 1.322488
C -4.508449 -2.025736 2.017080
O -4.075370 -2.002856 3.147644
C -5.751391 -1.409376 0.110823
N -5.393437 -0.724024 -1.107065
H -6.842601 -1.406491 0.238416
C -4.414682 -1.391265 -1.813681
O -3.866963 -0.989867 -2.830555
C -5.845570 0.593957 -1.486407

N -5.137179 1.686060 -0.869123
H -6.914518 0.664177 -1.245530
H -5.702587 0.690465 -2.564323
C -4.385145 2.566567 -1.638709
N -4.110981 3.668536 -0.844320
O -4.070332 2.416302 -2.802984
C -3.473721 4.844719 -1.382500
N -2.094371 5.044180 -1.008518
H -4.041348 5.730756 -1.071234
H -3.502947 4.748754 -2.469546
C -1.043881 4.604458 -1.795619
N 0.129780 4.990572 -1.162146
O -1.124490 4.027305 -2.865554
C 1.378276 4.971737 -1.877295
N 2.414895 4.179218 -1.254198
H 1.753064 6.000446 -1.994765
H 1.173794 4.545580 -2.861398
C 2.930444 3.055277 -1.875958
N 4.169609 2.802293 -1.322067
O 2.402268 2.424515 -2.783155
C 4.493439 3.693940 -0.230761
C 3.312614 4.719274 -0.246178
H 5.477980 4.151531 -0.402196
H 3.616973 5.737248 -0.527840
C 5.084415 1.823165 -1.855559
N 5.145396 0.582892 -1.118773
H 6.083757 2.280353 -1.897311
H 4.753236 1.573722 -2.865604
C 4.895725 -0.613646 -1.785962
O 4.378389 -0.728412 -2.881045
C -0.119808 5.810913 0.015300
C -1.644912 5.580557 0.251957
H 0.147610 6.858566 -0.179819
H -2.199630 6.497721 0.493909
C -4.634741 3.547549 0.495969
C -5.451521 2.211299 0.444869
H -5.245399 4.427386 0.743925
H -6.536577 2.354048 0.547110
C 4.755616 -0.031863 2.038951
N 4.914520 -1.252425 1.390089
O 4.159098 0.169440 3.075821
C 4.589339 -2.481939 2.076371
H 5.508595 -3.072981 2.217186
H 4.180603 -2.201568 3.049895
C 3.013269 -3.903661 -1.750018
N 2.103087 -4.665977 -1.025117
O 2.873472 -3.508412 -2.889903
C 2.638721 -5.110267 0.249078
H 2.814368 -6.195419 0.235915
C 1.025207 -5.335138 -1.705594
H 1.072173 -5.027880 -2.752158
H 1.157544 -6.424647 -1.633296
C -2.405515 -5.202265 -0.190901
H -3.071111 -6.015383 -0.513763
C 1.061876 -0.451565 -2.194862
C 0.941173 -0.279739 -0.717604
H 0.765794 -1.460323 -2.491364
H 2.066330 -0.247392 -2.564574
C 1.878546 0.137270 0.272315
C 1.271157 -0.081167 1.483959
H 2.793793 0.676497 0.070837

H 1.626409 0.215578 2.461498
C -0.022653 -0.640234 1.202197
O 0.034750 -1.121437 -0.105546
N 0.104060 0.519989 -2.875919
C -1.013855 0.937782 -1.941040
H -0.344243 0.044277 -3.697143
H 0.636656 1.353870 -3.154033
H -1.663465 0.066612 -1.834329
H -1.563851 1.728498 -2.458820
C -0.421451 1.343985 -0.626616
C -1.045530 1.037097 0.604376
H 0.233538 2.212053 -0.665927
H -2.012122 0.536300 0.577531
H -0.930844 1.712662 1.446034
O -1.446887 -1.131333 -4.371937
H -1.221585 -2.000190 -3.988086
H -2.342222 -0.958567 -4.025501
C -0.913071 -1.405079 2.130496
H -0.370898 -2.199092 2.650537
H -1.355241 -0.740144 2.877845
H -1.731826 -1.849010 1.557843
154
1b+CB[7]+wat PS
C 1.531436 -5.476847 0.030069
N 0.842839 -4.954184 1.183579
H 1.497355 -6.575775 0.040423
C 1.557285 -3.969901 1.827227
N 2.793605 -3.873280 1.209058
O 1.186068 -3.350602 2.815148
C 3.910742 -3.265175 1.887005
N 4.473687 -2.126959 1.203027
H 4.697802 -4.022376 2.029945
H 3.564667 -2.922737 2.863124
C 5.446544 -2.247229 0.120735
N 4.928311 -2.615543 -1.165156
H 6.260018 -2.922337 0.420308
C 4.573325 -3.956187 -1.575673
N 3.280191 -4.425049 -1.138920
H 5.343916 -4.646038 -1.209039
H 4.557337 -3.961568 -2.667525
C 2.180599 -4.463528 -1.994757
N 1.108594 -4.961045 -1.256293
O 2.175468 -4.163103 -3.168231
C -0.093158 -5.405300 -1.932650
N -1.322054 -4.846745 -1.429758
H -0.167005 -6.501639 -1.873458
H 0.010256 -5.100439 -2.976220
C -1.980046 -3.802099 -2.068338
N -3.207525 -3.635913 -1.427515
O -1.583458 -3.190383 -3.038846
C -3.440888 -4.650351 -0.417198
N -3.689991 -4.169805 0.918100
H -4.245379 -5.329886 -0.732417
C -4.927982 -3.594396 1.375681
N -5.175578 -2.225449 0.983094
H -4.900888 -3.615404 2.466686
H -5.760261 -4.208147 1.011311
C -5.701781 -1.797821 -0.288536
C -5.992339 -0.281873 -0.056867
H -6.592923 -2.385416 -0.548340
H -7.049108 -0.057656 0.142126

N -5.566056 0.322789 -1.286707
C -5.754036 1.705399 -1.649614
H -6.770762 1.999471 -1.356378
H -5.645925 1.776706 -2.733847
N -4.798618 2.630653 -1.080875
C -3.943193 3.364178 -1.902677
O -3.784794 3.218282 -3.093468
N -3.356341 4.340794 -1.102293
C -2.481177 5.344482 -1.656184
H -2.845877 6.341788 -1.376934
H -2.523575 5.224751 -2.740781
N -1.094871 5.252975 -1.261673
C -0.154993 4.556795 -2.014900
O -0.363450 3.949639 -3.042483
N 1.081917 4.730899 -1.386969
C 2.295070 4.506114 -2.143375
H 2.797719 5.470832 -2.321213
H 1.997697 4.066035 -3.098077
N 3.228564 3.596737 -1.522825
C 3.616106 2.428853 -2.178309
O 3.060402 1.914979 -3.126146
N 4.786943 1.990546 -1.571001
C 5.499178 0.827700 -2.035751
H 6.576565 1.043361 -1.993814
H 5.198446 0.651162 -3.070845
N 5.216894 -0.395788 -1.310108
C 4.777049 -1.521855 -2.013629
O 4.381325 -1.550248 -3.157207
C 5.899035 -0.767635 -0.082480
N 5.443966 -0.121523 1.124657
H 6.986181 -0.647865 -0.186672
C 4.531485 -0.890420 1.816419
O 3.925455 -0.543617 2.821252
C 5.726927 1.243328 1.498117
N 4.891375 2.234623 0.865249
H 6.781038 1.444205 1.265206
H 5.563794 1.329660 2.574003
C 4.063985 3.048040 1.633004
N 3.678870 4.110382 0.829370
O 3.768950 2.879921 2.799759
C 2.905074 5.210404 1.349416
N 1.510645 5.237252 0.976172
H 3.362223 6.152017 1.020850
H 2.947004 5.137940 2.437830
C 0.515897 4.703569 1.778059
N -0.694467 4.936775 1.138288
O 0.663401 4.161957 2.859876
C -1.932092 4.805564 1.865176
N -2.887955 3.907170 1.261855
H -2.404737 5.794188 1.971760
H -1.679151 4.415664 2.852996
C -3.255292 2.723616 1.878431
N -4.452970 2.318718 1.323492
O -2.647345 2.162393 2.781047
C -4.900080 3.179748 0.249815
C -3.840758 4.331523 0.251208
H -5.924926 3.522695 0.450521
H -4.256892 5.312269 0.522188
C -5.244217 1.241187 1.865113
N -5.177497 0.005741 1.120690
H -6.288006 1.582795 1.925732

H -4.871169 1.025340 2.868310
C -4.791218 -1.160535 1.778117
O -4.251324 -1.223160 2.866433
C -0.536934 5.772462 -0.046918
C 0.999298 5.686892 -0.294260
H -0.902080 6.790324 0.146528
H 1.458361 6.645430 -0.573028
C 4.185666 4.011898 -0.517929
C 5.170019 2.797576 -0.444036
H 4.670054 4.953946 -0.811570
H 6.228756 3.082873 -0.517810
C -4.754708 -0.526384 -2.036186
N -4.761755 -1.762443 -1.397123
O -4.186454 -0.244267 -3.069932
C -4.284223 -2.936756 -2.090618
H -5.123232 -3.634660 -2.242958
H -3.907784 -2.599140 -3.058962
C -2.568274 -4.240046 1.733605
N -1.579661 -4.888092 1.003019
O -2.481325 -3.858702 2.883538
C -2.046603 -5.344842 -0.292181
H -2.095644 -6.442871 -0.318630
C -0.427824 -5.433796 1.670093
H -0.509796 -5.152847 2.721857
H -0.428181 -6.529855 1.578399
C 2.970635 -4.893104 0.181242
H 3.713702 -5.631592 0.514614
C -1.014202 -0.488255 2.224285
C -0.766319 -0.184207 0.757461
H -0.701492 -1.503982 2.473982
H -2.042933 -0.332336 2.544300
C -1.843649 -0.093746 -0.303441
C -1.195364 -0.235471 -1.465155
H -2.874731 0.178711 -0.120647
H -1.592012 -0.142620 -2.468802
C 0.279519 -0.449219 -1.112345
O 0.162873 -1.114869 0.178370
N -0.119619 0.492217 2.970197
C 0.888800 1.040773 1.981685
H 0.410452 0.001590 3.738339
H -0.698841 1.259699 3.328065
H 1.674541 0.287912 1.901669
H 1.303165 1.966503 2.380288
C 0.087606 1.146115 0.691528
C 0.844300 0.944437 -0.637363
H -0.546316 2.037793 0.703344
H 1.924256 0.850510 -0.477891
H 0.674822 1.734626 -1.371385
O 1.588910 -1.041747 4.391141
H 1.435363 -1.916533 3.983094
H 2.455608 -0.774618 4.030322
C 1.161820 -1.223626 -2.058638
H 0.646750 -2.093249 -2.470474
H 1.495528 -0.589446 -2.885263
H 2.052256 -1.557023 -1.516489
151
1c+CB[7]+wat RS
C -0.953603 -5.565042 0.172606
N -1.288599 -4.715734 1.291835
H -1.457535 -6.536486 0.281848
C -0.182184 -4.172294 1.902974

N 0.939801 -4.672455 1.270174
O -0.193594 -3.430023 2.878183
C 2.232081 -4.619818 1.909250
N 3.198469 -3.805866 1.220608
H 2.621862 -5.645257 2.001224
H 2.099642 -4.195095 2.905590
C 3.963352 -4.273894 0.071444
N 3.253959 -4.356863 -1.173183
H 4.449697 -5.230919 0.305792
C 2.384998 -5.444539 -1.571577
N 1.025508 -5.369477 -1.090060
H 2.838368 -6.384571 -1.231463
H 2.327568 -5.433397 -2.662115
C -0.037802 -5.057373 -1.934879
N -1.187072 -5.014329 -1.145985
O 0.025352 -4.899026 -3.134065
C -2.495851 -4.951059 -1.768781
N -3.354053 -3.893326 -1.291799
H -3.019568 -5.908317 -1.626116
H -2.321487 -4.782640 -2.833763
C -3.593032 -2.732439 -2.023357
N -4.579198 -2.014108 -1.340935
O -3.078977 -2.427551 -3.077220
C -5.149043 -2.775092 -0.246098
N -5.115306 -2.150648 1.052127
H -6.174492 -3.090016 -0.486601
C -5.994441 -1.098080 1.491158
N -5.653067 0.234609 1.048860
H -5.961593 -1.089095 2.582140
H -7.011471 -1.324238 1.150478
C -5.968316 0.798313 -0.240243
C -5.542134 2.296348 -0.084866
H -7.036466 0.664758 -0.459549
H -6.388117 2.984040 0.050925
N -4.856091 2.572356 -1.317497
C -4.378507 3.865363 -1.743308
H -5.156491 4.609567 -1.523479
H -4.212110 3.810637 -2.821286
N -3.124610 4.288136 -1.150938
C -2.018647 4.573201 -1.958505
O -1.898138 4.308319 -3.132995
N -1.109618 5.271300 -1.167944
C 0.121647 5.806933 -1.701357
H 0.172608 6.880053 -1.471477
H 0.080551 5.660537 -2.782899
N 1.336133 5.184720 -1.227147
C 2.050629 4.281765 -2.009367
O 1.711496 3.844653 -3.087550
N 3.246900 4.013950 -1.343495
C 4.351315 3.412077 -2.067023
H 5.206852 4.102573 -2.054577
H 4.012139 3.260771 -3.094216
N 4.788341 2.131469 -1.574527
C 4.425257 0.942912 -2.196484
O 3.735160 0.829780 -3.186396
N 5.042454 -0.091530 -1.494846
C 5.132246 -1.416400 -2.061227
H 6.193385 -1.702620 -2.136921
H 4.690811 -1.374845 -3.059855
N 4.410634 -2.438529 -1.327289
C 3.500949 -3.262738 -1.998361

O 3.046584 -3.085429 -3.107319
C 4.961494 -3.097156 -0.155235
N 4.884004 -2.358297 1.081517
H 5.995411 -3.421889 -0.337093
C 3.783939 -2.714962 1.835268
O 3.425566 -2.191357 2.880225
C 5.783938 -1.300880 1.479390
N 5.522079 -0.008390 0.904675
H 6.803910 -1.609800 1.217692
H 5.696651 -1.190683 2.561821
C 4.977993 1.023357 1.655128
N 5.014685 2.165208 0.867932
O 4.590294 0.947846 2.805620
C 4.834024 3.470725 1.458332
N 3.626203 4.163601 1.082933
H 5.688518 4.107302 1.193509
H 4.799087 3.329743 2.540349
C 2.466898 4.058782 1.828733
N 1.498011 4.816464 1.186538
O 2.321099 3.452201 2.875610
C 0.280997 5.170096 1.863794
N -0.928897 4.718526 1.207605
H 0.225462 6.264185 1.974673
H 0.320442 4.706510 2.851396
C -1.803240 3.852390 1.841604
N -3.050583 4.027603 1.279073
O -1.527780 3.094585 2.764957
C -3.035091 4.922657 0.144523
C -1.582792 5.499388 0.168107
H -3.816516 5.686830 0.259681
H -1.537444 6.567762 0.422351
C -4.250902 3.434720 1.816634
N -4.718174 2.262534 1.117161
H -5.035681 4.204651 1.817867
H -4.038110 3.125312 2.841817
C -4.863457 1.068347 1.818539
O -4.408791 0.828790 2.920943
C 2.005314 5.496651 0.006682
C 3.420207 4.850673 -0.170320
H 2.031742 6.582965 0.170323
H 4.222277 5.583580 -0.333360
C 5.631705 1.937515 -0.424920
C 5.895648 0.391801 -0.430511
H 6.546920 2.539591 -0.515296
H 6.944043 0.125873 -0.627015
C -4.590699 1.419325 -2.050180
N -5.156658 0.353088 -1.360870
O -4.001792 1.369497 -3.110516
C -5.289544 -0.940807 -1.998192
H -6.357797 -1.200499 -2.064400
H -4.872472 -0.844046 -3.003366
C -4.068564 -2.607466 1.839848
N -3.456482 -3.640460 1.140255
O -3.770981 -2.217527 2.951869
C -4.147669 -3.963857 -0.095129
H -4.637681 -4.944960 -0.014902
C -2.615382 -4.577177 1.837932
H -2.518604 -4.216262 2.863673
H -3.087278 -5.571318 1.838778
C 0.605668 -5.658772 0.251792
H 0.970366 -6.647410 0.566359

C -1.177999 -0.195210 2.285475
C -1.305471 -0.214288 0.815628
H -1.060532 -1.209685 2.668426
H -2.054839 0.260816 2.748238
C -1.958517 0.556539 -0.103253
C -1.565505 0.099761 -1.399085
H -2.619504 1.380068 0.121298
H -1.886338 0.466942 -2.361920
C -0.687059 -0.917951 -1.169543
O -0.532577 -1.153331 0.161066
N 0.041560 0.593356 2.779321
C 1.295249 0.564170 1.931019
H 0.332212 0.129536 3.669733
H -0.244718 1.570942 2.938847
H 1.533545 -0.486223 1.756966
H 2.073738 0.994853 2.569077
C 1.174675 1.336244 0.651477
C 1.679748 0.917167 -0.509227
H 0.695196 2.310505 0.715136
H 2.159180 -0.054069 -0.605308
H 1.631658 1.515052 -1.413317
O 1.109603 -1.337042 4.374645
H 0.666216 -2.135788 4.032198
H 2.026432 -1.444575 4.061255
Cl 0.238278 -1.884367 -2.226012
151
1c+CB[7]+wat TS
C -1.296290 -5.526518 -0.159040
N -0.637644 -4.917275 -1.288926
H -1.206162 -6.620621 -0.221023
C -1.407585 -3.953260 -1.897828
N -2.647279 -3.952646 -1.284151
O -1.068954 -3.269971 -2.856772
C -3.795955 -3.382750 -1.944518
N -4.396526 -2.277990 -1.241955
H -4.550407 -4.172151 -2.087456
H -3.475733 -3.014195 -2.920170
C -5.340157 -2.450321 -0.140644
N -4.784351 -2.835968 1.123581
H -6.144350 -3.137268 -0.438354
C -4.395644 -4.177900 1.498034
N -3.090830 -4.603775 1.049377
H -5.150795 -4.874948 1.113604
H -4.376589 -4.214646 2.589237
C -1.993674 -4.643293 1.908151
N -0.896652 -5.049573 1.149313
O -2.009283 -4.416331 3.097855
C 0.326509 -5.460193 1.810494
N 1.529680 -4.829101 1.327279
H 0.450487 -6.549733 1.716574
H 0.210385 -5.192698 2.863025
C 2.162736 -3.800137 2.017917
N 3.369988 -3.546539 1.359715
O 1.769126 -3.261474 3.029313
C 3.636727 -4.516581 0.315482
N 3.870730 -3.983121 -1.002807
H 4.461989 -5.181924 0.606614
C 5.088129 -3.350614 -1.439005
N 5.279448 -1.981589 -1.014870
H 5.069756 -3.347373 -2.530356
H 5.940537 -3.940330 -1.082170

C 5.781231 -1.561783 0.269671
C 5.998116 -0.026481 0.078659
H 6.699637 -2.111446 0.516665
H 7.046090 0.255753 -0.090550
N 5.513567 0.527307 1.311800
C 5.611426 1.910733 1.705905
H 6.619450 2.267549 1.454632
H 5.462079 1.952883 2.786794
N 4.624610 2.793441 1.118724
C 3.729024 3.494044 1.930391
O 3.534667 3.313931 3.111087
N 3.148462 4.477642 1.135030
C 2.218404 5.441394 1.674071
H 2.559859 6.453436 1.419035
H 2.230049 5.307959 2.757898
N 0.849352 5.306707 1.234762
C -0.108591 4.645267 1.997084
O 0.083520 4.070804 3.046673
N -1.336369 4.811714 1.352011
C -2.562049 4.628288 2.107463
H -3.086128 5.593109 2.181828
H -2.272937 4.286939 3.104040
N -3.470572 3.649392 1.572587
C -3.655001 2.411775 2.177873
O -3.067829 1.991074 3.151026
N -4.677102 1.766410 1.483756
C -5.330509 0.604032 2.038346
H -6.413409 0.797335 2.092344
H -4.933425 0.450947 3.044728
N -5.091373 -0.622982 1.308412
C -4.612834 -1.750905 1.980773
O -4.178012 -1.790881 3.109873
C -5.818588 -0.986962 0.105549
N -5.426044 -0.307027 -1.104854
H -6.903124 -0.893876 0.255688
C -4.511269 -1.035718 -1.836356
O -3.945809 -0.658303 -2.853389
C -5.837112 1.023607 -1.490133
N -5.081170 2.100961 -0.909000
H -6.895001 1.137860 -1.222417
H -5.718684 1.103452 -2.572269
C -4.190387 2.851917 -1.661031
N -3.739899 3.884682 -0.849409
O -3.894272 2.666557 -2.825667
C -3.077072 5.032974 -1.421538
N -1.694406 5.202396 -1.048782
H -3.620003 5.943797 -1.136019
H -3.105230 4.909951 -2.505943
C -0.662332 4.696014 -1.817599
N 0.522928 5.018481 -1.174469
O -0.762695 4.112785 -2.883798
C 1.778944 4.915679 -1.867370
N 2.741063 4.038471 -1.238145
H 2.231722 5.914661 -1.961563
H 1.561884 4.520112 -2.861451
C 3.177239 2.884018 -1.864452
N 4.389336 2.536808 -1.302541
O 2.609447 2.299147 -2.778789
C 4.761095 3.385102 -0.192012
C 3.659281 4.494920 -0.207903
H 5.779285 3.771038 -0.340879

H 4.041969 5.492183 -0.466778
C 5.235148 1.497742 -1.835716
N 5.196187 0.250345 -1.109379
H 6.265836 1.880049 -1.863095
H 4.896872 1.282415 -2.851237
C 4.862169 -0.917340 -1.792583
O 4.336055 -0.977881 -2.887834
C 0.310175 5.832732 0.012815
C -1.234164 5.715860 0.219051
H 0.659811 6.860350 -0.156917
H -1.724270 6.674054 0.439616
C -4.357008 3.878863 0.462290
C -5.247150 2.588665 0.440226
H -4.930123 4.805046 0.612465
H -6.309551 2.784325 0.643874
C 4.752259 -0.386183 2.037056
N 4.839026 -1.605841 1.375611
O 4.158359 -0.158292 3.070490
C 4.422238 -2.823622 2.035853
H 5.295204 -3.484739 2.156699
H 4.040496 -2.536520 3.018435
C 2.745976 -4.045908 -1.814093
N 1.776415 -4.745363 -1.105855
O 2.641580 -3.616924 -2.946098
C 2.265037 -5.248351 0.165403
H 2.350246 -6.344212 0.137817
C 0.650807 -5.314253 -1.799099
H 0.717082 -4.981768 -2.836801
H 0.703683 -6.412287 -1.758333
C -2.765074 -5.011099 -0.286307
H -3.470971 -5.775300 -0.641814
C 1.032243 -0.451445 -2.250929
C 0.942227 -0.327869 -0.768147
H 0.668736 -1.428745 -2.576882
H 2.047580 -0.302594 -2.617985
C 1.917607 -0.033068 0.225226
C 1.317244 -0.255716 1.441213
H 2.874603 0.430903 0.031332
H 1.701114 -0.047400 2.429505
C -0.024562 -0.652201 1.133176
O -0.041659 -1.100089 -0.176838
N 0.136486 0.604550 -2.886644
C -0.929984 1.082049 -1.923202
H -0.365684 0.176082 -3.705560
H 0.721491 1.403479 -3.162839
H -1.639707 0.258582 -1.819553
H -1.430172 1.919545 -2.416300
C -0.290416 1.424443 -0.611413
C -0.916343 1.141409 0.619027
H 0.442189 2.228065 -0.645846
H -1.915293 0.710624 0.606910
H -0.716356 1.763234 1.485914
O -1.539799 -0.907293 -4.374357
H -1.380898 -1.795552 -4.000634
H -2.425455 -0.677683 -4.034950
Cl -1.084593 -1.505445 2.205869
151
1c+CB[7]+wat PS
C 1.900974 -5.339534 0.092184
N 1.168688 -4.845616 1.232327
H 1.934607 -6.438371 0.119152

C 1.813294 -3.811601 1.870459
N 3.047166 -3.646215 1.266062
O 1.391173 -3.204396 2.846908
C 4.115956 -2.962275 1.949486
N 4.604356 -1.790258 1.268028
H 4.951838 -3.664161 2.096434
H 3.743089 -2.643955 2.923908
C 5.582999 -1.843244 0.185433
N 5.098474 -2.266312 -1.095655
H 6.447804 -2.447082 0.493550
C 4.857696 -3.637084 -1.492060
N 3.598673 -4.202164 -1.070024
H 5.674893 -4.257884 -1.103274
H 4.860543 -3.658760 -2.583878
C 2.517313 -4.328702 -1.941586
N 1.463517 -4.867694 -1.205522
O 2.519730 -4.069437 -3.124571
C 0.289994 -5.374406 -1.887018
N -0.972256 -4.891256 -1.382697
H 0.278542 -6.474146 -1.839101
H 0.377973 -5.052689 -2.927043
C -1.716475 -3.922482 -2.047336
N -2.954378 -3.841789 -1.403081
O -1.379985 -3.303742 -3.033668
C -3.111790 -4.861278 -0.385903
N -3.403626 -4.388963 0.944681
H -3.859728 -5.605032 -0.695915
C -4.683654 -3.909089 1.393932
N -5.033082 -2.562382 0.997198
H -4.662095 -3.926307 2.485107
H -5.465661 -4.583554 1.025865
C -5.580942 -2.179195 -0.280069
C -5.976481 -0.685835 -0.058790
H -6.427469 -2.828518 -0.541209
H -7.046177 -0.533833 0.138478
N -5.589811 -0.060655 -1.291418
C -5.868831 1.304661 -1.661840
H -6.904905 1.529845 -1.375492
H -5.759901 1.378536 -2.745813
N -4.980999 2.295570 -1.092571
C -4.185959 3.092253 -1.917262
O -4.015783 2.954989 -3.107226
N -3.677337 4.114317 -1.119847
C -2.871702 5.174911 -1.674117
H -3.306301 6.145300 -1.400683
H -2.901426 5.048120 -2.758328
N -1.483936 5.182407 -1.273511
C -0.495553 4.541248 -2.014296
O -0.660037 3.903049 -3.030987
N 0.724725 4.809611 -1.387278
C 1.953460 4.664851 -2.140811
H 2.387636 5.661616 -2.321911
H 1.690264 4.200607 -3.094213
N 2.942763 3.823319 -1.512845
C 3.416774 2.685741 -2.169906
O 2.910895 2.148523 -3.130731
N 4.599473 2.315390 -1.540060
C 5.395979 1.204628 -2.001096
H 6.454378 1.501899 -1.967366
H 5.103338 0.998346 -3.033158
N 5.210168 -0.031051 -1.265670

C 4.874338 -1.199064 -1.961600
O 4.511978 -1.274268 -3.113471
C 5.914809 -0.333813 -0.032693
N 5.403521 0.285716 1.166611
H 6.989389 -0.127322 -0.132937
C 4.551758 -0.546588 1.861182
O 3.908821 -0.234330 2.855747
C 5.583960 1.667724 1.536279
N 4.682668 2.593120 0.893604
H 6.621848 1.944699 1.308360
H 5.408487 1.746862 2.610733
C 3.798873 3.354237 1.650505
N 3.346482 4.381972 0.836312
O 3.507492 3.175201 2.816935
C 2.504272 5.435563 1.345137
N 1.110194 5.369942 0.971614
H 2.900004 6.401197 1.007049
H 2.549165 5.377348 2.434264
C 0.153540 4.774307 1.774918
N -1.067923 4.911464 1.128463
O 0.334640 4.253711 2.862415
C -2.295631 4.690009 1.849848
N -3.177425 3.718926 1.244381
H -2.843075 5.639860 1.949993
H -2.018936 4.324560 2.840626
C -3.461942 2.515455 1.866613
N -4.628838 2.025860 1.315153
O -2.813585 2.000102 2.769020
C -5.131579 2.845297 0.233440
C -4.159814 4.071412 0.233312
H -6.179977 3.113320 0.426033
H -4.646595 5.019107 0.503798
C -5.341608 0.895308 1.857665
N -5.182939 -0.334665 1.117207
H -6.407449 1.160733 1.913501
H -4.957741 0.710349 2.862920
C -4.720846 -1.469010 1.783777
O -4.175692 -1.485148 2.871343
C -0.967064 5.749291 -0.062126
C 0.572080 5.769381 -0.305317
H -1.403228 6.740099 0.123848
H 0.963492 6.754688 -0.593162
C 3.865973 4.306528 -0.508139
C 4.926159 3.158541 -0.421525
H 4.290568 5.275896 -0.805853
H 5.964402 3.511407 -0.496191
C -4.710889 -0.853426 -2.027129
N -4.638204 -2.084696 -1.383304
O -4.146869 -0.533377 -3.052317
C -4.078409 -3.229143 -2.069844
H -4.867661 -3.984039 -2.215138
H -3.727539 -2.873652 -3.041471
C -2.284753 -4.371880 1.766899
N -1.248879 -4.952202 1.048069
O -2.233027 -3.974437 2.914104
C -1.667420 -5.443320 -0.250915
H -1.630142 -6.541958 -0.275928
C -0.064117 -5.408608 1.723506
H -0.168779 -5.123900 2.772182
H 0.012440 -6.502890 1.642740
C 3.299874 -4.666321 0.252517

H 4.081361 -5.353032 0.608425
C -0.986640 -0.529926 2.298462
C -0.766592 -0.237069 0.827434
H -0.600324 -1.515028 2.567070
H -2.026448 -0.445776 2.610390
C -1.842353 -0.302768 -0.236710
C -1.185837 -0.423423 -1.395260
H -2.896183 -0.134414 -0.062049
H -1.567392 -0.414748 -2.407696
C 0.291408 -0.439979 -1.011309
O 0.265369 -1.085635 0.272933
N -0.169972 0.532011 3.019219
C 0.771365 1.161088 2.012623
H 0.414043 0.086404 3.779943
H -0.806953 1.248287 3.384345
H 1.628916 0.490437 1.937827
H 1.093594 2.130477 2.391212
C -0.045730 1.166673 0.725637
C 0.725892 1.012037 -0.604381
H -0.760941 1.993844 0.720653
H 1.808744 1.023333 -0.454110
H 0.469688 1.749051 -1.367009
O 1.635287 -0.879590 4.424747
H 1.540025 -1.763782 4.017894
H 2.485819 -0.560862 4.065750
Cl 1.402192 -1.254630 -2.119056
151
1d+CB[7]+wat RS
C 0.604301 -5.567324 0.308819
N 0.039721 -4.817028 1.405164
H 0.368720 -6.634683 0.430391
C 0.950313 -3.978753 2.005983
N 2.174018 -4.174601 1.393369
O 0.727123 -3.243633 2.960568
C 3.394574 -3.765880 2.045273
N 4.117426 -2.736743 1.347668
H 4.042923 -4.647374 2.165190
H 3.137657 -3.372159 3.029993
C 4.993394 -3.000796 0.213444
N 4.348193 -3.317736 -1.028972
H 5.730571 -3.772185 0.475306
C 3.827889 -4.618786 -1.396391
N 2.491037 -4.912344 -0.936941
H 4.518425 -5.380898 -1.013859
H 3.795212 -4.661584 -2.487075
C 1.397869 -4.895171 -1.799664
N 0.261607 -5.116354 -1.024653
O 1.441279 -4.756681 -3.003173
C -1.011044 -5.394263 -1.663627
N -2.118860 -4.593152 -1.202073
H -1.272308 -6.454159 -1.523245
H -0.871366 -5.188556 -2.727028
C -2.648090 -3.542066 -1.947669
N -3.805090 -3.114448 -1.288530
O -2.215092 -3.116886 -2.996203
C -4.169244 -3.998496 -0.198238
N -4.332798 -3.384398 1.094862
H -5.065865 -4.580447 -0.455502
C -5.482910 -2.627782 1.516474
N -5.532785 -1.254999 1.069711
H -5.468759 -2.606624 2.607684

H -6.387689 -3.137255 1.165019
C -5.963514 -0.811833 -0.232401
C -5.993626 0.745250 -0.081831
H -6.941065 -1.249677 -0.476432
H -7.004198 1.157372 0.043754
N -5.407366 1.203956 -1.310860
C -5.321014 2.578539 -1.741100
H -6.278036 3.069297 -1.516986
H -5.151659 2.571690 -2.819941
N -4.236802 3.342053 -1.157186
C -3.250359 3.911728 -1.967606
O -3.066395 3.688542 -3.142500
N -2.560152 4.828825 -1.179300
C -1.517594 5.671407 -1.717407
H -1.751725 6.720247 -1.489620
H -1.521765 5.516441 -2.798507
N -0.180289 5.395330 -1.246264
C 0.737167 4.685173 -2.015185
O 0.518531 4.157523 -3.083840
N 1.963170 4.737902 -1.349596
C 3.181943 4.436248 -2.077744
H 3.819030 5.332415 -2.099159
H 2.885058 4.168713 -3.094290
N 3.954835 3.337114 -1.560659
C 3.934320 2.080217 -2.152924
O 3.297265 1.755239 -3.131520
N 4.820993 1.275695 -1.438003
C 5.283990 0.023571 -1.987852
H 6.381892 0.054171 -2.074563
H 4.840068 -0.081614 -2.980628
N 4.894965 -1.150652 -1.230258
C 4.267531 -2.218272 -1.877704
O 3.783361 -2.207031 -2.989586
C 5.614407 -1.594883 -0.047436
N 5.330753 -0.879774 1.172456
H 6.697500 -1.615044 -0.231257
C 4.367452 -1.510798 1.934344
O 3.867418 -1.082300 2.964093
C 5.898621 0.394607 1.545494
N 5.282920 1.550207 0.950695
H 6.963743 0.379098 1.281813
H 5.786431 0.496889 2.626405
C 4.475288 2.403645 1.687460
N 4.182137 3.490695 0.876691
O 4.134165 2.249389 2.844857
C 3.656111 4.709143 1.446418
N 2.305424 5.043555 1.068064
H 4.305628 5.548611 1.165110
H 3.663524 4.583121 2.530802
C 1.217509 4.640019 1.820266
N 0.079520 5.094875 1.169115
O 1.241669 4.033173 2.876919
C -1.187903 5.120170 1.847028
N -2.234554 4.356855 1.199565
H -1.531857 6.161384 1.946809
H -1.026128 4.694825 2.839343
C -2.839073 3.287949 1.838809
N -4.084756 3.109226 1.272645
O -2.368930 2.641770 2.768311
C -4.317126 3.975388 0.138815
C -3.076023 4.925336 0.157224

H -5.276217 4.498550 0.258569
H -3.322749 5.967004 0.406718
C -5.080628 2.216219 1.814813
N -5.206152 0.953793 1.127837
H -6.045571 2.742883 1.806298
H -4.796678 1.986384 2.843698
C -5.019310 -0.227836 1.840024
O -4.531060 -0.322825 2.949937
C 0.387029 5.877632 -0.017297
C 1.919591 5.621055 -0.197410
H 0.130007 6.934145 0.141362
H 2.500333 6.534186 -0.387277
C 4.828096 3.414576 -0.419917
C 5.515168 2.005480 -0.398309
H 5.536784 4.246910 -0.535731
H 6.593918 2.039453 -0.607315
C -4.795800 0.177760 -2.024488
N -5.027671 -1.004586 -1.329872
O -4.200334 0.299151 -3.075385
C -4.771559 -2.281108 -1.966772
H -5.720894 -2.831868 -2.061744
H -4.372481 -2.065275 -2.960646
C -3.217382 -3.536391 1.905268
N -2.333652 -4.364763 1.224676
O -3.059727 -3.078311 3.020073
C -2.886760 -4.870235 -0.018806
H -3.093792 -5.947167 0.062680
C -1.280124 -5.031197 1.942603
H -1.294374 -4.642304 2.962541
H -1.467266 -6.115579 1.957943
C 2.131665 -5.249919 0.411034
H 2.733079 -6.097559 0.770151
C -1.136244 -0.407395 2.409726
C -1.255888 -0.487693 0.942048
H -0.759286 -1.348869 2.810757
H -2.099671 -0.181187 2.869213
C -2.078185 0.078864 0.009899
C -1.568725 -0.276211 -1.276385
H -2.929471 0.708424 0.219203
H -1.960080 -0.012078 -2.246781
C -0.462160 -1.033537 -1.029824
O -0.263635 -1.205351 0.304124
N -0.158142 0.680779 2.873267
C 1.059625 0.955224 2.014591
H 0.245256 0.332042 3.770860
H -0.687022 1.554716 3.009908
H 1.558373 -0.002560 1.860453
H 1.702371 1.585338 2.637861
C 0.743642 1.642380 0.720529
C 1.333600 1.337678 -0.435624
H 0.032146 2.463514 0.767038
H 2.037662 0.513829 -0.520421
H 1.130102 1.883862 -1.350669
O 1.406126 -0.879707 4.470679
H 1.199237 -1.765057 4.116921
H 2.316285 -0.727399 4.157164
Br 0.806100 -1.764846 -2.167888
151
1d+CB[7]+wat TS
C 2.866005 -4.854154 0.287902
N 2.039463 -4.453068 1.399457

H 3.086207 -5.929263 0.355232
C 2.484701 -3.299700 2.003263
N 3.683888 -2.948551 1.409474
O 1.943914 -2.732839 2.945153
C 4.604522 -2.062393 2.079078
N 4.863481 -0.835971 1.371551
H 5.554920 -2.594554 2.240193
H 4.175272 -1.796841 3.046369
C 5.831406 -0.726340 0.284260
N 5.445564 -1.290303 -0.976713
H 6.805218 -1.121079 0.605056
C 5.495961 -2.694245 -1.321989
N 4.366270 -3.487468 -0.897664
H 6.412225 -3.120774 -0.895924
H 5.527745 -2.757431 -2.411585
C 3.340529 -3.831181 -1.775336
N 2.376635 -4.508573 -1.032344
O 3.322308 -3.621107 -2.969160
C 1.328505 -5.242967 -1.714023
N -0.009574 -4.981988 -1.246072
H 1.516373 -6.324074 -1.627638
H 1.378812 -4.944148 -2.763190
C -0.902401 -4.174846 -1.944623
N -2.142553 -4.286541 -1.306002
O -0.664818 -3.541442 -2.949624
C -2.132545 -5.308783 -0.276469
N -2.541824 -4.889732 1.039842
H -2.717020 -6.184008 -0.594324
C -3.901828 -4.666120 1.455305
N -4.499124 -3.421156 1.028618
H -3.903100 -4.664274 2.546798
H -4.524781 -5.487849 1.083203
C -5.076507 -3.167127 -0.267035
C -5.770604 -1.781507 -0.076073
H -5.768270 -3.975397 -0.540403
H -6.855378 -1.846377 0.083238
N -5.475210 -1.098281 -1.303445
C -6.006253 0.182833 -1.699599
H -7.073852 0.203460 -1.442931
H -5.883783 0.267502 -2.781360
N -5.344706 1.332277 -1.120529
C -4.693971 2.262264 -1.933343
O -4.461860 2.148628 -3.115469
N -4.418883 3.369833 -1.136612
C -3.818087 4.564243 -1.680653
H -4.438232 5.432427 -1.421245
H -3.799139 4.432591 -2.764565
N -2.466671 4.836858 -1.251884
C -1.360864 4.442477 -1.999006
O -1.383579 3.815792 -3.035936
N -0.228206 4.948169 -1.356496
C 1.004171 5.089247 -2.109195
H 1.233434 6.159562 -2.227508
H 0.830324 4.638652 -3.089045
N 2.149086 4.431880 -1.536493
C 2.719042 3.307076 -2.122612
O 2.294585 2.699899 -3.081868
N 3.896556 3.037285 -1.427206
C 4.882921 2.127701 -1.959754
H 5.850580 2.650081 -2.020034
H 4.558479 1.837893 -2.961967

N 5.038491 0.901355 -1.205575
C 4.952561 -0.330428 -1.856916
O 4.571003 -0.526739 -2.990006
C 5.831054 0.807896 0.008843
N 5.234171 1.352995 1.203330
H 6.833305 1.232791 -0.140140
C 4.576577 0.389795 1.940718
O 3.905060 0.589681 2.943154
C 5.204438 2.751324 1.564056
N 4.153554 3.524745 0.958904
H 6.176074 3.186370 1.297756
H 5.056814 2.809558 2.644010
C 3.081979 3.999610 1.700232
N 2.350116 4.835625 0.868716
O 2.853627 3.763863 2.871163
C 1.368254 5.742726 1.412805
N 0.001913 5.482693 1.032770
H 1.615480 6.768449 1.108196
H 1.423290 5.656054 2.499673
C -0.842057 4.711269 1.810877
N -2.065719 4.659667 1.159231
O -0.582952 4.204672 2.889258
C -3.240834 4.208431 1.856400
N -3.909294 3.084045 1.240974
H -3.962449 5.035540 1.939207
H -2.920218 3.904788 2.854811
C -3.974747 1.851471 1.865863
N -5.020724 1.151009 1.298030
O -3.260301 1.466381 2.783334
C -5.638119 1.852618 0.193826
C -4.916732 3.240635 0.205555
H -6.723577 1.917495 0.353316
H -5.580217 4.079959 0.457328
C -5.521202 -0.089804 1.837128
N -5.106653 -1.272847 1.121051
H -6.619172 -0.032571 1.857917
H -5.140308 -0.188642 2.855589
C -4.438871 -2.282055 1.810606
O -3.933605 -2.181599 2.912847
C -2.095015 5.496118 -0.033030
C -0.581321 5.815212 -0.244108
H -2.718196 6.385124 0.135085
H -0.381104 6.867195 -0.489770
C 2.930659 4.966742 -0.451787
C 4.174528 4.014560 -0.399784
H 3.189889 6.017526 -0.646215
H 5.127123 4.524041 -0.603811
C -4.444087 -1.711412 -2.011097
N -4.141900 -2.896375 -1.348511
O -3.936452 -1.298026 -3.032853
C -3.349262 -3.911882 -2.007476
H -3.970020 -4.808974 -2.163471
H -3.049507 -3.501386 -2.974523
C -1.465841 -4.617211 1.873331
N -0.317420 -4.981868 1.180894
O -1.516264 -4.189135 3.009430
C -0.610308 -5.603500 -0.098191
H -0.368599 -6.675814 -0.069447
C 0.911815 -5.200094 1.896470
H 0.735034 -4.894464 2.929448
H 1.177871 -6.267226 1.866989

C 4.126456 -3.945936 0.439938
H 5.006792 -4.475132 0.831518
C -0.927558 -0.706892 2.353523
C -0.877018 -0.578428 0.871857
H -0.282163 -1.522229 2.688215
H -1.940890 -0.873147 2.718597
C -1.879670 -0.549378 -0.126324
C -1.226253 -0.576051 -1.341367
H -2.931071 -0.379480 0.057905
H -1.643584 -0.472498 -2.332440
C 0.160789 -0.587541 -1.021963
O 0.299461 -1.003011 0.287607
N -0.399562 0.577178 2.983183
C 0.497561 1.345915 2.034743
H 0.191815 0.327513 3.813993
H -1.201888 1.167608 3.236384
H 1.416662 0.763733 1.942725
H 0.726603 2.289536 2.537351
C -0.186051 1.506965 0.713135
C 0.504134 1.399616 -0.498193
H -1.129735 2.046730 0.732609
H 1.580850 1.248720 -0.476469
H 0.147120 1.918185 -1.381714
O 1.667367 -0.342968 4.472266
H 1.808049 -1.227482 4.082407
H 2.436151 0.163827 4.150932
Br 1.555036 -1.101573 -2.185104
151
1d+CB[7]+wat PS
C 3.126125 -4.702633 0.203341
N 2.280451 -4.397412 1.329984
H 3.408195 -5.765182 0.225004
C 2.646814 -3.234429 1.966679
N 3.817568 -2.784117 1.381268
O 2.073041 -2.739414 2.928597
C 4.676601 -1.858234 2.076008
N 4.877370 -0.607217 1.390947
H 5.654862 -2.335436 2.243148
H 4.220229 -1.634563 3.041255
C 5.859557 -0.423474 0.326104
N 5.532035 -0.980648 -0.953486
H 6.844925 -0.774478 0.662374
C 5.656854 -2.374480 -1.321134
N 4.561817 -3.228701 -0.928580
H 6.585512 -2.765185 -0.887318
H 5.709326 -2.413962 -2.411071
C 3.547649 -3.579385 -1.818588
N 2.617885 -4.327444 -1.101320
O 3.518625 -3.324533 -3.003136
C 1.594819 -5.072765 -1.805837
N 0.252462 -4.892719 -1.311304
H 1.829174 -6.148032 -1.773568
H 1.619863 -4.722726 -2.840003
C -0.686204 -4.109922 -1.975004
N -1.920334 -4.334056 -1.355227
O -0.486218 -3.412326 -2.945419
C -1.849490 -5.384436 -0.359185
N -2.268340 -5.023229 0.971858
H -2.394651 -6.276807 -0.698757
C -3.633744 -4.880650 1.402166
N -4.303033 -3.663012 1.001061

H -3.623878 -4.897563 2.493520
H -4.217158 -5.727398 1.021553
C -4.907026 -3.422305 -0.285188
C -5.684284 -2.087982 -0.062940
H -5.548057 -4.267716 -0.569402
H -6.758081 -2.223839 0.122980
N -5.463444 -1.376079 -1.288758
C -6.090663 -0.131593 -1.660128
H -7.148234 -0.184555 -1.369549
H -6.009272 -0.033324 -2.744549
N -5.489013 1.056489 -1.096604
C -4.907576 2.018706 -1.921707
O -4.712060 1.926609 -3.112140
N -4.655966 3.131842 -1.124044
C -4.118385 4.349617 -1.678999
H -4.763530 5.194317 -1.404585
H -4.120792 4.218995 -2.763145
N -2.768987 4.676252 -1.280305
C -1.658532 4.254141 -2.005329
O -1.671371 3.579452 -3.011402
N -0.530544 4.791892 -1.376677
C 0.702458 4.912382 -2.128548
H 0.893309 5.976306 -2.345397
H 0.558817 4.368064 -3.064947
N 1.858523 4.347211 -1.475619
C 2.600624 3.343651 -2.104592
O 2.246037 2.670600 -3.047230
N 3.837192 3.295007 -1.470671
C 4.895995 2.426081 -1.922250
H 5.838511 2.992988 -1.903001
H 4.664128 2.131062 -2.948125
N 5.046016 1.198319 -1.166539
C 5.046378 -0.025919 -1.842394
O 4.738887 -0.214243 -2.998184
C 5.789313 1.115422 0.078820
N 5.118339 1.601597 1.260125
H 6.773849 1.593637 -0.017849
C 4.494946 0.589305 1.958848
O 3.775256 0.740948 2.938144
C 4.943937 2.988510 1.612795
N 3.845401 3.650316 0.953055
H 5.881886 3.513438 1.387336
H 4.744257 3.034738 2.684950
C 2.800516 4.188917 1.694653
N 2.109303 5.050590 0.856384
O 2.561218 3.974790 2.867214
C 1.039090 5.883505 1.344429
N -0.296297 5.483086 0.968159
H 1.196755 6.909730 0.989983
H 1.090005 5.855827 2.434493
C -1.092446 4.692194 1.778554
N -2.307185 4.529726 1.125254
O -0.802078 4.246243 2.875454
C -3.453163 4.030751 1.842949
N -4.077058 2.874212 1.242643
H -4.210156 4.824997 1.931792
H -3.104189 3.749218 2.838311
C -4.057973 1.637594 1.864747
N -5.066093 0.875016 1.308863
O -3.305979 1.297758 2.769714
C -5.759249 1.549878 0.231954

C -5.113895 2.975582 0.229718
H -6.840025 1.555308 0.431796
H -5.816512 3.777204 0.497962
C -5.483646 -0.393838 1.854770
N -5.021210 -1.550143 1.123680
H -6.582384 -0.398177 1.902095
H -5.074320 -0.474735 2.863753
C -4.291966 -2.530557 1.794445
O -3.774583 -2.413240 2.889540
C -2.396867 5.356314 -0.074895
C -0.902172 5.719343 -0.318207
H -3.045285 6.225250 0.100995
H -0.742929 6.760488 -0.630615
C 2.632766 5.069999 -0.488510
C 3.943526 4.223073 -0.377472
H 2.804762 6.106018 -0.813805
H 4.862487 4.819496 -0.466989
C -4.389924 -1.897075 -2.008663
N -4.000292 -3.068513 -1.366768
O -3.914194 -1.428655 -3.021557
C -3.145074 -4.014842 -2.050197
H -3.708746 -4.944085 -2.232855
H -2.870929 -3.559629 -3.004698
C -1.200147 -4.752250 1.816440
N -0.044019 -5.057068 1.111338
O -1.264098 -4.371896 2.968928
C -0.312148 -5.612000 -0.201482
H -0.016166 -6.670362 -0.239075
C 1.204276 -5.231393 1.803188
H 1.021952 -4.983413 2.850535
H 1.533155 -6.277906 1.722411
C 4.330950 -3.730656 0.393829
H 5.234478 -4.218838 0.786056
C -0.917981 -0.715139 2.394947
C -0.765594 -0.361714 0.928732
H -0.310076 -1.583704 2.655844
H -1.949583 -0.882379 2.699682
C -1.789881 -0.657549 -0.148228
C -1.113173 -0.605904 -1.300815
H -2.855934 -0.736064 0.015217
H -1.477911 -0.668152 -2.317477
C 0.323369 -0.293295 -0.899416
O 0.437461 -0.945648 0.375130
N -0.377468 0.501507 3.132327
C 0.396866 1.345072 2.139887
H 0.291860 0.200186 3.892409
H -1.166375 1.044695 3.497880
H 1.388551 0.895766 2.068270
H 0.478240 2.360424 2.526588
C -0.387944 1.171551 0.845984
C 0.412914 1.211738 -0.474826
H -1.275192 1.810008 0.838350
H 1.461982 1.469593 -0.308123
H 0.003871 1.879209 -1.234101
O 1.727653 -0.440032 4.519072
H 1.850986 -1.317214 4.104668
H 2.471378 0.080047 4.158518
Br 1.715246 -0.853007 -2.107616
22
noCBpath coordinate 3.03
C1 -0.057073 -0.570312 -0.040718

C2 1.081693 -1.086698 -0.910657
C3 2.150462 -0.378341 -0.525626
C4 1.669826 0.539811 0.599378
O 0.639537 -0.300279 1.200406
H1 0.979226 -1.797347 -1.724301
H2 3.146523 -0.374051 -0.953483
C5 -1.363822 -1.314944 0.217094
H3 -1.526896 -2.196753 -0.402902
H4 -1.453886 -1.586630 1.270057
N -2.431603 -0.280583 -0.107359
H5 -3.314309 -0.424952 0.437530
C6 -1.784460 1.075212 0.115459
H6 -2.389482 1.852830 -0.352485
H7 -1.742205 1.240381 1.194943
C7 -0.406178 0.834288 -0.504133
C8 0.818836 1.643793 0.021552
H8 0.531556 2.345194 0.811837
H9 1.331875 2.201557 -0.766779
H10 -0.497045 0.884463 -1.594789
H11 -2.691100 -0.382553 -1.117256
H12 2.392059 0.852035 1.354966
22
noCBpath coordinate 3.19
C1 -0.043995 -0.602599 -0.033298
C2 1.091999 -1.100848 -0.903003
C3 2.158960 -0.389586 -0.514437
C4 1.683717 0.513642 0.614189
O 0.636106 -0.302464 1.196911
H1 0.997184 -1.797884 -1.728747
H2 3.149267 -0.372092 -0.955459
C5 -1.359438 -1.327072 0.217548
H3 -1.529472 -2.204076 -0.408219
H4 -1.465056 -1.609901 1.267146
N -2.420522 -0.287586 -0.108253
H5 -3.304585 -0.430681 0.432485
C6 -1.788984 1.077885 0.106936
H6 -2.427963 1.836832 -0.347016
H7 -1.733197 1.246189 1.185707
C7 -0.414876 0.879853 -0.528048
C8 0.799773 1.681624 0.001147
H8 0.528287 2.373846 0.801619
H9 1.328480 2.232014 -0.779431
H10 -0.501975 0.920333 -1.617494
H11 -2.676075 -0.392017 -1.119370
H12 2.394017 0.823936 1.379066
22
noCBpath coordinate 3.37
C1 -0.029268 -0.638839 -0.023395
C2 1.099515 -1.114619 -0.896118
C3 2.167878 -0.399522 -0.501841
C4 1.696280 0.483793 0.628935
O 0.634426 -0.307298 1.195491
H1 1.006188 -1.796483 -1.733035
H2 3.152693 -0.367741 -0.951372
C5 -1.354166 -1.337986 0.217193
H3 -1.528370 -2.211780 -0.411283
H4 -1.469676 -1.621727 1.265042
N -2.410078 -0.292594 -0.114082
H5 -3.295337 -0.436598 0.425518
C6 -1.793324 1.080885 0.097545
H6 -2.455737 1.827580 -0.346539

H7 -1.730757 1.249109 1.174915
C7 -0.423498 0.929280 -0.550551
C8 0.774942 1.715064 -0.020267
H8 0.525102 2.396272 0.797514
H9 1.330079 2.255161 -0.790891
H10 -0.508776 0.949074 -1.640654
H11 -2.665697 -0.399588 -1.124715
H12 2.394161 0.810574 1.398207
22
noCBpath coordinate 3.56
C1 -0.013756 -0.681404 -0.010010
C2 1.105895 -1.127937 -0.885330
C3 2.171827 -0.410300 -0.488728
C4 1.708736 0.453156 0.643825
O 0.630997 -0.309664 1.193514
H1 1.011501 -1.794217 -1.736658
H2 3.152703 -0.359604 -0.949620
C5 -1.349534 -1.348643 0.219843
H3 -1.529489 -2.221100 -0.409496
H4 -1.478712 -1.631109 1.266640
N -2.398072 -0.296602 -0.120214
H5 -3.285739 -0.439874 0.416062
C6 -1.795043 1.085076 0.086088
H6 -2.482670 1.816649 -0.345724
H7 -1.724624 1.253563 1.163535
C7 -0.433949 0.985062 -0.576221
C8 0.750393 1.750270 -0.043123
H8 0.524649 2.412861 0.795618
H9 1.334179 2.276752 -0.799344
H10 -0.513433 0.980354 -1.665484
H11 -2.651404 -0.406875 -1.131177
H12 2.391750 0.799041 1.415328
22
noCBpath coordinate 3.77
C1 0.003997 -0.731702 0.008225
C2 1.105780 -1.143063 -0.874834
C3 2.176234 -0.416816 -0.476023
C4 1.719447 0.421387 0.653982
O 0.628363 -0.309166 1.190722
H1 1.010378 -1.789385 -1.739749
H2 3.147791 -0.346801 -0.951850
C5 -1.345526 -1.359725 0.225329
H3 -1.533213 -2.230621 -0.404455
H4 -1.491243 -1.638816 1.271101
N -2.384660 -0.299111 -0.124977
H5 -3.274816 -0.440434 0.407963
C6 -1.794095 1.090127 0.077227
H6 -2.505863 1.806901 -0.340608
H7 -1.716770 1.257871 1.155288
C7 -0.445544 1.046528 -0.601032
C8 0.725570 1.782443 -0.065235
H8 0.528185 2.419656 0.798241
H9 1.340196 2.294011 -0.804062
H10 -0.518639 1.007815 -1.688051
H11 -2.635942 -0.411902 -1.136025
H12 2.388950 0.793303 1.427046
22
noCBpath coordinate 3.98
C1 0.021766 -0.791954 0.029983
C2 1.098781 -1.151957 -0.864303
C3 2.172147 -0.413284 -0.465988

C4 1.722524 0.396978 0.660304
O 0.617927 -0.299304 1.183454
H1 0.999541 -1.778610 -1.743481
H2 3.131273 -0.314973 -0.960538
C5 -1.340560 -1.376307 0.235911
H3 -1.536342 -2.248435 -0.388610
H4 -1.505999 -1.644722 1.281422
N -2.364047 -0.304831 -0.133544
H5 -3.261356 -0.443587 0.388470
C6 -1.789105 1.093156 0.067850
H6 -2.533421 1.788907 -0.331983
H7 -1.699078 1.259020 1.144328
C7 -0.456897 1.122091 -0.630029
C8 0.699528 1.810003 -0.086422
H8 0.540559 2.411645 0.809031
H9 1.356556 2.304600 -0.801042
H10 -0.521576 1.051771 -1.715752
H11 -2.604190 -0.421317 -1.146689
H12 2.371468 0.802253 1.432721
22
noCBpath coordinate 4.20
C1 0.037137 -0.830245 0.044495
C2 1.087528 -1.153265 -0.859364
C3 2.175575 -0.413088 -0.455447
C4 1.748995 0.349554 0.677895
O 0.616543 -0.287216 1.173958
H1 0.986207 -1.762751 -1.749716
H2 3.125382 -0.300277 -0.963726
C5 -1.334797 -1.380627 0.241065
H3 -1.537248 -2.254975 -0.380366
H4 -1.513462 -1.642856 1.286308
N -2.351712 -0.305671 -0.138604
H5 -3.250431 -0.444389 0.380318
C6 -1.788567 1.095638 0.065092
H6 -2.545382 1.783085 -0.326660
H7 -1.697736 1.259635 1.141977
C7 -0.466049 1.173240 -0.642511
C8 0.659492 1.852886 -0.114545
H8 0.546546 2.402068 0.820100
H9 1.359265 2.313065 -0.807273
H10 -0.523328 1.050996 -1.723400
H11 -2.590263 -0.423210 -1.152439
H12 2.371799 0.804039 1.441682
22
noCBpath coordinate 4.41
C1 0.046797 -0.851184 0.059206
C2 1.066054 -1.139263 -0.859855
C3 2.176886 -0.408290 -0.444441
C4 1.780988 0.296455 0.704493
O 0.613637 -0.266726 1.166777
H1 0.947253 -1.717028 -1.766276
H2 3.120245 -0.289151 -0.960952
C5 -1.329080 -1.379672 0.252327
H3 -1.529743 -2.262172 -0.357742
H4 -1.516669 -1.626962 1.299708
N -2.337808 -0.307878 -0.148684
H5 -3.242811 -0.449733 0.357653
C6 -1.791499 1.097188 0.063349
H6 -2.564694 1.773464 -0.317245
H7 -1.694962 1.256568 1.139383
C7 -0.478585 1.222631 -0.656068

C8 0.617645 1.901535 -0.144761
H8 0.561495 2.383893 0.828632
H9 1.364493 2.311602 -0.817872
H10 -0.528741 1.054588 -1.730674
H11 -2.562235 -0.427763 -1.165517
H12 2.372945 0.802751 1.458044
22
noCBpath coordinate 4.62
C1 0.060433 -0.883795 0.069662
C2 1.060129 -1.142423 -0.858664
C3 2.184424 -0.412374 -0.440645
C4 1.809645 0.251055 0.722762
O 0.617128 -0.260779 1.158710
H1 0.944340 -1.714553 -1.770541
H2 3.122554 -0.287397 -0.963315
C5 -1.325909 -1.382574 0.258031
H3 -1.535935 -2.266482 -0.346296
H4 -1.524135 -1.621682 1.305334
N -2.326960 -0.305425 -0.152727
H5 -3.233601 -0.448167 0.351400
C6 -1.796109 1.103476 0.062623
H6 -2.579730 1.771833 -0.309736
H7 -1.697171 1.259056 1.139654
C7 -0.494729 1.270702 -0.666874
C8 0.582388 1.949852 -0.162669
H8 0.557912 2.390195 0.830805
H9 1.365246 2.312537 -0.819793
H10 -0.534109 1.068898 -1.735204
H11 -2.550823 -0.427699 -1.169541
H12 2.379180 0.797294 1.462221
22
noCBpath coordinate 4.82
C1 0.071731 -0.908030 0.081097
C2 1.053893 -1.148043 -0.858060
C3 2.192665 -0.424471 -0.436631
C4 1.836608 0.210063 0.736054
O 0.623088 -0.255295 1.153197
H1 0.934162 -1.716149 -1.771595
H2 3.131905 -0.310838 -0.959265
C5 -1.321247 -1.380928 0.269167
H3 -1.540106 -2.270981 -0.323912
H4 -1.527496 -1.605437 1.318782
N -2.312639 -0.302927 -0.158468
H5 -3.225336 -0.450825 0.332455
C6 -1.805493 1.113034 0.064804
H6 -2.611788 1.764282 -0.291502
H7 -1.697471 1.264138 1.141187
C7 -0.519985 1.327873 -0.679019
C8 0.548283 2.000730 -0.176167
H8 0.546641 2.403755 0.832820
H9 1.350585 2.336766 -0.823640
H10 -0.556260 1.117962 -1.746579
H11 -2.522072 -0.426319 -1.178248
H12 2.398235 0.770286 1.472582
22
noCBpath coordinate 5.02
C1 0.083935 -0.938525 0.094340
C2 1.054605 -1.158926 -0.855492
C3 2.201116 -0.440037 -0.432134
C4 1.864036 0.167578 0.752403
O 0.632912 -0.262514 1.153751

H1 0.930684 -1.720456 -1.772609
H2 3.138009 -0.322381 -0.961452
C5 -1.317776 -1.382460 0.275664
H3 -1.545754 -2.273356 -0.312329
H4 -1.533284 -1.597591 1.325150
N -2.302624 -0.298867 -0.162614
H5 -3.216332 -0.448594 0.326186
C6 -1.812664 1.122376 0.064345
H6 -2.630555 1.765186 -0.279920
H7 -1.698348 1.265727 1.141348
C7 -0.540499 1.376311 -0.690184
C8 0.518426 2.048302 -0.188456
H8 0.537444 2.415514 0.833898
H9 1.339262 2.358901 -0.826647
H10 -0.568452 1.143691 -1.753124
H11 -2.512083 -0.425384 -1.182163
H12 2.410219 0.755850 1.476537
22
noCBpath coordinate 5.22
C1 0.093757 -0.958529 0.104906
C2 1.053536 -1.174402 -0.852980
C3 2.213021 -0.468536 -0.428656
C4 1.892371 0.123245 0.764860
O 0.646623 -0.270832 1.154436
H1 0.925126 -1.735642 -1.769238
H2 3.151427 -0.366172 -0.957681
C5 -1.313597 -1.379364 0.287397
H3 -1.552438 -2.274797 -0.290933
H4 -1.535246 -1.580786 1.338262
N -2.290422 -0.294511 -0.165418
H5 -3.208747 -0.450020 0.310888
C6 -1.823368 1.133686 0.069262
H6 -2.660390 1.759738 -0.259701
H7 -1.697589 1.272070 1.144322
C7 -0.566709 1.428419 -0.698489
C8 0.484051 2.100967 -0.196889
H8 0.512520 2.447745 0.833679
H9 1.307644 2.411486 -0.832908
H10 -0.598148 1.203437 -1.764078
H11 -2.486758 -0.421747 -1.187688
H12 2.443751 0.704344 1.490957
22
noCBpath coordinate 5.41
C1 0.104616 -0.978138 0.116974
C2 1.053166 -1.192790 -0.848845
C3 2.227100 -0.503593 -0.425318
C4 1.923431 0.077093 0.775495
O 0.664998 -0.282356 1.158310
H1 0.916667 -1.751527 -1.765310
H2 3.166047 -0.417992 -0.954942
C5 -1.310387 -1.373239 0.300190
H3 -1.559689 -2.273359 -0.264018
H4 -1.535872 -1.555380 1.354163
N -2.279628 -0.287201 -0.168797
H5 -3.203404 -0.450096 0.298450
C6 -1.836112 1.146086 0.074586
H6 -2.687758 1.760180 -0.236192
H7 -1.699586 1.273798 1.151818
C7 -0.595718 1.480138 -0.702764
C8 0.449753 2.153650 -0.200292
H8 0.490760 2.474176 0.837825

H9 1.275625 2.464619 -0.835693
H10 -0.628999 1.257408 -1.769351
H11 -2.467251 -0.416600 -1.191992
H12 2.478626 0.659285 1.497828
22
noCBpath coordinate 5.61
C1 0.115821 -1.002417 0.130920
C2 1.054456 -1.215328 -0.842850
C3 2.240650 -0.541128 -0.420578
C4 1.952209 0.028710 0.787237
O 0.684955 -0.303216 1.166440
H1 0.909425 -1.768521 -1.761269
H2 3.177145 -0.462979 -0.954941
C5 -1.306606 -1.369814 0.313819
H3 -1.568909 -2.272629 -0.240129
H4 -1.537004 -1.536445 1.369391
N -2.268240 -0.280706 -0.168885
H5 -3.195675 -0.449312 0.289483
C6 -1.846954 1.158911 0.080216
H6 -2.714345 1.758475 -0.214444
H7 -1.698480 1.280361 1.156584
C7 -0.623877 1.528472 -0.708991
C8 0.418158 2.203599 -0.206067
H8 0.468102 2.505435 0.837474
H9 1.245203 2.514415 -0.840484
H10 -0.659787 1.308993 -1.776142
H11 -2.447227 -0.412180 -1.193216
H12 2.511652 0.611270 1.506566
22
noCBpath coordinate 5.81
C1 0.126136 -1.024231 0.143142
C2 1.056422 -1.242684 -0.834868
C3 2.256603 -0.589175 -0.412825
C4 1.982350 -0.025033 0.799889
O 0.705904 -0.327175 1.175665
H1 0.901660 -1.789375 -1.755971
H2 3.191740 -0.522352 -0.950326
C5 -1.302727 -1.365387 0.327694
H3 -1.577848 -2.271555 -0.214953
H4 -1.536148 -1.516182 1.385109
N -2.257626 -0.274812 -0.168091
H5 -3.188248 -0.449299 0.281180
C6 -1.857530 1.170252 0.086995
H6 -2.739853 1.755645 -0.191490
H7 -1.697132 1.286003 1.161975
C7 -0.652922 1.573888 -0.714544
C8 0.385054 2.255637 -0.212277
H8 0.443509 2.540930 0.835439
H9 1.205359 2.577716 -0.849510
H10 -0.695328 1.364978 -1.782756
H11 -2.427186 -0.408031 -1.193776
H12 2.548795 0.552043 1.517993
22
noCBpath coordinate 6.01
C1 0.135180 -1.044942 0.155161
C2 1.060265 -1.276097 -0.824714
C3 2.273035 -0.645505 -0.402096
C4 2.011634 -0.081105 0.812442
O 0.726643 -0.350785 1.183861
H1 0.895667 -1.818569 -1.746723
H2 3.209376 -0.595149 -0.940831

C5 -1.299099 -1.360637 0.342069
H3 -1.587045 -2.270366 -0.188438
H4 -1.533944 -1.494395 1.401226
N -2.247673 -0.269249 -0.166525
H5 -3.181121 -0.449819 0.273416
C6 -1.867000 1.180332 0.095515
H6 -2.763672 1.752294 -0.165438
H7 -1.693916 1.289667 1.168879
C7 -0.682186 1.618939 -0.717921
C8 0.350294 2.306756 -0.218190
H8 0.419887 2.574417 0.833974
H9 1.161194 2.646697 -0.859388
H10 -0.734850 1.424743 -1.789052
H11 -2.407515 -0.403446 -1.193566
H12 2.586264 0.490182 1.528658
22
noCBpath coordinate 6.20
C1 0.144768 -1.068204 0.168082
C2 1.064981 -1.312258 -0.812785
C3 2.289166 -0.701429 -0.390265
C4 2.038330 -0.136533 0.825626
O 0.746974 -0.377755 1.193698
H1 0.892042 -1.851178 -1.735389
H2 3.225436 -0.665469 -0.930209
C5 -1.295011 -1.357904 0.355491
H3 -1.596025 -2.269780 -0.164333
H4 -1.532432 -1.475907 1.415617
N -2.237027 -0.264159 -0.165128
H5 -3.172496 -0.450093 0.267227
C6 -1.875147 1.189880 0.103643
H6 -2.786743 1.747953 -0.137500
H7 -1.688408 1.292481 1.174992
C7 -0.712664 1.661650 -0.722075
C8 0.318539 2.354631 -0.223944
H8 0.399169 2.604978 0.830951
H9 1.118577 2.711289 -0.869730
H10 -0.775483 1.483636 -1.795140
H11 -2.389456 -0.399407 -1.193079
H12 2.619929 0.432352 1.538427
22
noCBpath coordinate 6.40
C1 0.153744 -1.091231 0.181089
C2 1.069406 -1.347693 -0.802080
C3 2.304831 -0.759273 -0.377734
C4 2.066159 -0.196242 0.840788
O 0.767695 -0.407288 1.204803
H1 0.888081 -1.883580 -1.723822
H2 3.241299 -0.739056 -0.918189
C5 -1.290376 -1.355319 0.368511
H3 -1.603899 -2.269144 -0.140838
H4 -1.529311 -1.458963 1.430016
N -2.226120 -0.259985 -0.162806
H5 -3.164057 -0.451466 0.261193
C6 -1.882709 1.197974 0.113087
H6 -2.807749 1.742246 -0.108229
H7 -1.681051 1.294464 1.182108
C7 -0.743484 1.703600 -0.725439
C8 0.283982 2.403417 -0.231654
H8 0.377531 2.638273 0.826040
H9 1.072702 2.776849 -0.882228
H10 -0.819384 1.540025 -1.799800

H11 -2.370627 -0.395368 -1.191837
H12 2.655417 0.366012 1.551806
22
noCBpath coordinate 6.60
C1 0.161734 -1.108315 0.191309
C2 1.072708 -1.381770 -0.790983
C3 2.321104 -0.824367 -0.363754
C4 2.094985 -0.261352 0.856666
O 0.790052 -0.438196 1.216109
H1 0.881599 -1.914637 -1.713744
H2 3.258930 -0.824986 -0.903006
C5 -1.285884 -1.351033 0.382141
H3 -1.610393 -2.267495 -0.115673
H4 -1.523424 -1.439656 1.445471
N -2.216588 -0.256030 -0.159030
H5 -3.157611 -0.454385 0.255958
C6 -1.889726 1.204360 0.125527
H6 -2.825559 1.736950 -0.076652
H7 -1.672478 1.293475 1.192673
C7 -0.774650 1.740583 -0.725330
C8 0.246295 2.454350 -0.239144
H8 0.353976 2.675152 0.819744
H9 1.021124 2.844987 -0.896919
H10 -0.864200 1.587193 -1.800442
H11 -2.352770 -0.390416 -1.188940
H12 2.695369 0.287644 1.568452
22
noCBpath coordinate 6.80
C1 0.170073 -1.126496 0.201756
C2 1.075581 -1.417096 -0.780515
C3 2.337207 -0.892565 -0.349549
C4 2.123714 -0.330833 0.873397
O 0.813593 -0.474293 1.229321
H1 0.874149 -1.943293 -1.705162
H2 3.275301 -0.913408 -0.887959
C5 -1.281041 -1.346888 0.395974
H3 -1.617299 -2.265352 -0.090616
H4 -1.516703 -1.421230 1.460906
N -2.207318 -0.251951 -0.153712
H5 -3.150634 -0.457024 0.252898
C6 -1.896294 1.210731 0.138819
H6 -2.842436 1.731487 -0.044201
H7 -1.663268 1.293904 1.203267
C7 -0.804637 1.774347 -0.724527
C8 0.208041 2.504755 -0.247435
H8 0.327700 2.718678 0.811525
H9 0.968867 2.910315 -0.912791
H10 -0.906300 1.626379 -1.799443
H11 -2.335882 -0.385134 -1.184611
H12 2.735376 0.204845 1.585447
22
noCBpath coordinate 7.00
C1 0.178477 -1.146641 0.212902
C2 1.077607 -1.453206 -0.770421
C3 2.351613 -0.961195 -0.336200
C4 2.150957 -0.402348 0.889928
O 0.837083 -0.513859 1.243657
H1 0.865631 -1.971327 -1.697170
H2 3.289044 -1.001117 -0.874407
C5 -1.275999 -1.343736 0.410298
H3 -1.624593 -2.263383 -0.065597

H4 -1.509760 -1.404728 1.476516
N -2.197488 -0.247967 -0.147482
H5 -3.142776 -0.459059 0.251196
C6 -1.902138 1.217282 0.151589
H6 -2.858393 1.725439 -0.013029
H7 -1.654441 1.296482 1.213065
C7 -0.833733 1.806053 -0.724018
C8 0.170251 2.554121 -0.256635
H8 0.300129 2.766049 0.801543
H9 0.916820 2.973059 -0.929892
H10 -0.946412 1.660941 -1.798131
H11 -2.318786 -0.380347 -1.179283
H12 2.774342 0.118424 1.602788
148
CBpath coordinate 6.59
C1 0.194672 -1.108132 0.213451
C2 1.050275 -1.297904 -0.836003
C3 2.318555 -0.782403 -0.436435
C4 2.152826 -0.320517 0.834894
O 0.864976 -0.499435 1.244118
H1 0.814323 -1.765731 -1.780765
H2 3.236399 -0.792360 -1.007759
C5 -1.248481 -1.353647 0.423560
H3 -1.480999 -1.372850 1.485923
H4 -1.560863 -2.304079 -0.011123
N -2.200426 -0.320299 -0.208483
H5 -3.140870 -0.522177 0.157848
C6 -1.933669 1.162946 0.028106
H6 -1.804297 1.300636 1.103126
H7 -2.856720 1.666799 -0.279158
C7 -0.765581 1.653845 -0.763126
C8 0.219385 2.396288 -0.265534
H8 0.275588 2.663298 0.784349
H9 1.042747 2.711098 -0.893734
H10 -0.750857 1.361598 -1.811301
H11 -2.223423 -0.512714 -1.228381
H12 2.821387 0.110195 1.565150
C1 1.211788 -0.423995 5.171136
N 0.106074 -1.050436 4.482932
C2 -0.258080 -2.423568 4.736964
N 0.187630 -3.348613 3.714996
C3 -0.637825 -3.864918 2.732428
O -1.864918 -3.782994 2.694072
N 0.176156 -4.496573 1.801977
C4 -0.348226 -5.411848 0.816079
N -0.088955 -4.969999 -0.532134
C5 -1.107455 -4.691141 -1.437389
O -2.298537 -4.508521 -1.181075
N -0.527513 -4.628315 -2.692278
C6 -1.140558 -3.913515 -3.793436
N -0.566062 -2.621802 -4.083933
C7 -1.143796 -1.417037 -3.692797
O -1.992448 -1.262277 -2.813336
N -0.656064 -0.425275 -4.538069
C8 -1.452504 0.728069 -4.899823
N -0.884658 2.002411 -4.517267
C9 -1.557344 2.923350 -3.731216
O -2.721993 2.829222 -3.347130
N -0.674250 3.960966 -3.463362
C10 -1.141545 5.272201 -3.067103
N -0.627388 5.709265 -1.789758

C11 -1.428563 6.325000 -0.832388
O -2.652172 6.469933 -0.872697
N -0.593967 6.727279 0.199407
C12 -0.979000 6.563824 1.597267
N -0.411303 5.442368 2.319723
C13 -1.036757 4.210822 2.440587
O -2.070107 3.866915 1.873672
N -0.316273 3.452428 3.363281
C14 -0.958714 2.375896 4.095985
N -0.185236 1.152346 4.155987
H1 -1.950579 2.155112 3.629588
H2 -1.193197 2.715713 5.131052
C15 0.699101 4.257004 4.024086
N 2.085588 3.862667 3.840831
C16 2.703413 2.848948 4.657855
N 2.231612 1.515921 4.378942
C17 3.125368 0.474169 4.160940
O 4.260962 0.564570 3.697362
H3 2.541411 3.093174 5.731386
H4 3.810189 2.875229 4.509579
C18 2.844621 4.900815 3.318822
O 4.071183 4.935826 3.230299
N 1.970309 5.883218 2.889477
C19 2.291545 6.644605 1.704136
N 1.771683 6.094087 0.483305
C20 2.357862 5.068415 -0.237751
O 3.302289 4.369999 0.121917
N 1.744385 5.040786 -1.484249
C21 2.449035 4.558721 -2.649789
N 1.743425 3.507495 -3.339935
C22 2.380624 2.338353 -3.733823
O 3.505596 1.979811 -3.391247
N 1.561064 1.710063 -4.661165
C23 2.095547 0.780559 -5.632926
N 1.642040 -0.574551 -5.429845
C24 2.482655 -1.673454 -5.332495
O 3.709003 -1.675046 -5.410302
N 1.676622 -2.780317 -5.113866
C25 2.162090 -4.013205 -4.531189
N 1.864350 -4.151580 -3.126939
C26 2.792594 -3.876080 -2.132265
O 3.873843 -3.311074 -2.291074
N 2.287564 -4.392354 -0.940223
C27 3.111710 -4.526769 0.246300
N 2.552298 -3.902807 1.432338
C28 3.272085 -2.983920 2.191508
O 4.351171 -2.468859 1.898649
N 2.562433 -2.771816 3.365013
C29 3.081752 -2.010096 4.476444
H5 4.192153 -1.932938 4.396277
H6 2.896714 -2.563626 5.422965
H7 4.118977 -4.086043 0.041774
H8 3.304759 -5.604710 0.444795
H9 3.264559 -4.103980 -4.693966
H10 1.715895 -4.868888 -5.088789
H11 3.214121 0.806167 -5.611735
H12 1.816275 1.108350 -6.660020
H13 3.466444 4.202229 -2.352209
H14 2.629068 5.397470 -3.354072
H15 3.395975 6.784165 1.609186
H16 1.879109 7.669295 1.837710

H17 0.469681 4.373851 5.108236
C30 0.615708 5.590631 3.333929
H18 0.297064 6.361528 4.075116
H19 -2.092749 6.511276 1.674425
H20 -0.702929 7.499219 2.135120
C31 0.774900 6.788402 -0.292146
H21 1.077366 7.854018 -0.436831
C32 0.762472 6.098451 -1.622979
H22 1.077799 6.805790 -2.418301
H23 -2.257673 5.260642 -3.023720
H24 -0.881953 6.019371 -3.850641
C33 0.583949 3.788294 -4.168282
H25 0.785062 4.646223 -4.852484
C34 0.397869 2.523255 -4.962758
H26 0.355625 2.768475 -6.049375
H27 -2.477112 0.627342 -4.461985
H28 -1.619499 0.734314 -6.000299
C35 0.252625 -0.974908 -5.525519
H29 -0.121616 -0.796259 -6.556887
C36 0.271461 -2.444575 -5.253384
H30 -0.162485 -2.986607 -6.127727
H31 -2.238191 -3.799816 -3.612597
H32 -1.065857 -4.554890 -4.701906
C37 0.857429 -5.076304 -2.652214
H33 0.944542 -6.048715 -3.189090
C38 1.167058 -5.283346 -1.194770
H34 1.461775 -6.345413 -1.019672
H35 -1.449628 -5.536397 0.962085
H36 0.085450 -6.426183 0.972427
C39 1.556358 -4.573697 2.251347
H37 1.853554 -5.630954 2.444116
C40 1.558600 -3.797953 3.546944
H38 1.846304 -4.475418 4.385003
H39 -1.367089 -2.498196 4.852443
H40 0.155691 -2.742358 5.721213
C41 -0.785804 -0.094678 4.019073
O -1.921722 -0.312081 3.601957
N 2.514652 -0.687855 4.597870
H41 1.203955 -0.674572 6.257672
C42 1.000320 1.052479 4.990406
H42 0.830899 1.536522 5.980488
148
CBpath coordinate 6.39
C1 0.181477 -1.089176 0.203779
C2 1.043426 -1.293626 -0.838572
C3 2.305505 -0.758904 -0.445696
C4 2.128962 -0.265348 0.812982
O 0.844578 -0.459148 1.225914
H1 0.809956 -1.770780 -1.779139
H2 3.225037 -0.772669 -1.015130
C5 -1.258017 -1.352580 0.419073
H3 -1.485312 -1.365328 1.482893
H4 -1.559783 -2.308867 -0.009665
N -2.222647 -0.329041 -0.208019
H5 -3.157577 -0.525615 0.175829
C6 -1.936955 1.150432 0.027104
H6 -1.811702 1.280258 1.103656
H7 -2.850922 1.670532 -0.280478
C7 -0.751452 1.616565 -0.754284
C8 0.250893 2.332575 -0.244799
H8 0.303936 2.591567 0.806928

H9 1.086700 2.633631 -0.863604
H10 -0.733389 1.327194 -1.802817
H11 -2.262363 -0.525320 -1.226799
H12 2.786047 0.207208 1.528315
C1 1.212248 -0.425496 5.169565
N 0.104585 -1.050605 4.482947
C2 -0.259569 -2.423887 4.736046
N 0.186598 -3.347498 3.713276
C3 -0.638352 -3.863830 2.730310
O -1.865244 -3.780011 2.689807
N 0.176035 -4.499180 1.802924
C4 -0.347498 -5.414725 0.817129
N -0.088534 -4.971561 -0.530606
C5 -1.106608 -4.688414 -1.434858
O -2.297374 -4.504394 -1.177998
N -0.526336 -4.622991 -2.689442
C6 -1.139635 -3.908246 -3.790336
N -0.563833 -2.617490 -4.081699
C7 -1.144063 -1.412803 -3.694579
O -1.996503 -1.258037 -2.818600
N -0.654376 -0.422276 -4.540015
C8 -1.449937 0.731092 -4.902623
N -0.881579 2.003116 -4.515328
C9 -1.554125 2.922731 -3.727320
O -2.719225 2.829311 -3.343717
N -0.671855 3.961578 -3.462474
C10 -1.139839 5.273627 -3.070287
N -0.628952 5.710007 -1.791749
C11 -1.430081 6.324355 -0.833703
O -2.653658 6.469422 -0.874003
N -0.595083 6.724678 0.198688
C12 -0.979765 6.560004 1.596380
N -0.410598 5.438612 2.317970
C13 -1.033703 4.205494 2.438023
O -2.065882 3.859200 1.870329
N -0.315129 3.450560 3.365088
C14 -0.958221 2.377143 4.101712
N -0.186820 1.152399 4.160218
H1 -1.952442 2.158153 3.639534
H2 -1.187843 2.718819 5.137341
C15 0.700444 4.256399 4.023872
N 2.086614 3.861549 3.839953
C16 2.704600 2.846590 4.655037
N 2.230842 1.514518 4.375472
C17 3.123811 0.472817 4.154510
O 4.258564 0.563701 3.688792
H3 2.544492 3.089866 5.729020
H4 3.811194 2.872013 4.505056
C18 2.845578 4.898979 3.316669
O 4.072058 4.933042 3.226524
N 1.971205 5.881563 2.887863
C19 2.292016 6.641986 1.702013
N 1.770526 6.089613 0.482940
C20 2.355406 5.062583 -0.237384
O 3.303069 4.367405 0.120327
N 1.738458 5.031614 -1.481871
C21 2.447143 4.558715 -2.648263
N 1.745218 3.508116 -3.341279
C22 2.383293 2.339931 -3.735412
O 3.507950 1.981327 -3.391851
N 1.564218 1.712442 -4.663520

C23 2.098855 0.782668 -5.634748
N 1.645142 -0.572116 -5.430136
C24 2.486041 -1.670490 -5.329698
O 3.712436 -1.672220 -5.407059
N 1.680077 -2.776824 -5.108934
C25 2.165622 -4.009707 -4.526674
N 1.866231 -4.148395 -3.122971
C26 2.794630 -3.876274 -2.127462
O 3.877083 -3.313056 -2.284289
N 2.288207 -4.394557 -0.937226
C27 3.110557 -4.532729 0.249485
N 2.550414 -3.904095 1.432060
C28 3.265588 -2.976887 2.185316
O 4.340808 -2.456369 1.887962
N 2.560002 -2.769751 3.362178
C29 3.081321 -2.010314 4.473623
H5 4.191736 -1.934032 4.393096
H6 2.895451 -2.563402 5.420352
H7 4.120924 -4.098313 0.046904
H8 3.296243 -5.611782 0.449460
H9 3.268319 -4.099910 -4.688234
H10 1.720270 -4.865291 -5.085049
H11 3.217486 0.808206 -5.613252
H12 1.820027 1.109814 -6.662180
H13 3.466358 4.205637 -2.352993
H14 2.623022 5.401204 -3.349458
H15 3.396377 6.781412 1.605997
H16 1.879564 7.666780 1.834934
H17 0.472142 4.375044 5.108118
C30 0.616440 5.588801 3.332071
H18 0.297325 6.360574 4.072164
H19 -2.093424 6.507011 1.674113
H20 -0.703756 7.495415 2.134292
C31 0.774096 6.784512 -0.292241
H21 1.077246 7.849979 -0.437184
C32 0.761523 6.094089 -1.622257
H22 1.080543 6.801401 -2.416704
H23 -2.256052 5.263977 -3.031086
H24 -0.875885 6.019651 -3.853592
C33 0.585593 3.789117 -4.168829
H25 0.786409 4.647372 -4.852876
C34 0.399738 2.524708 -4.963437
H26 0.355763 2.770200 -6.050002
H27 -2.475502 0.630530 -4.467129
H28 -1.613932 0.739495 -6.003542
C35 0.255689 -0.973013 -5.525754
H29 -0.118038 -0.796525 -6.557708
C36 0.274906 -2.442189 -5.250662
H30 -0.157766 -2.986134 -6.124427
H31 -2.236925 -3.793116 -3.608226
H32 -1.066602 -4.549916 -4.698626
C37 0.857957 -5.073045 -2.650781
H33 0.943669 -6.044250 -3.190059
C38 1.167203 -5.284090 -1.194041
H34 1.461328 -6.346746 -1.021506
H35 -1.448747 -5.540343 0.963204
H36 0.087435 -6.428605 0.973065
C39 1.555980 -4.574592 2.252995
H37 1.853995 -5.631379 2.447686
C40 1.557680 -3.796798 3.546576
H38 1.845664 -4.472206 4.386307

H39 -1.368589 -2.499058 4.850966
H40 0.154193 -2.743488 5.720023
C41 -0.788150 -0.094087 4.022377
O -1.924808 -0.310728 3.606857
N 2.513970 -0.688709 4.593599
H41 1.206540 -0.677578 6.255764
C42 1.001297 1.051070 4.990722
H42 0.835504 1.534162 5.981890
148
CBpath coordinate 6.20
C1 0.167111 -1.081780 0.200219
C2 1.031717 -1.293226 -0.836874
C3 2.282875 -0.723439 -0.457175
C4 2.094710 -0.205082 0.790082
O 0.818063 -0.423772 1.211590
H1 0.807444 -1.796280 -1.766428
H2 3.201170 -0.725695 -1.027931
C5 -1.269810 -1.358760 0.420553
H3 -1.493798 -1.374904 1.484688
H4 -1.566855 -2.315036 -0.010615
N -2.234237 -0.333979 -0.202794
H5 -3.168252 -0.519446 0.188712
C6 -1.927889 1.143051 0.023411
H6 -1.811869 1.280756 1.099769
H7 -2.830445 1.674636 -0.298117
C7 -0.724944 1.584203 -0.747645
C8 0.288308 2.272142 -0.224906
H8 0.335468 2.520527 0.830993
H9 1.133429 2.572408 -0.833044
H10 -0.712074 1.308261 -1.800706
H11 -2.280104 -0.532582 -1.220490
H12 2.742804 0.296907 1.494668
C1 1.213047 -0.426062 5.168474
N 0.103751 -1.050699 4.483275
C2 -0.261518 -2.423868 4.735613
N 0.184246 -3.347032 3.712340
C3 -0.640415 -3.864549 2.729641
O -1.867160 -3.779670 2.687662
N 0.174256 -4.502319 1.804317
C4 -0.347155 -5.418008 0.817662
N -0.087889 -4.973501 -0.529638
C5 -1.105750 -4.687406 -1.433095
O -2.296463 -4.503677 -1.175878
N -0.525033 -4.618434 -2.687241
C6 -1.139097 -3.904011 -3.787705
N -0.562252 -2.613986 -4.079487
C7 -1.144536 -1.409298 -3.695348
O -1.999464 -1.254289 -2.821722
N -0.653871 -0.420221 -4.541924
C8 -1.448297 0.733149 -4.905914
N -0.879570 2.002363 -4.512333
C9 -1.552095 2.920352 -3.722352
O -2.716767 2.826401 -3.337631
N -0.670791 3.960665 -3.460678
C10 -1.139531 5.273027 -3.071476
N -0.631388 5.707190 -1.791582
C11 -1.432168 6.323019 -0.834498
O -2.655463 6.470507 -0.875987
N -0.596884 6.721393 0.198439
C12 -0.981458 6.554026 1.595654
N -0.408948 5.433389 2.315369

C13 -1.027356 4.197513 2.431878
O -2.055856 3.847398 1.859903
N -0.312078 3.447164 3.365009
C14 -0.956921 2.377981 4.105700
N -0.187720 1.152264 4.163372
H1 -1.953528 2.161051 3.647696
H2 -1.181497 2.722241 5.141633
C15 0.702260 4.254943 4.023033
N 2.088494 3.860805 3.839645
C16 2.706468 2.844759 4.652842
N 2.230401 1.513937 4.372218
C17 3.122531 0.472400 4.148035
O 4.255668 0.563501 3.678602
H3 2.547966 3.087003 5.727252
H4 3.812889 2.869268 4.501271
C18 2.847048 4.897884 3.315401
O 4.073349 4.931392 3.223469
N 1.972147 5.880361 2.887215
C19 2.292390 6.638792 1.700166
N 1.768786 6.083948 0.483539
C20 2.351539 5.054377 -0.234683
O 3.300196 4.360096 0.122879
N 1.733212 5.021749 -1.478144
C21 2.445675 4.556056 -2.644512
N 1.746128 3.506906 -3.340585
C22 2.385263 2.339977 -3.735832
O 3.509995 1.981714 -3.392382
N 1.566188 1.713557 -4.664423
C23 2.100741 0.784755 -5.636173
N 1.647260 -0.569726 -5.430254
C24 2.488961 -1.667051 -5.326614
O 3.715364 -1.668285 -5.403489
N 1.683415 -2.773135 -5.103770
C25 2.169880 -4.005829 -4.522536
N 1.868316 -4.145459 -3.119665
C26 2.796537 -3.876038 -2.123298
O 3.879088 -3.312442 -2.277727
N 2.289367 -4.397974 -0.935396
C27 3.108729 -4.536933 0.252454
N 2.545186 -3.901880 1.429118
C28 3.254590 -2.964788 2.175203
O 4.324564 -2.437181 1.871978
N 2.554317 -2.763520 3.356271
C29 3.080901 -2.009272 4.467970
H5 4.191159 -1.934181 4.384411
H6 2.896514 -2.564018 5.414088
H7 4.121915 -4.108085 0.052097
H8 3.288104 -5.616365 0.456570
H9 3.273003 -4.094248 -4.682358
H10 1.726492 -4.861501 -5.082222
H11 3.219424 0.810822 -5.615366
H12 1.821137 1.111752 -6.663441
H13 3.465622 4.204592 -2.349844
H14 2.619765 5.401662 -3.342659
H15 3.396680 6.777452 1.602263
H16 1.880347 7.663925 1.831599
H17 0.473955 4.374620 5.107195
C30 0.617135 5.586145 3.330172
H18 0.296176 6.358210 4.069142
H19 -2.094981 6.498260 1.673698
H20 -0.707180 7.489319 2.134587

C31 0.772712 6.779043 -0.291880
H21 1.077053 7.844002 -0.438001
C32 0.759628 6.087333 -1.620572
H22 1.080920 6.793722 -2.415297
H23 -2.255872 5.265152 -3.036255
H24 -0.871152 6.018718 -3.853707
C33 0.586377 3.788434 -4.167588
H25 0.787310 4.646949 -4.851374
C34 0.400514 2.524836 -4.962527
H26 0.354515 2.770545 -6.048959
H27 -2.475748 0.632364 -4.475006
H28 -1.606719 0.744823 -6.007627
C35 0.257934 -0.971724 -5.525727
H29 -0.115150 -0.797584 -6.558323
C36 0.278047 -2.440273 -5.247718
H30 -0.152817 -2.986187 -6.121128
H31 -2.236070 -3.787882 -3.604182
H32 -1.067647 -4.545899 -4.695912
C37 0.858698 -5.070232 -2.650256
H33 0.943105 -6.039914 -3.192374
C38 1.167438 -5.285629 -1.194216
H34 1.460397 -6.349082 -1.024566
H35 -1.448258 -5.545351 0.963282
H36 0.089433 -6.431239 0.973217
C39 1.554540 -4.573481 2.253312
H37 1.855410 -5.629196 2.449679
C40 1.556008 -3.793510 3.544690
H38 1.846974 -4.466151 4.385653
H39 -1.370635 -2.498610 4.850132
H40 0.151842 -2.744246 5.719488
C41 -0.789433 -0.093707 4.024871
O -1.926656 -0.309927 3.610398
N 2.514413 -0.688374 4.591278
H41 1.208732 -0.678751 6.254539
C42 1.002433 1.050369 4.990686
H42 0.839387 1.533137 5.982466
148
CBpath coordinate 6.00
C1 0.149504 -1.062064 0.195431
C2 1.019531 -1.281621 -0.837266
C3 2.261466 -0.691095 -0.465529
C4 2.064818 -0.152805 0.772436
O 0.793932 -0.389957 1.200640
H1 0.800834 -1.802668 -1.758497
H2 3.181104 -0.691913 -1.034691
C5 -1.281024 -1.359597 0.419681
H3 -1.503404 -1.378288 1.485060
H4 -1.568093 -2.318259 -0.012028
N -2.245941 -0.337865 -0.201043
H5 -3.181120 -0.513799 0.194026
C6 -1.914524 1.133627 0.020737
H6 -1.809040 1.273942 1.098850
H7 -2.800195 1.682168 -0.316559
C7 -0.693216 1.546650 -0.736726
C8 0.324618 2.223971 -0.206821
H8 0.364969 2.473615 0.849195
H9 1.171521 2.526266 -0.811709
H10 -0.680611 1.280689 -1.792813
H11 -2.295304 -0.537421 -1.218287
H12 2.709279 0.360544 1.471845
C1 1.213781 -0.426190 5.167971

N 0.103884 -1.050934 4.483367
C2 -0.262435 -2.423952 4.735476
N 0.182768 -3.347302 3.712076
C3 -0.641944 -3.865829 2.729788
O -1.868611 -3.781197 2.687649
N 0.172894 -4.504271 1.805006
C4 -0.347058 -5.420264 0.817818
N -0.087656 -4.975447 -0.529500
C5 -1.105405 -4.688273 -1.432649
O -2.296184 -4.505078 -1.175326
N -0.524417 -4.617052 -2.686538
C6 -1.138651 -3.902033 -3.786380
N -0.560804 -2.612528 -4.078054
C7 -1.143796 -1.407779 -3.695444
O -1.999494 -1.252494 -2.822458
N -0.653379 -0.419666 -4.543146
C8 -1.447432 0.733510 -4.907804
N -0.878890 2.001391 -4.510767
C9 -1.550409 2.916975 -3.717162
O -2.714529 2.821876 -3.330613
N -0.670305 3.958793 -3.458113
C10 -1.139931 5.271338 -3.071022
N -0.632794 5.705229 -1.790954
C11 -1.433344 6.322186 -0.834624
O -2.656468 6.470837 -0.876788
N -0.597963 6.719808 0.198531
C12 -0.982800 6.550306 1.595330
N -0.407909 5.430254 2.313781
C13 -1.022812 4.192316 2.427301
O -2.048599 3.839535 1.852128
N -0.309725 3.444865 3.364329
C14 -0.955912 2.377978 4.106772
N -0.187714 1.151980 4.164653
H1 -1.953331 2.161929 3.650155
H2 -1.178664 2.723630 5.142667
C15 0.703275 4.254051 4.022524
N 2.089767 3.860842 3.839941
C16 2.707703 2.844202 4.652094
N 2.230472 1.514022 4.370814
C17 3.122191 0.472703 4.145111
O 4.254594 0.564160 3.673712
H3 2.550119 3.085833 5.726756
H4 3.814033 2.868320 4.499626
C18 2.847810 4.898009 3.315375
O 4.074100 4.931632 3.222761
N 1.972461 5.880071 2.887212
C19 2.292423 6.637503 1.699533
N 1.767946 6.081497 0.483955
C20 2.348951 5.049742 -0.232587
O 3.297449 4.355399 0.125386
N 1.730271 5.016151 -1.475754
C21 2.445115 4.553335 -2.641635
N 1.746487 3.505328 -3.339635
C22 2.385740 2.339032 -3.735785
O 3.510476 1.980487 -3.392333
N 1.566708 1.713639 -4.664944
C23 2.100950 0.785716 -5.637399
N 1.648064 -0.568837 -5.431318
C24 2.490371 -1.665331 -5.325342
O 3.716880 -1.665884 -5.401426
N 1.685346 -2.771496 -5.101553

C25 2.172428 -4.004002 -4.520803
N 1.869227 -4.144224 -3.118445
C26 2.796891 -3.875499 -2.121472
O 3.879459 -3.311605 -2.274543
N 2.289379 -4.399343 -0.934868
C27 3.107202 -4.538136 0.253545
N 2.541067 -3.899261 1.426290
C28 3.246168 -2.955272 2.167458
O 4.312302 -2.422436 1.859972
N 2.549978 -2.758373 3.351607
C29 3.080898 -2.008216 4.463551
H5 4.190992 -1.933966 4.377163
H6 2.898347 -2.564734 5.409003
H7 4.121731 -4.111873 0.054457
H8 3.283746 -5.617597 0.460397
H9 3.275872 -4.091008 -4.679352
H10 1.730637 -4.859831 -5.081448
H11 3.219648 0.812344 -5.617339
H12 1.820405 1.112900 -6.664331
H13 3.464810 4.201608 -2.346412
H14 2.620060 5.400140 -3.338314
H15 3.396680 6.775852 1.600818
H16 1.880598 7.662805 1.830249
H17 0.474505 4.373965 5.106589
C30 0.617309 5.584639 3.329249
H18 0.295026 6.356693 4.067653
H19 -2.096235 6.492025 1.673141
H20 -0.710561 7.485586 2.135280
C31 0.771842 6.776320 -0.291630
H21 1.076571 7.841058 -0.438619
C32 0.758514 6.083434 -1.619475
H22 1.080879 6.789110 -2.414613
H23 -2.256324 5.263801 -3.037302
H24 -0.870063 6.016731 -3.853103
C33 0.586465 3.787409 -4.165954
H25 0.787132 4.646555 -4.849126
C34 0.400564 2.524753 -4.961751
H26 0.353845 2.771274 -6.048010
H27 -2.475837 0.632273 -4.479338
H28 -1.603021 0.746725 -6.009918
C35 0.258901 -0.971754 -5.526183
H29 -0.114265 -0.799166 -6.559007
C36 0.279786 -2.439835 -5.246309
H30 -0.150402 -2.987139 -6.119162
H31 -2.235391 -3.784988 -3.602035
H32 -1.068326 -4.543743 -4.694758
C37 0.859135 -5.069377 -2.650435
H33 0.943020 -6.038248 -3.194033
C38 1.167598 -5.286853 -1.194714
H34 1.460486 -6.350607 -1.026593
H35 -1.448076 -5.548624 0.963101
H36 0.090429 -6.433113 0.973475
C39 1.553559 -4.572218 2.252870
H37 1.856798 -5.627158 2.450063
C40 1.555111 -3.791039 3.542955
H38 1.848974 -4.461850 4.384416
H39 -1.371594 -2.498206 4.849914
H40 0.150682 -2.744660 5.719357
C41 -0.789725 -0.093817 4.025942
O -1.927103 -0.309814 3.612119
N 2.515384 -0.687700 4.590726

H41 1.209924 -0.679018 6.254016
C42 1.003190 1.050132 4.990628
H42 0.841188 1.532786 5.982652
148
CBpath coordinate 5.80
C1 0.134222 -1.052455 0.194623
C2 1.007734 -1.271665 -0.835311
C3 2.237820 -0.652484 -0.472752
C4 2.030182 -0.099904 0.758806
O 0.769486 -0.368558 1.196579
H1 0.795620 -1.803050 -1.751626
H2 3.154169 -0.634399 -1.046340
C5 -1.294240 -1.362659 0.418642
H3 -1.518465 -1.385018 1.482892
H4 -1.577116 -2.320630 -0.017591
N -2.255574 -0.337229 -0.200621
H5 -3.189229 -0.497248 0.203498
C6 -1.895201 1.128732 0.013549
H6 -1.800514 1.274435 1.090914
H7 -2.765726 1.693992 -0.337737
C7 -0.653813 1.506201 -0.730086
C8 0.370893 2.170846 -0.188791
H8 0.397056 2.429462 0.864773
H9 1.225147 2.467023 -0.785323
H10 -0.634444 1.243757 -1.785851
H11 -2.314031 -0.540298 -1.216465
H12 2.663752 0.440725 1.446736
C1 1.214749 -0.426107 5.167410
N 0.104272 -1.051252 4.483386
C2 -0.263103 -2.424076 4.735367
N 0.181065 -3.347774 3.711842
C3 -0.643797 -3.867177 2.729986
O -1.870503 -3.782978 2.687950
N 0.171073 -4.505955 1.805452
C4 -0.347205 -5.422407 0.817755
N -0.087818 -4.977314 -0.529650
C5 -1.105542 -4.689711 -1.432637
O -2.296467 -4.507532 -1.175357
N -0.524204 -4.616402 -2.686289
C6 -1.138614 -3.900892 -3.785571
N -0.559710 -2.611989 -4.077235
C7 -1.144355 -1.407546 -3.696528
O -2.001762 -1.252438 -2.825145
N -0.653476 -0.420144 -4.544644
C8 -1.446879 0.733212 -4.908933
N -0.877844 1.998362 -4.505733
C9 -1.547944 2.910472 -3.706852
O -2.710628 2.813066 -3.316431
N -0.669666 3.954911 -3.452530
C10 -1.141021 5.267704 -3.069302
N -0.634804 5.702844 -1.789699
C11 -1.434892 6.321763 -0.834545
O -2.657835 6.471903 -0.877400
N -0.599241 6.718562 0.198681
C12 -0.984307 6.546254 1.594944
N -0.406666 5.426676 2.311528
C13 -1.017031 4.186131 2.420856
O -2.039028 3.830001 1.841010
N -0.306812 3.441977 3.362496
C14 -0.954813 2.377633 4.106565
N -0.187641 1.151413 4.165404

H1 -1.952775 2.162322 3.650783
H2 -1.176388 2.724928 5.142183
C15 0.704094 4.252919 4.021570
N 2.091031 3.861187 3.840461
C16 2.708817 2.844024 4.651682
N 2.230562 1.514498 4.369485
C17 3.122440 0.473663 4.142816
O 4.254180 0.565922 3.670146
H3 2.551801 3.084940 5.726565
H4 3.815084 2.867965 4.498613
C18 2.848515 4.898633 3.315811
O 4.074722 4.932508 3.222578
N 1.972507 5.880146 2.887612
C19 2.292114 6.636340 1.699139
N 1.766937 6.078704 0.484787
C20 2.345392 5.043699 -0.229091
O 3.293282 4.348802 0.129914
N 1.726534 5.008754 -1.471958
C21 2.444346 4.549041 -2.637019
N 1.747156 3.502372 -3.337608
C22 2.386700 2.336984 -3.735397
O 3.511361 1.977961 -3.392183
N 1.567389 1.713091 -4.665228
C23 2.100796 0.786410 -5.639067
N 1.648502 -0.568263 -5.432785
C24 2.491652 -1.663814 -5.324560
O 3.718194 -1.663487 -5.399735
N 1.687193 -2.770202 -5.100008
C25 2.174872 -4.002264 -4.519315
N 1.869454 -4.142299 -3.117626
C26 2.796004 -3.872672 -2.120015
O 3.877933 -3.307301 -2.271724
N 2.288581 -4.398926 -0.934794
C27 3.105065 -4.537247 0.253824
N 2.535831 -3.894590 1.422198
C28 3.235686 -2.942456 2.157608
O 4.296414 -2.402337 1.844587
N 2.544747 -2.751612 3.345803
C29 3.081011 -2.006602 4.458054
H5 4.190883 -1.933542 4.368060
H6 2.900683 -2.565606 5.402476
H7 4.120838 -4.113360 0.056037
H8 3.278713 -5.616693 0.463544
H9 3.278677 -4.088030 -4.676182
H10 1.734707 -4.858448 -5.080638
H11 3.219527 0.813631 -5.620426
H12 1.818790 1.114160 -6.665437
H13 3.463668 4.197000 -2.340897
H14 2.620212 5.397340 -3.331851
H15 3.396291 6.774664 1.599573
H16 1.880131 7.661709 1.828789
H17 0.474186 4.372833 5.105414
C30 0.617158 5.582914 3.328116
H18 0.292882 6.354755 4.065839
H19 -2.097630 6.485214 1.672722
H20 -0.714117 7.481318 2.136239
C31 0.770878 6.773033 -0.291325
H21 1.076402 7.837308 -0.440012
C32 0.757077 6.078160 -1.617781
H22 1.081050 6.782469 -2.413703
H23 -2.257481 5.259987 -3.037023

H24 -0.869956 6.011931 -3.852188
C33 0.586160 3.784839 -4.162257
H25 0.785878 4.644915 -4.844609
C34 0.399965 2.523444 -4.959118
H26 0.350810 2.771151 -6.045051
H27 -2.476489 0.631327 -4.483586
H28 -1.598803 0.749869 -6.011531
C35 0.259510 -0.972323 -5.526985
H29 -0.113822 -0.801264 -6.559993
C36 0.281381 -2.439985 -5.245387
H30 -0.147949 -2.988639 -6.117773
H31 -2.235113 -3.782934 -3.600301
H32 -1.069465 -4.542397 -4.694122
C37 0.859462 -5.068283 -2.650757
H33 0.943497 -6.036469 -3.195483
C38 1.167651 -5.287152 -1.195357
H34 1.461436 -6.350860 -1.028335
H35 -1.448095 -5.552079 0.962783
H36 0.091510 -6.434718 0.973576
C39 1.552408 -4.569724 2.251437
H37 1.858927 -5.623683 2.448982
C40 1.554217 -3.787690 3.540265
H38 1.851943 -4.456610 4.381881
H39 -1.372295 -2.497730 4.850065
H40 0.150103 -2.745106 5.719081
C41 -0.789639 -0.094223 4.026668
O -1.927278 -0.310252 3.613277
N 2.516895 -0.686514 4.590709
H41 1.211136 -0.679011 6.253436
C42 1.003942 1.050027 4.990340
H42 0.842643 1.532551 5.982553
148
CBpath coordinate 5.61
C1 0.116595 -1.031917 0.188160
C2 0.997398 -1.253942 -0.837952
C3 2.216424 -0.616158 -0.477887
C4 1.997787 -0.052295 0.746916
O 0.745009 -0.344434 1.190388
H1 0.792942 -1.797766 -1.748179
H2 3.133516 -0.589500 -1.050609
C5 -1.305453 -1.362723 0.415003
H3 -1.526467 -1.390657 1.480688
H4 -1.579685 -2.321096 -0.024459
N -2.265435 -0.336327 -0.198980
H5 -3.199791 -0.483611 0.210340
C6 -1.875469 1.122546 0.008269
H6 -1.792042 1.274319 1.086765
H7 -2.725135 1.705771 -0.361978
C7 -0.615178 1.465792 -0.720349
C8 0.410928 2.125398 -0.176308
H8 0.429252 2.393178 0.875887
H9 1.267210 2.422939 -0.769978
H10 -0.594732 1.205605 -1.777676
H11 -2.330194 -0.540576 -1.213918
H12 2.627834 0.492960 1.435305
C1 1.215449 -0.425820 5.167170
N 0.104973 -1.051590 4.483343
C2 -0.263259 -2.424223 4.735474
N 0.180004 -3.348366 3.711921
C3 -0.645147 -3.868330 2.730422
O -1.871840 -3.784923 2.688894

N 0.169747 -4.506629 1.805470
C4 -0.347432 -5.423430 0.817435
N -0.088138 -4.978209 -0.530082
C5 -1.105944 -4.691395 -1.433171
O -2.297086 -4.510469 -1.176000
N -0.524385 -4.616854 -2.686665
C6 -1.138932 -3.901227 -3.785699
N -0.559627 -2.612563 -4.077293
C7 -1.145036 -1.408375 -3.697175
O -2.002920 -1.253357 -2.826147
N -0.654276 -0.421374 -4.545701
C8 -1.446986 0.732386 -4.909234
N -0.877275 1.995056 -4.500615
C9 -1.545797 2.904186 -3.697062
O -2.707201 2.804664 -3.303041
N -0.669253 3.950958 -3.446982
C10 -1.142211 5.263915 -3.066942
N -0.636257 5.700727 -1.788146
C11 -1.435905 6.321678 -0.834192
O -2.658682 6.473070 -0.877689
N -0.600056 6.718164 0.198953
C12 -0.985389 6.543807 1.594801
N -0.405915 5.424516 2.310075
C13 -1.012734 4.181893 2.415616
O -2.031842 3.823553 1.832137
N -0.304675 3.439863 3.360514
C14 -0.954107 2.377021 4.105203
N -0.187326 1.150863 4.165239
H1 -1.952045 2.161970 3.649265
H2 -1.175700 2.725334 5.140506
C15 0.704369 4.252061 4.020790
N 2.091750 3.861664 3.841068
C16 2.709370 2.844343 4.651917
N 2.230796 1.515122 4.369046
C17 3.122927 0.474699 4.142361
O 4.254562 0.567500 3.669354
H3 2.552370 3.084848 5.726881
H4 3.815632 2.868384 4.498755
C18 2.848743 4.899523 3.316661
O 4.074950 4.933832 3.223323
N 1.972257 5.880550 2.888335
C19 2.291636 6.635779 1.699227
N 1.766209 6.076985 0.485620
C20 2.342574 5.039231 -0.225993
O 3.289577 4.343596 0.134156
N 1.724014 5.003459 -1.468906
C21 2.444014 4.545228 -2.633110
N 1.747695 3.499615 -3.335654
C22 2.387208 2.334817 -3.734673
O 3.511918 1.975379 -3.391847
N 1.567478 1.711948 -4.664736
C23 2.099883 0.786450 -5.639980
N 1.648030 -0.568342 -5.433880
C24 2.491756 -1.663206 -5.324566
O 3.718394 -1.662113 -5.399069
N 1.687831 -2.769884 -5.099922
C25 2.176064 -4.001358 -4.518855
N 1.869020 -4.140797 -3.117611
C26 2.794436 -3.869481 -2.119568
O 3.875612 -3.302500 -2.270474
N 2.287447 -4.397336 -0.935162

C27 3.103505 -4.535023 0.253333
N 2.531869 -3.890238 1.418803
C28 3.227883 -2.932613 2.150430
O 4.284812 -2.387321 1.833601
N 2.540879 -2.746315 3.341547
C29 3.081197 -2.005204 4.454082
H5 4.190884 -1.932949 4.361250
H6 2.902837 -2.566226 5.397673
H7 4.119766 -4.111958 0.056229
H8 3.276235 -5.614330 0.464886
H9 3.280152 -4.086120 -4.674471
H10 1.737214 -4.858008 -5.080441
H11 3.218638 0.814111 -5.622677
H12 1.816476 1.114896 -6.665742
H13 3.462746 4.192456 -2.335848
H14 2.621389 5.394270 -3.326789
H15 3.395747 6.774199 1.599162
H16 1.879459 7.661170 1.828049
H17 0.473306 4.371586 5.104455
C30 0.616759 5.581838 3.327599
H18 0.291016 6.353319 4.065050
H19 -2.098626 6.480714 1.672394
H20 -0.716795 7.478702 2.137145
C31 0.770268 6.770867 -0.291041
H21 1.076472 7.834741 -0.441192
C32 0.756031 6.074185 -1.616337
H22 1.080879 6.777245 -2.413145
H23 -2.258706 5.255574 -3.035124
H24 -0.870994 6.007120 -3.850814
C33 0.585612 3.782142 -4.158637
H25 0.784213 4.642950 -4.840447
C34 0.399039 2.521688 -4.956220
H26 0.347780 2.770205 -6.041906
H27 -2.477551 0.629947 -4.486391
H28 -1.596010 0.752202 -6.012203
C35 0.259171 -0.973251 -5.527759
H29 -0.114255 -0.802923 -6.560836
C36 0.281772 -2.440654 -5.245401
H30 -0.147007 -2.989999 -6.117591
H31 -2.235336 -3.782951 -3.600030
H32 -1.070231 -4.542640 -4.694321
C37 0.859564 -5.067732 -2.651251
H33 0.944197 -6.035583 -3.196427
C38 1.167594 -5.286803 -1.195969
H34 1.462338 -6.350338 -1.029316
H35 -1.448220 -5.554030 0.962337
H36 0.092101 -6.435399 0.973344
C39 1.551620 -4.567407 2.249885
H37 1.860657 -5.620694 2.447303
C40 1.553743 -3.785162 3.538097
H38 1.854587 -4.452962 4.379519
H39 -1.372436 -2.497417 4.850603
H40 0.150164 -2.745370 5.719067
C41 -0.789273 -0.094846 4.026613
O -1.926795 -0.311166 3.613333
N 2.518232 -0.685390 4.591292
H41 1.211787 -0.678533 6.253243
C42 1.004310 1.050145 4.990015
H42 0.842920 1.532698 5.982217
148
CBpath coordinate 5.41

C1 0.101962 -1.014499 0.181653
C2 0.990871 -1.238449 -0.839203
C3 2.198303 -0.581957 -0.480492
C4 1.965311 -0.005755 0.737525
O 0.723017 -0.328063 1.187735
H1 0.793523 -1.786119 -1.748302
H2 3.113993 -0.538822 -1.054434
C5 -1.316087 -1.362581 0.408905
H3 -1.536129 -1.397028 1.474630
H4 -1.584757 -2.319600 -0.036616
N -2.274676 -0.332871 -0.198240
H5 -3.206985 -0.465830 0.220889
C6 -1.854277 1.118133 0.002391
H6 -1.782113 1.276531 1.080646
H7 -2.684346 1.718435 -0.384602
C7 -0.575400 1.423531 -0.710818
C8 0.455174 2.076513 -0.163138
H8 0.463866 2.360644 0.884974
H9 1.311283 2.376712 -0.755634
H10 -0.553859 1.168125 -1.769534
H11 -2.350264 -0.538154 -1.211910
H12 2.587978 0.552183 1.422412
C1 1.216055 -0.425406 5.167081
N 0.105736 -1.051972 4.483246
C2 -0.263190 -2.424389 4.735676
N 0.179007 -3.349018 3.712123
C3 -0.646385 -3.868945 2.730717
O -1.873189 -3.786209 2.689780
N 0.168376 -4.506576 1.805165
C4 -0.347867 -5.423849 0.817009
N -0.088813 -4.978702 -0.530702
C5 -1.106752 -4.693282 -1.434058
O -2.298165 -4.513764 -1.177168
N -0.524954 -4.618042 -2.687443
C6 -1.139522 -3.902420 -3.786376
N -0.559993 -2.613933 -4.077878
C7 -1.146737 -1.410275 -3.698446
O -2.005829 -1.255778 -2.828493
N -0.655557 -0.423293 -4.546617
C8 -1.447471 0.731126 -4.908666
N -0.876675 1.990792 -4.493845
C9 -1.543407 2.896666 -3.685063
O -2.703048 2.794559 -3.286480
N -0.668780 3.946144 -3.440033
C10 -1.143691 5.259288 -3.063824
N -0.637786 5.698646 -1.786225
C11 -1.436907 6.321937 -0.833598
O -2.659496 6.474578 -0.877647
N -0.600750 6.718199 0.199355
C12 -0.986337 6.541722 1.594766
N -0.405194 5.422504 2.308491
C13 -1.008272 4.177746 2.409816
O -2.024280 3.817185 1.822310
N -0.302476 3.437609 3.357800
C14 -0.953490 2.376148 4.102782
N -0.187008 1.150130 4.164512
H1 -1.951102 2.161113 3.646120
H2 -1.175778 2.725500 5.137603
C15 0.704394 4.251068 4.019781
N 2.092297 3.862219 3.841788
C16 2.709654 2.844866 4.652509

N 2.230923 1.515892 4.368917
C17 3.123698 0.475974 4.142668
O 4.255282 0.569533 3.669915
H3 2.552339 3.085002 5.727500
H4 3.815942 2.869137 4.499567
C18 2.848845 4.900628 3.317867
O 4.075017 4.935467 3.224618
N 1.971824 5.881111 2.889321
C19 2.291073 6.635395 1.699601
N 1.765591 6.075424 0.486641
C20 2.339650 5.034652 -0.222461
O 3.285604 4.338116 0.139009
N 1.721411 4.997922 -1.465423
C21 2.443661 4.541028 -2.628691
N 1.748343 3.496428 -3.333279
C22 2.387854 2.332254 -3.733680
O 3.512619 1.972426 -3.391384
N 1.567489 1.710359 -4.663826
C23 2.098639 0.786078 -5.640699
N 1.647039 -0.568841 -5.435023
C24 2.491345 -1.663131 -5.325188
O 3.718008 -1.661256 -5.399005
N 1.687881 -2.770201 -5.100890
C25 2.176643 -4.000814 -4.518829
N 1.867956 -4.139040 -3.118009
C26 2.792088 -3.865098 -2.119648
O 3.872209 -3.296082 -2.270046
N 2.285787 -4.394390 -0.935832
C27 3.101817 -4.531258 0.252248
N 2.527938 -3.885006 1.415239
C28 3.220080 -2.922019 2.143334
O 4.272825 -2.371091 1.822696
N 2.537109 -2.740845 3.337544
C29 3.081364 -2.003693 4.450444
H5 4.190865 -1.932348 4.354894
H6 2.904780 -2.566911 5.393055
H7 4.118261 -4.108416 0.055624
H8 3.274100 -5.610420 0.465224
H9 3.280994 -4.084787 -4.673149
H10 1.738990 -4.858170 -5.080232
H11 3.217422 0.813980 -5.624850
H12 1.813819 1.115481 -6.665776
H13 3.461781 4.187525 -2.330221
H14 2.622571 5.390811 -3.321211
H15 3.395116 6.774029 1.599194
H16 1.878581 7.660770 1.827536
H17 0.471774 4.369944 5.103208
C30 0.616162 5.580755 3.327123
H18 0.288773 6.351718 4.064373
H19 -2.099492 6.476851 1.672328
H20 -0.719029 7.476311 2.138236
C31 0.769792 6.768818 -0.290671
H21 1.076843 7.832207 -0.442554
C32 0.754989 6.070055 -1.614648
H22 1.080800 6.771690 -2.412458
H23 -2.260218 5.250004 -3.032219
H24 -0.872714 6.001060 -3.849209
C33 0.584802 3.778762 -4.154134
H25 0.781861 4.640327 -4.835477
C34 0.397730 2.519227 -4.952353
H26 0.343694 2.768549 -6.037761

H27 -2.478930 0.628116 -4.488209
H28 -1.593715 0.754796 -6.011962
C35 0.258295 -0.974498 -5.528640
H29 -0.115307 -0.804543 -6.561696
C36 0.281579 -2.441766 -5.245997
H30 -0.146919 -2.991476 -6.118062
H31 -2.235890 -3.784016 -3.600540
H32 -1.070926 -4.543714 -4.695052
C37 0.859507 -5.067162 -2.651743
H33 0.945292 -6.034900 -3.196873
C38 1.167409 -5.285645 -1.196516
H34 1.463719 -6.348744 -1.029709
H35 -1.448551 -5.555295 0.961922
H36 0.092468 -6.435458 0.973109
C39 1.550877 -4.564476 2.247875
H37 1.862473 -5.617146 2.444734
C40 1.553389 -3.782514 3.535736
H38 1.857442 -4.449520 4.376650
H39 -1.372345 -2.497135 4.851410
H40 0.150664 -2.745573 5.719061
C41 -0.788482 -0.095712 4.025741
O -1.925729 -0.312572 3.611669
N 2.519651 -0.684125 4.592522
H41 1.212010 -0.677845 6.253215
C42 1.004469 1.050324 4.989613
H42 0.842588 1.532951 5.981719
148
CBpath coordinate 5.21
C1 0.089747 -0.997480 0.173402
C2 0.988673 -1.223959 -0.838412
C3 2.183527 -0.548420 -0.479745
C4 1.931063 0.041819 0.731309
O 0.701267 -0.316546 1.188089
H1 0.800127 -1.773210 -1.748725
H2 3.095633 -0.481959 -1.055120
C5 -1.325709 -1.362644 0.400219
H3 -1.544936 -1.409195 1.465129
H4 -1.590524 -2.315674 -0.056516
N -2.281836 -0.325710 -0.196595
H5 -3.210367 -0.441976 0.234735
C6 -1.829061 1.116843 -0.004380
H6 -1.767633 1.285633 1.071923
H7 -2.639672 1.733212 -0.408586
C7 -0.533760 1.379962 -0.704082
C8 0.502750 2.029920 -0.153769
H8 0.499255 2.337667 0.887478
H9 1.353622 2.339963 -0.747590
H10 -0.512533 1.129830 -1.763187
H11 -2.370401 -0.531696 -1.208576
H12 2.545809 0.607813 1.416184
C1 1.216591 -0.424672 5.167218
N 0.106949 -1.052540 4.483084
C2 -0.262896 -2.424604 4.736126
N 0.177897 -3.350072 3.712760
C3 -0.647974 -3.869578 2.731307
O -1.874807 -3.787813 2.691334
N 0.166656 -4.505679 1.804475
C4 -0.348673 -5.423504 0.816244
N -0.089863 -4.978703 -0.531761
C5 -1.108090 -4.696187 -1.435728
O -2.299965 -4.518967 -1.179385

N -0.525980 -4.620518 -2.688969
C6 -1.140629 -3.905189 -3.787982
N -0.561331 -2.616623 -4.079218
C7 -1.149232 -1.413654 -3.699819
O -2.009266 -1.259807 -2.830545
N -0.657936 -0.426425 -4.547400
C8 -1.448709 0.729094 -4.907117
N -0.876220 1.984795 -4.484346
C9 -1.540512 2.886768 -3.669409
O -2.697775 2.781177 -3.264519
N -0.668369 3.939631 -3.430656
C10 -1.145781 5.252870 -3.059098
N -0.639520 5.695866 -1.783307
C11 -1.437907 6.322519 -0.832548
O -2.660260 6.476810 -0.877323
N -0.601372 6.718731 0.200025
C12 -0.987295 6.539807 1.594922
N -0.404378 5.420606 2.306741
C13 -1.003208 4.173435 2.402749
O -2.015478 3.810717 1.810244
N -0.299965 3.434966 3.353792
C14 -0.952924 2.374654 4.098428
N -0.186378 1.149054 4.162619
H1 -1.949701 2.159312 3.640120
H2 -1.176945 2.725011 5.132545
C15 0.704096 4.249690 4.018412
N 2.092750 3.862921 3.842764
C16 2.709680 2.845777 4.653736
N 2.231213 1.516952 4.369283
C17 3.124848 0.477700 4.144443
O 4.256778 0.572156 3.672451
H3 2.551517 3.085596 5.728654
H4 3.816064 2.870517 4.501468
C18 2.848740 4.902132 3.319809
O 4.074947 4.937807 3.226986
N 1.971143 5.881963 2.890917
C19 2.290312 6.635127 1.700486
N 1.764982 6.073906 0.488126
C20 2.336290 5.029456 -0.217841
O 3.280438 4.331388 0.145576
N 1.718785 4.991838 -1.461068
C21 2.443466 4.535675 -2.623090
N 1.749113 3.492231 -3.329933
C22 2.388485 2.328653 -3.731996
O 3.513420 1.968399 -3.390537
N 1.567146 1.707806 -4.661911
C23 2.096495 0.785075 -5.641008
N 1.644997 -0.569957 -5.436084
C24 2.489844 -1.663683 -5.326499
O 3.716593 -1.660890 -5.399749
N 1.686999 -2.771307 -5.103043
C25 2.176495 -4.000727 -4.519554
N 1.866193 -4.137246 -3.119167
C26 2.788715 -3.859397 -2.120641
O 3.867218 -3.287270 -2.270746
N 2.283594 -4.390052 -0.937216
C27 3.099858 -4.525019 0.250331
N 2.523218 -3.877667 1.410739
C28 3.210998 -2.909250 2.135433
O 4.259087 -2.351823 1.810740
N 2.532688 -2.734134 3.333104

C29 3.081492 -2.001799 4.446563
H5 4.190788 -1.931576 4.347791
H6 2.907027 -2.567847 5.387836
H7 4.116001 -4.101303 0.053978
H8 3.272861 -5.603791 0.464920
H9 3.281136 -4.083774 -4.672491
H10 1.740193 -4.859068 -5.080435
H11 3.215314 0.813080 -5.627016
H12 1.809918 1.115881 -6.665153
H13 3.460665 4.180972 -2.322874
H14 2.624583 5.386051 -3.314430
H15 3.394285 6.774037 1.599805
H16 1.877395 7.660463 1.827318
H17 0.469243 4.367365 5.101501
C30 0.615260 5.579537 3.326833
H18 0.285858 6.349636 4.064058
H19 -2.100366 6.472952 1.672437
H20 -0.721386 7.473953 2.139781
C31 0.769428 6.766659 -0.290093
H21 1.077628 7.829381 -0.444258
C32 0.753825 6.065227 -1.612430
H22 1.080427 6.764980 -2.411670
H23 -2.262348 5.242036 -3.027175
H24 -0.875757 5.992770 -3.846643
C33 0.583538 3.774020 -4.147982
H25 0.778432 4.636424 -4.828910
C34 0.395707 2.515460 -4.946746
H26 0.337924 2.765535 -6.031810
H27 -2.481250 0.625443 -4.489496
H28 -1.591658 0.758015 -6.010750
C35 0.256366 -0.976392 -5.529659
H29 -0.117396 -0.806331 -6.562608
C36 0.280379 -2.443617 -5.247359
H30 -0.147908 -2.993304 -6.119503
H31 -2.237082 -3.787186 -3.602300
H32 -1.071573 -4.546372 -4.696674
C37 0.859290 -5.066887 -2.652531
H33 0.946922 -6.034737 -3.197070
C38 1.167076 -5.283750 -1.197227
H34 1.465483 -6.346145 -1.029594
H35 -1.449242 -5.555787 0.961203
H36 0.092486 -6.434719 0.972670
C39 1.549926 -4.560298 2.244903
H37 1.864640 -5.612287 2.440520
C40 1.553017 -3.779294 3.532771
H38 1.861102 -4.445756 4.372651
H39 -1.371998 -2.496674 4.852797
H40 0.151638 -2.745678 5.719255
C41 -0.787051 -0.097164 4.023238
O -1.923286 -0.314994 3.607117
N 2.521572 -0.682439 4.594983
H41 1.211667 -0.676475 6.253484
C42 1.004341 1.050682 4.988896
H42 0.841188 1.533606 5.980661
148
CBpath coordinate 5.02
C1 0.078031 -0.967878 0.162259
C2 0.993000 -1.207011 -0.837230
C3 2.175193 -0.521083 -0.474438
C4 1.901322 0.090378 0.726120
O 0.683608 -0.300515 1.188289

H1 0.811556 -1.758977 -1.747759
H2 3.089212 -0.443025 -1.046004
C5 -1.331588 -1.357619 0.388164
H3 -1.547861 -1.419741 1.452708
H4 -1.586668 -2.307325 -0.081046
N -2.291839 -0.317522 -0.194577
H5 -3.215119 -0.420941 0.251169
C6 -1.807815 1.116318 -0.010773
H6 -1.755321 1.293746 1.064704
H7 -2.597412 1.749388 -0.430930
C7 -0.497233 1.334820 -0.696345
C8 0.543950 1.987378 -0.150283
H8 0.530054 2.326472 0.881194
H9 1.385050 2.311996 -0.751648
H10 -0.478838 1.085243 -1.756498
H11 -2.395830 -0.523230 -1.204622
H12 2.514745 0.651957 1.416250
C1 1.216822 -0.423789 5.167713
N 0.108459 -1.053204 4.482635
C2 -0.262305 -2.424821 4.736656
N 0.177004 -3.351416 3.713702
C3 -0.649329 -3.869721 2.731851
O -1.876247 -3.788835 2.692977
N 0.165039 -4.503808 1.803306
C4 -0.349683 -5.422321 0.815309
N -0.091024 -4.978251 -0.533071
C5 -1.109574 -4.699398 -1.437811
O -2.301893 -4.524318 -1.182040
N -0.527268 -4.624175 -2.691013
C6 -1.141783 -3.908934 -3.790083
N -0.562845 -2.620123 -4.080605
C7 -1.151410 -1.417915 -3.700549
O -2.012126 -1.264855 -2.831535
N -0.660467 -0.430144 -4.547396
C8 -1.450344 0.726459 -4.904444
N -0.876117 1.978497 -4.474654
C9 -1.537827 2.876809 -3.653699
O -2.692686 2.767756 -3.242603
N -0.668135 3.932884 -3.420966
C10 -1.148036 5.246190 -3.053705
N -0.640965 5.693334 -1.780027
C11 -1.438595 6.323397 -0.831143
O -2.660726 6.479076 -0.876488
N -0.601647 6.719964 0.200910
C12 -0.987901 6.539167 1.595379
N -0.403914 5.419754 2.305614
C13 -0.999239 4.170630 2.396546
O -2.008461 3.806588 1.799727
N -0.298015 3.432807 3.349521
C14 -0.952631 2.372744 4.092837
N -0.185488 1.147850 4.159703
H1 -1.948031 2.156609 3.631939
H2 -1.179548 2.723499 5.126197
C15 0.703389 4.248369 4.017131
N 2.092797 3.863574 3.843805
C16 2.709309 2.846970 4.655497
N 2.231598 1.518031 4.370477
C17 3.126237 0.479364 4.147761
O 4.258681 0.574558 3.676867
H3 2.549932 3.086765 5.730236
H4 3.815824 2.872340 4.504242

C18 2.848317 4.903736 3.322124
O 4.074594 4.940345 3.230139
N 1.970279 5.882972 2.892858
C19 2.289511 6.635281 1.701862
N 1.764634 6.073163 0.489739
C20 2.333429 5.025335 -0.213316
O 3.275790 4.325808 0.152037
N 1.716699 4.986793 -1.456881
C21 2.443356 4.530503 -2.617679
N 1.749719 3.488022 -3.326468
C22 2.388948 2.325022 -3.730190
O 3.514245 1.964640 -3.390018
N 1.566395 1.704761 -4.659455
C23 2.093900 0.783513 -5.640765
N 1.642382 -0.571702 -5.437128
C24 2.487636 -1.665118 -5.328546
O 3.714420 -1.661519 -5.401366
N 1.685297 -2.773355 -5.106378
C25 2.175436 -4.001638 -4.521317
N 1.864137 -4.136437 -3.121154
C26 2.785199 -3.854263 -2.122716
O 3.862117 -3.279220 -2.272968
N 2.281275 -4.385238 -0.939148
C27 3.098180 -4.517495 0.247845
N 2.518927 -3.869808 1.406315
C28 3.202884 -2.897182 2.128741
O 4.246956 -2.333949 1.800833
N 2.528728 -2.727864 3.329478
C29 3.081490 -2.000128 4.443750
H5 4.190622 -1.931048 4.342244
H6 2.908849 -2.569110 5.383552
H7 4.113478 -4.091775 0.051389
H8 3.273322 -5.595695 0.463793
H9 3.280268 -4.084030 -4.673344
H10 1.740103 -4.860950 -5.081416
H11 3.212737 0.811439 -5.628459
H12 1.805761 1.115970 -6.663943
H13 3.459529 4.174389 -2.315644
H14 2.626931 5.381081 -3.308227
H15 3.393433 6.774500 1.601192
H16 1.876202 7.660571 1.827801
H17 0.466217 4.364476 5.099904
C30 0.614207 5.578716 3.327049
H18 0.283186 6.347799 4.064586
H19 -2.100913 6.470914 1.672800
H20 -0.722978 7.472880 2.141424
C31 0.769334 6.765269 -0.289429
H21 1.078630 7.827344 -0.445857
C32 0.752889 6.061126 -1.610190
H22 1.079974 6.758982 -2.410967
H23 -2.264609 5.233462 -3.020752
H24 -0.879739 5.984214 -3.843651
C33 0.582015 3.769079 -4.141620
H25 0.774579 4.632244 -4.822253
C34 0.393411 2.511302 -4.940774
H26 0.332092 2.762014 -6.025527
H27 -2.483642 0.622168 -4.488866
H28 -1.590953 0.760205 -6.008279
C35 0.253834 -0.978655 -5.530457
H29 -0.120369 -0.807957 -6.563113
C36 0.278417 -2.445943 -5.249152

H30 -0.150281 -2.995106 -6.121389
H31 -2.238412 -3.791532 -3.604940
H32 -1.071866 -4.549824 -4.698894
C37 0.858938 -5.067444 -2.653491
H33 0.948605 -6.035744 -3.196793
C38 1.166695 -5.281695 -1.197939
H34 1.467265 -6.343252 -1.028818
H35 -1.450185 -5.555065 0.960369
H36 0.091946 -6.433261 0.972280
C39 1.549074 -4.555773 2.241501
H37 1.866590 -5.607277 2.435329
C40 1.552828 -3.776412 3.529872
H38 1.864813 -4.442914 4.368299
H39 -1.371347 -2.496117 4.854386
H40 0.152956 -2.745556 5.719590
C41 -0.784906 -0.098935 4.019320
O -1.919764 -0.318013 3.600050
N 2.523553 -0.680812 4.598745
H41 1.210274 -0.674712 6.254166
C42 1.003859 1.051143 4.988101
H42 0.838620 1.534564 5.979310
148
CBpath coordinate 4.82
C1 0.066781 -0.933023 0.147620
C2 0.999279 -1.194497 -0.836425
C3 2.170077 -0.502369 -0.471221
C4 1.875355 0.138058 0.716442
O 0.670412 -0.287844 1.188946
H1 0.825118 -1.752308 -1.745508
H2 3.087696 -0.421292 -1.036754
C5 -1.335323 -1.351617 0.377631
H3 -1.544796 -1.427249 1.443402
H4 -1.580577 -2.298786 -0.100517
N -2.302688 -0.311857 -0.189656
H5 -3.221643 -0.407746 0.267970
C6 -1.793123 1.114462 -0.014118
H6 -1.747568 1.301767 1.060661
H7 -2.564592 1.759378 -0.448927
C7 -0.470123 1.287983 -0.687207
C8 0.580420 1.945644 -0.145476
H8 0.550358 2.323925 0.872603
H9 1.400790 2.295390 -0.760089
H10 -0.459005 1.053499 -1.750040
H11 -2.418524 -0.516043 -1.198429
H12 2.496033 0.678422 1.416465
C1 1.216755 -0.423101 5.168142
N 0.109433 -1.053649 4.482244
C2 -0.261882 -2.424916 4.737021
N 0.176426 -3.352282 3.714376
C3 -0.650168 -3.869270 2.731995
O -1.877164 -3.788806 2.693810
N 0.163966 -4.501852 1.802201
C4 -0.350479 -5.420969 0.814616
N -0.091752 -4.977533 -0.533992
C5 -1.110524 -4.701259 -1.439282
O -2.303054 -4.527216 -1.183837
N -0.528188 -4.627113 -2.692543
C6 -1.142567 -3.911890 -3.791662
N -0.563908 -2.622874 -4.081467
C7 -1.153044 -1.421214 -3.700517
O -2.014297 -1.268988 -2.831938

N -0.662188 -0.432894 -4.546781
C8 -1.451599 0.724418 -4.901834
N -0.876072 1.974155 -4.467821
C9 -1.536083 2.870164 -3.643079
O -2.689274 2.759100 -3.227993
N -0.667901 3.928345 -3.414246
C10 -1.149551 5.241671 -3.049849
N -0.641859 5.691730 -1.777652
C11 -1.438921 6.324122 -0.829989
O -2.660940 6.480588 -0.875660
N -0.601681 6.721074 0.201589
C12 -0.988019 6.539450 1.595888
N -0.403750 5.419755 2.305167
C13 -0.997060 4.169479 2.392696
O -2.004436 3.805044 1.793063
N -0.297051 3.431646 3.346478
C14 -0.952651 2.371365 4.088560
N -0.184936 1.147032 4.157320
H1 -1.946909 2.154513 3.625550
H2 -1.181951 2.722177 5.121377
C15 0.702721 4.247481 4.016292
N 2.092597 3.863952 3.844478
C16 2.708843 2.847816 4.656848
N 2.231790 1.518725 4.371529
C17 3.127286 0.480417 4.150553
O 4.260119 0.575984 3.680872
H3 2.548444 3.087678 5.731417
H4 3.815447 2.873680 4.506447
C18 2.847924 4.904743 3.323789
O 4.074209 4.942025 3.232494
N 1.969623 5.883695 2.894293
C19 2.288958 6.635465 1.702966
N 1.764451 6.072814 0.490902
C20 2.331640 5.022871 -0.210343
O 3.273057 4.322624 0.156175
N 1.715281 4.983531 -1.454028
C21 2.443196 4.527217 -2.614138
N 1.750089 3.485301 -3.324161
C22 2.389324 2.322723 -3.729081
O 3.514985 1.962516 -3.389923
N 1.565897 1.702643 -4.657672
C23 2.092146 0.782187 -5.640339
N 1.640515 -0.573125 -5.437472
C24 2.485944 -1.666486 -5.330105
O 3.712718 -1.662501 -5.402963
N 1.683845 -2.775120 -5.108906
C25 2.174266 -4.002819 -4.522996
N 1.862756 -4.136589 -3.122885
C26 2.783006 -3.851321 -2.124689
O 3.858807 -3.274366 -2.275327
N 2.279833 -4.381923 -0.940735
C27 3.097246 -4.511852 0.245969
N 2.516218 -3.864204 1.403362
C28 3.197912 -2.889202 2.124775
O 4.239446 -2.322416 1.795185
N 2.526281 -2.723907 3.327519
C29 3.081337 -1.999126 4.442475
H5 4.190382 -1.930871 4.339554
H6 2.909563 -2.570067 5.381241
H7 4.111739 -4.084325 0.049283
H8 3.274387 -5.589585 0.462799

H9 3.279150 -4.085067 -4.674769
H10 1.739158 -4.862632 -5.082474
H11 3.210998 0.809943 -5.629056
H12 1.803121 1.115672 -6.662935
H13 3.458783 4.170338 -2.311015
H14 2.628249 5.377916 -3.304211
H15 3.392852 6.774925 1.602384
H16 1.875385 7.660717 1.828373
H17 0.463961 4.362350 5.098856
C30 0.613460 5.578328 3.327452
H18 0.281552 6.346655 4.065361
H19 -2.101009 6.470802 1.673385
H20 -0.723235 7.472841 2.142529
C31 0.769403 6.764585 -0.288918
H21 1.079488 7.826210 -0.446830
C32 0.752309 6.058588 -1.608617
H22 1.079669 6.755169 -2.410438
H23 -2.266123 5.227636 -3.016051
H24 -0.882617 5.978423 -3.841483
C33 0.580924 3.765699 -4.137332
H25 0.771823 4.629323 -4.817876
C34 0.391787 2.508323 -4.936637
H26 0.328005 2.759335 -6.021191
H27 -2.485200 0.619788 -4.487140
H28 -1.591184 0.761188 -6.005735
C35 0.251956 -0.980232 -5.530585
H29 -0.122606 -0.808826 -6.562992
C36 0.276824 -2.447650 -5.250329
H30 -0.152336 -2.996191 -6.122729
H31 -2.239349 -3.795033 -3.607058
H32 -1.071881 -4.552498 -4.700614
C37 0.858657 -5.068268 -2.654142
H33 0.949692 -6.037083 -3.196225
C38 1.166461 -5.280232 -1.198346
H34 1.468398 -6.341184 -1.027837
H35 -1.450965 -5.553853 0.959734
H36 0.091316 -6.431766 0.972126
C39 1.548488 -4.552433 2.238963
H37 1.867646 -5.603728 2.431331
C40 1.552699 -3.774557 3.527925
H38 1.867085 -4.441444 4.365173
H39 -1.370876 -2.495714 4.855520
H40 0.153932 -2.745381 5.719810
C41 -0.783267 -0.100187 4.016105
O -1.917074 -0.320308 3.594259
N 2.524724 -0.679809 4.601746
H41 1.208817 -0.673308 6.254753
C42 1.003330 1.051515 4.987470
H42 0.836407 1.535325 5.978226
148
CBpath coordinate 4.62
C1 0.058246 -0.901773 0.134550
C2 1.013152 -1.184548 -0.833637
C3 2.167472 -0.480919 -0.465517
C4 1.846605 0.187816 0.709736
O 0.659973 -0.282375 1.194504
H1 0.849582 -1.744573 -1.742941
H2 3.085282 -0.385155 -1.028146
C5 -1.339194 -1.346324 0.362014
H3 -1.542253 -1.443220 1.427525
H4 -1.575668 -2.285668 -0.134705

N -2.316897 -0.300435 -0.180087
H5 -3.224349 -0.381812 0.305228
C6 -1.773604 1.117403 -0.021411
H6 -1.736170 1.317665 1.051395
H7 -2.518928 1.777474 -0.476913
C7 -0.439519 1.237446 -0.680554
C8 0.624735 1.906296 -0.144958
H8 0.579259 2.329281 0.853908
H9 1.419732 2.278197 -0.778048
H10 -0.429228 1.005229 -1.742929
H11 -2.465336 -0.504585 -1.183805
H12 2.470151 0.719211 1.415874
C1 1.216354 -0.421983 5.168758
N 0.110484 -1.054229 4.481799
C2 -0.261417 -2.425045 4.737642
N 0.175480 -3.353569 3.715513
C3 -0.651499 -3.868184 2.732126
O -1.878580 -3.788144 2.694872
N 0.162287 -4.498470 1.800413
C4 -0.351760 -5.418497 0.813547
N -0.092773 -4.976225 -0.535402
C5 -1.111847 -4.704009 -1.441555
O -2.304699 -4.531314 -1.186696
N -0.529494 -4.631896 -2.694922
C6 -1.143583 -3.916499 -3.793975
N -0.565294 -2.627142 -4.082336
C7 -1.155525 -1.426421 -3.699718
O -2.017774 -1.275766 -2.832285
N -0.664563 -0.437078 -4.544991
C8 -1.453482 0.721195 -4.896935
N -0.875890 1.967447 -4.456949
C9 -1.533320 2.860311 -3.626716
O -2.683884 2.746196 -3.205322
N -0.667437 3.921584 -3.404037
C10 -1.151667 5.234972 -3.043967
N -0.643181 5.689387 -1.773941
C11 -1.439374 6.325221 -0.828105
O -2.661250 6.482747 -0.874133
N -0.601622 6.722882 0.202711
C12 -0.987954 6.540366 1.596794
N -0.403540 5.420096 2.304725
C13 -0.993593 4.168109 2.386699
O -1.997918 3.803021 1.782287
N -0.295648 3.430028 3.341721
C14 -0.952853 2.369386 4.081777
N -0.184381 1.145832 4.153515
H1 -1.945313 2.151498 3.615414
H2 -1.185914 2.720217 5.113752
C15 0.701679 4.246147 4.014996
N 2.092228 3.864454 3.845451
C16 2.708020 2.849000 4.658808
N 2.231862 1.519740 4.372947
C17 3.128747 0.481920 4.154835
O 4.262234 0.578080 3.687312
H3 2.546042 3.088912 5.733132
H4 3.814776 2.875571 4.509771
C18 2.847359 4.906246 3.326330
O 4.073613 4.944431 3.236077
N 1.968692 5.884797 2.896562
C19 2.288238 6.635688 1.704671
N 1.764196 6.072265 0.492741

C20 2.329300 5.019374 -0.205925
O 3.269239 4.318130 0.162160
N 1.713243 4.978882 -1.449781
C21 2.442902 4.522589 -2.608904
N 1.750762 3.481447 -3.320901
C22 2.390105 2.319562 -3.727465
O 3.516283 1.959705 -3.389818
N 1.565253 1.699472 -4.654866
C23 2.089623 0.780006 -5.639352
N 1.637740 -0.575358 -5.437320
C24 2.483325 -1.668794 -5.332313
O 3.710057 -1.664344 -5.405586
N 1.681510 -2.777964 -5.112666
C25 2.172253 -4.005046 -4.525857
N 1.860938 -4.137598 -3.125735
C26 2.780293 -3.848018 -2.128122
O 3.854686 -3.268741 -2.279676
N 2.278048 -4.377355 -0.943341
C27 3.095994 -4.503029 0.243187
N 2.512112 -3.855431 1.398903
C28 3.190197 -2.876635 2.118829
O 4.227396 -2.303857 1.786586
N 2.522584 -2.718011 3.324836
C29 3.080839 -1.997637 4.440866
H5 4.189769 -1.930727 4.336065
H6 2.910053 -2.571478 5.378039
H7 4.109126 -4.072465 0.046185
H8 3.276461 -5.579929 0.461604
H9 3.277142 -4.087256 -4.677639
H10 1.737114 -4.865402 -5.084433
H11 3.208498 0.807403 -5.629556
H12 1.799318 1.114829 -6.661165
H13 3.457689 4.164710 -2.304313
H14 2.629815 5.373567 -3.298179
H15 3.392092 6.775399 1.604189
H16 1.874359 7.660924 1.829216
H17 0.460491 4.358987 5.097257
C30 0.612411 5.577861 3.328231
H18 0.279317 6.344965 4.066847
H19 -2.100921 6.471506 1.674601
H20 -0.722980 7.473268 2.144148
C31 0.769609 6.763597 -0.288048
H21 1.080996 7.824502 -0.448223
C32 0.751474 6.054853 -1.606147
H22 1.079206 6.749496 -2.409548
H23 -2.268233 5.219051 -3.009102
H24 -0.886574 5.969746 -3.838106
C33 0.579279 3.760649 -4.130936
H25 0.767522 4.624843 -4.811505
C34 0.389306 2.503686 -4.930176
H26 0.321575 2.754930 -6.014472
H27 -2.487411 0.616066 -4.483277
H28 -1.591857 0.762335 -6.000908
C35 0.249119 -0.982513 -5.530091
H29 -0.126059 -0.809764 -6.562078
C36 0.274303 -2.450191 -5.251745
H30 -0.155838 -2.997530 -6.124418
H31 -2.240624 -3.800539 -3.610356
H32 -1.071522 -4.556477 -4.703266
C37 0.858302 -5.069938 -2.655073
H33 0.951263 -6.039684 -3.195051

C38 1.166178 -5.278149 -1.198857
H34 1.469956 -6.338185 -1.025974
H35 -1.452241 -5.551417 0.958689
H36 0.090209 -6.429069 0.972013
C39 1.547514 -4.547057 2.234973
H37 1.869053 -5.598083 2.424930
C40 1.552406 -3.771711 3.524957
H38 1.870388 -4.439374 4.360250
H39 -1.370329 -2.495174 4.857278
H40 0.155258 -2.745032 5.720204
C41 -0.781057 -0.102083 4.010960
O -1.913184 -0.323761 3.584901
N 2.526217 -0.678314 4.606411
H41 1.206279 -0.670952 6.255619
C42 1.002276 1.052120 4.986298
H42 0.832840 1.536570 5.976345
148
CBpath coordinate 4.41
C1 0.051540 -0.862648 0.115988
C2 1.037911 -1.179739 -0.831131
C3 2.170508 -0.470242 -0.460801
C4 1.818878 0.245245 0.698541
O 0.654118 -0.282033 1.200005
H1 0.894106 -1.755266 -1.735148
H2 3.098702 -0.383580 -1.009235
C5 -1.338753 -1.335831 0.345044
H3 -1.527936 -1.467918 1.409149
H4 -1.566862 -2.260370 -0.183949
N -2.331033 -0.281248 -0.159613
H5 -3.223785 -0.350000 0.355722
C6 -1.756546 1.131552 -0.030411
H6 -1.716255 1.358233 1.035411
H7 -2.485827 1.795285 -0.506930
C7 -0.414030 1.191364 -0.681171
C8 0.670494 1.867552 -0.144579
H8 0.575664 2.374484 0.812565
H9 1.426521 2.279665 -0.801170
H10 -0.415938 0.990404 -1.750061
H11 -2.513968 -0.483896 -1.156476
H12 2.468533 0.714684 1.426754
C1 1.215273 -0.420187 5.169757
N 0.112053 -1.055047 4.480937
C2 -0.260854 -2.425181 4.738629
N 0.174148 -3.355993 3.717744
C3 -0.653251 -3.867637 2.733154
O -1.880573 -3.788634 2.697460
N 0.159934 -4.493853 1.798207
C4 -0.353581 -5.414870 0.812079
N -0.093658 -4.974091 -0.537228
C5 -1.113119 -4.707937 -1.444651
O -2.306505 -4.537257 -1.190628
N -0.530938 -4.639190 -2.698227
C6 -1.144759 -3.923687 -3.797249
N -0.567736 -2.633351 -4.082956
C7 -1.157735 -1.434011 -3.697648
O -2.021469 -1.285314 -2.829925
N -0.668044 -0.442948 -4.540842
C8 -1.456306 0.716548 -4.889095
N -0.876237 1.959213 -4.443304
C9 -1.530403 2.849094 -3.607769
O -2.677734 2.731421 -3.178150

N -0.667095 3.913433 -3.391495
C10 -1.154305 5.226664 -3.035903
N -0.644357 5.686195 -1.768655
C11 -1.439583 6.326530 -0.825188
O -2.661170 6.484959 -0.871618
N -0.601083 6.725690 0.204412
C12 -0.987306 6.543004 1.598419
N -0.403207 5.422162 2.305089
C13 -0.990332 4.168781 2.380405
O -1.991080 3.804492 1.769763
N -0.294604 3.429021 3.335613
C14 -0.953488 2.366697 4.071857
N -0.183481 1.144347 4.147370
H1 -1.943115 2.147112 3.600309
H2 -1.192251 2.716896 5.102742
C15 0.700083 4.244679 4.013631
N 2.091576 3.865262 3.846836
C16 2.706665 2.850781 4.661716
N 2.232209 1.521098 4.375430
C17 3.130312 0.483789 4.162055
O 4.265493 0.580178 3.697413
H3 2.542534 3.090907 5.735645
H4 3.813648 2.878313 4.514443
C18 2.846418 4.908286 3.329938
O 4.072870 4.947863 3.241279
N 1.967644 5.886506 2.899846
C19 2.287520 6.636370 1.707301
N 1.764155 6.072380 0.495222
C20 2.327108 5.016440 -0.200682
O 3.265299 4.314094 0.169164
N 1.711350 4.974277 -1.444675
C21 2.442782 4.517276 -2.602659
N 1.751338 3.477002 -3.316719
C22 2.391011 2.315989 -3.725415
O 3.518225 1.957324 -3.390385
N 1.564068 1.695300 -4.650668
C23 2.086112 0.776702 -5.637159
N 1.633845 -0.578631 -5.435745
C24 2.479425 -1.672419 -5.334945
O 3.706120 -1.667597 -5.409636
N 1.677910 -2.782250 -5.117438
C25 2.169015 -4.009237 -4.530702
N 1.859314 -4.141259 -3.130335
C26 2.778671 -3.847541 -2.134201
O 3.851979 -3.266639 -2.287742
N 2.276891 -4.372818 -0.947590
C27 3.094874 -4.490857 0.239506
N 2.506826 -3.843353 1.393012
C28 3.180620 -2.861569 2.112485
O 4.213916 -2.282067 1.777814
N 2.517860 -2.710410 3.321794
C29 3.079741 -1.995763 4.439506
H5 4.188605 -1.930497 4.332407
H6 2.910270 -2.573571 5.374391
H7 4.105618 -4.054927 0.041895
H8 3.281365 -5.566169 0.460650
H9 3.273757 -4.091634 -4.683482
H10 1.733150 -4.869813 -5.088298
H11 3.204999 0.803620 -5.629235
H12 1.794188 1.112885 -6.658071
H13 3.456567 4.158025 -2.296216

H14 2.632457 5.368245 -3.291228
H15 3.391371 6.776254 1.607100
H16 1.873483 7.661640 1.830936
H17 0.455829 4.354418 5.095516
C30 0.611243 5.577913 3.330026
H18 0.277044 6.343267 4.069887
H19 -2.100266 6.474271 1.676645
H20 -0.721702 7.475354 2.146324
C31 0.770188 6.763190 -0.286791
H21 1.083102 7.823245 -0.449649
C32 0.750662 6.050971 -1.602988
H22 1.078296 6.743197 -2.408532
H23 -2.270867 5.208254 -2.999091
H24 -0.892132 5.959217 -3.833067
C33 0.577059 3.754437 -4.123134
H25 0.761873 4.619081 -4.804048
C34 0.385969 2.497586 -4.921922
H26 0.313332 2.748531 -6.005979
H27 -2.490356 0.610939 -4.475729
H28 -1.594239 0.762464 -5.992968
C35 0.245163 -0.985617 -5.528306
H29 -0.130844 -0.810457 -6.559600
C36 0.270587 -2.453712 -5.253336
H30 -0.160919 -2.998927 -6.126638
H31 -2.242302 -3.809436 -3.615104
H32 -1.070570 -4.562624 -4.707082
C37 0.858047 -5.073577 -2.656444
H33 0.953177 -6.044893 -3.193029
C38 1.166107 -5.276170 -1.199458
H34 1.471589 -6.335063 -1.022879
H35 -1.454171 -5.547507 0.956973
H36 0.088079 -6.425346 0.971924
C39 1.546154 -4.539844 2.229556
H37 1.870888 -5.590497 2.415957
C40 1.551965 -3.768290 3.521260
H38 1.874946 -4.437253 4.353577
H39 -1.369680 -2.494213 4.859811
H40 0.156842 -2.744126 5.721083
C41 -0.777673 -0.104764 4.002919
O -1.907186 -0.328892 3.571117
N 2.528176 -0.676304 4.613654
H41 1.201715 -0.667077 6.256999
C42 1.000382 1.053179 4.984425
H42 0.826890 1.538662 5.973263
148
CBpath coordinate 4.20
C1 0.042153 -0.826175 0.102569
C2 1.054229 -1.179092 -0.832337
C3 2.173436 -0.469231 -0.463671
C4 1.790924 0.302012 0.678984
O 0.652442 -0.284665 1.207213
H1 0.903177 -1.759993 -1.730575
H2 3.108521 -0.387702 -1.000710
C5 -1.341696 -1.328018 0.332910
H3 -1.525565 -1.470085 1.396655
H4 -1.566459 -2.249677 -0.201431
N -2.339082 -0.273386 -0.156688
H5 -3.222204 -0.336152 0.375738
C6 -1.752213 1.136002 -0.034450
H6 -1.715781 1.362660 1.032274
H7 -2.475508 1.804705 -0.512673

C7 -0.400864 1.150859 -0.672470
C8 0.715929 1.822553 -0.124690
H8 0.586529 2.372810 0.804813
H9 1.430353 2.276174 -0.802794
H10 -0.406551 0.977784 -1.746953
H11 -2.536900 -0.475709 -1.150374
H12 2.454792 0.742831 1.414386
C1 1.214724 -0.419459 5.169940
N 0.112444 -1.055191 4.480299
C2 -0.260531 -2.425049 4.738773
N 0.173889 -3.357038 3.718741
C3 -0.653866 -3.866971 2.733360
O -1.881049 -3.788198 2.698211
N 0.159285 -4.491992 1.797338
C4 -0.354118 -5.413374 0.811590
N -0.093555 -4.973242 -0.537787
C5 -1.113243 -4.708807 -1.445576
O -2.306530 -4.538045 -1.191899
N -0.531085 -4.642134 -2.699359
C6 -1.144719 -3.926339 -3.798211
N -0.567695 -2.635945 -4.083140
C7 -1.158975 -1.436854 -3.696029
O -2.022266 -1.289162 -2.830061
N -0.668300 -0.444969 -4.539009
C8 -1.457300 0.714505 -4.885454
N -0.876439 1.956620 -4.439135
C9 -1.529947 2.845543 -3.601717
O -2.675713 2.726660 -3.169630
N -0.667096 3.910945 -3.387451
C10 -1.155298 5.224237 -3.033062
N -0.644768 5.685299 -1.766577
C11 -1.439381 6.327187 -0.823824
O -2.661117 6.485674 -0.870098
N -0.600710 6.727121 0.205179
C12 -0.986731 6.545236 1.599333
N -0.403457 5.423873 2.305858
C13 -0.990302 4.170249 2.379272
O -1.990709 3.806664 1.767775
N -0.294511 3.429283 3.333528
C14 -0.953734 2.365780 4.067809
N -0.183126 1.143965 4.144566
H1 -1.942009 2.145373 3.593914
H2 -1.195199 2.715174 5.098312
C15 0.699450 4.244456 4.013286
N 2.091095 3.865591 3.847265
C16 2.706061 2.851518 4.662785
N 2.232069 1.521696 4.376462
C17 3.131525 0.484506 4.164695
O 4.266721 0.581220 3.702216
H3 2.541056 3.091782 5.736573
H4 3.813080 2.879377 4.516341
C18 2.846194 4.909089 3.331316
O 4.072470 4.948921 3.243323
N 1.967299 5.887355 2.901192
C19 2.287396 6.636960 1.708457
N 1.764358 6.072763 0.496240
C20 2.326671 5.016103 -0.198908
O 3.264805 4.313704 0.171512
N 1.710775 4.972854 -1.442743
C21 2.442540 4.515673 -2.600633
N 1.751211 3.475502 -3.315108

C22 2.391182 2.315197 -3.725145
O 3.519178 1.957269 -3.391551
N 1.563554 1.693878 -4.649232
C23 2.084866 0.775236 -5.636141
N 1.632620 -0.580120 -5.434869
C24 2.478075 -1.674191 -5.335642
O 3.704706 -1.669368 -5.410889
N 1.676533 -2.784262 -5.119205
C25 2.167481 -4.011724 -4.533190
N 1.859276 -4.144309 -3.132491
C26 2.779138 -3.849377 -2.137084
O 3.852524 -3.269095 -2.291712
N 2.277050 -4.372183 -0.949408
C27 3.094852 -4.486526 0.238240
N 2.504934 -3.839111 1.390906
C28 3.177785 -2.855655 2.110126
O 4.208908 -2.274227 1.774971
N 2.516263 -2.708028 3.321177
C29 3.079319 -1.995184 4.439404
H5 4.188088 -1.930736 4.331866
H6 2.909859 -2.574539 5.373396
H7 4.104520 -4.048364 0.040442
H8 3.283914 -5.561128 0.460760
H9 3.272021 -4.094514 -4.687134
H10 1.730672 -4.871932 -5.090624
H11 3.203766 0.801820 -5.628759
H12 1.792504 1.111674 -6.656829
H13 3.456029 4.155876 -2.293722
H14 2.633049 5.366608 -3.289105
H15 3.391272 6.776883 1.608527
H16 1.873317 7.662253 1.831799
H17 0.454170 4.352989 5.095073
C30 0.610910 5.578459 3.331025
H18 0.276834 6.343149 4.071640
H19 -2.099726 6.477434 1.677926
H20 -0.720236 7.477507 2.146984
C31 0.770587 6.763455 -0.286256
H21 1.084089 7.823167 -0.450194
C32 0.750455 6.049968 -1.601763
H22 1.077892 6.741485 -2.408025
H23 -2.271846 5.205102 -2.995493
H24 -0.894218 5.956267 -3.831138
C33 0.576134 3.752551 -4.120604
H25 0.760043 4.617279 -4.801696
C34 0.384747 2.495646 -4.919289
H26 0.310922 2.746621 -6.003266
H27 -2.490922 0.608566 -4.471332
H28 -1.596159 0.761102 -5.989233
C35 0.243827 -0.986751 -5.527176
H29 -0.132697 -0.810446 -6.558123
C36 0.269191 -2.455195 -5.253651
H30 -0.163294 -2.999446 -6.127115
H31 -2.242344 -3.812574 -3.616762
H32 -1.069574 -4.564808 -4.708357
C37 0.858065 -5.075891 -2.656898
H33 0.953331 -6.048079 -3.191874
C38 1.166319 -5.276083 -1.199549
H34 1.472115 -6.334617 -1.021295
H35 -1.454763 -5.545647 0.956269
H36 0.087317 -6.423871 0.972023
C39 1.545861 -4.537150 2.227471

H37 1.871699 -5.587777 2.412414
C40 1.552015 -3.767132 3.519942
H38 1.876733 -4.436843 4.351049
H39 -1.369282 -2.493669 4.860481
H40 0.157530 -2.743357 5.721266
C41 -0.776331 -0.105582 3.999174
O -1.905107 -0.330359 3.565117
N 2.528715 -0.675660 4.616907
H41 1.199345 -0.665591 6.257365
C42 0.999608 1.053661 4.983431
H42 0.824382 1.539589 5.971811
148
CBpath coordinate 3.98
C1 0.030556 -0.786908 0.084167
C2 1.072905 -1.176564 -0.836723
C3 2.172728 -0.464608 -0.473788
C4 1.765419 0.357687 0.661162
O 0.653133 -0.292811 1.213904
H1 0.931628 -1.783633 -1.721860
H2 3.117591 -0.392796 -0.998414
C5 -1.345047 -1.319995 0.322062
H3 -1.518235 -1.476332 1.386114
H4 -1.565151 -2.238069 -0.220765
N -2.350233 -0.265078 -0.149841
H5 -3.227376 -0.325435 0.392998
C6 -1.750236 1.140667 -0.033679
H6 -1.715569 1.372910 1.031468
H7 -2.458566 1.817176 -0.522713
C7 -0.391486 1.103617 -0.659136
C8 0.756857 1.785853 -0.097347
H8 0.576383 2.391474 0.789760
H9 1.424534 2.276652 -0.798835
H10 -0.402314 0.973586 -1.739549
H11 -2.558470 -0.465106 -1.141581
H12 2.453990 0.744914 1.404603
C1 1.214133 -0.418715 5.169679
N 0.112570 -1.055283 4.479824
C2 -0.260500 -2.424904 4.738901
N 0.173346 -3.357867 3.719517
C3 -0.654464 -3.866199 2.733373
O -1.881768 -3.787806 2.698541
N 0.158510 -4.490034 1.796379
C4 -0.354569 -5.411908 0.811098
N -0.093333 -4.972254 -0.538294
C5 -1.113052 -4.709414 -1.446409
O -2.306481 -4.538604 -1.192918
N -0.531132 -4.644543 -2.700288
C6 -1.144624 -3.928747 -3.799158
N -0.568141 -2.637930 -4.082838
C7 -1.159248 -1.439408 -3.695515
O -2.024164 -1.292211 -2.829898
N -0.669110 -0.446795 -4.537135
C8 -1.458024 0.712935 -4.882581
N -0.876559 1.954249 -4.434938
C9 -1.528876 2.842342 -3.596072
O -2.673579 2.722456 -3.160897
N -0.666950 3.908541 -3.383509
C10 -1.155920 5.221960 -3.030562
N -0.645089 5.684273 -1.764722
C11 -1.439389 6.327609 -0.822660
O -2.661053 6.485863 -0.868845

N -0.600357 6.728218 0.205796
C12 -0.986084 6.547071 1.600116
N -0.403487 5.425388 2.306510
C13 -0.989485 4.171298 2.377368
O -1.988078 3.808061 1.762856
N -0.294657 3.429642 3.331783
C14 -0.954221 2.365126 4.064447
N -0.183118 1.143718 4.142107
H1 -1.941501 2.144248 3.588794
H2 -1.197589 2.713851 5.094719
C15 0.698830 4.244227 4.013025
N 2.090750 3.865968 3.847612
C16 2.705496 2.852110 4.663520
N 2.232291 1.522187 4.376842
C17 3.131929 0.485117 4.167060
O 4.268598 0.581973 3.706716
H3 2.539698 3.092237 5.737189
H4 3.812611 2.880358 4.517746
C18 2.845762 4.909771 3.332427
O 4.072204 4.950033 3.244848
N 1.967027 5.888095 2.902347
C19 2.287239 6.637278 1.709352
N 1.764347 6.072799 0.497134
C20 2.325992 5.015276 -0.197215
O 3.263881 4.312738 0.173453
N 1.709703 4.971003 -1.440797
C21 2.442018 4.513937 -2.598474
N 1.750860 3.473866 -3.313250
C22 2.391436 2.314298 -3.724540
O 3.519955 1.957265 -3.392054
N 1.563253 1.692735 -4.648006
C23 2.084080 0.774054 -5.635231
N 1.631772 -0.581287 -5.433840
C24 2.477169 -1.675617 -5.336046
O 3.703739 -1.670852 -5.412125
N 1.675570 -2.785841 -5.120261
C25 2.166347 -4.013992 -4.535375
N 1.859754 -4.147411 -3.134422
C26 2.780557 -3.852492 -2.139995
O 3.854293 -3.273012 -2.295773
N 2.277822 -4.372241 -0.951279
C27 3.094925 -4.482862 0.237189
N 2.503299 -3.834933 1.388689
C28 3.174230 -2.850293 2.107835
O 4.204312 -2.266413 1.771683
N 2.515092 -2.705701 3.320239
C29 3.078851 -1.994535 4.439109
H5 4.187634 -1.930701 4.331096
H6 2.909502 -2.575007 5.372363
H7 4.103762 -4.042756 0.039311
H8 3.286200 -5.556719 0.461267
H9 3.270678 -4.097236 -4.690454
H10 1.728495 -4.873721 -5.092683
H11 3.202985 0.800499 -5.628258
H12 1.791386 1.110595 -6.655800
H13 3.455396 4.154037 -2.291245
H14 2.632926 5.364923 -3.286849
H15 3.391133 6.777194 1.609475
H16 1.873194 7.662614 1.832393
H17 0.452766 4.351605 5.094736
C30 0.610605 5.578851 3.331980

H18 0.276543 6.342939 4.073198
H19 -2.099082 6.480092 1.679161
H20 -0.718571 7.479178 2.147560
C31 0.770913 6.763518 -0.285791
H21 1.084983 7.822936 -0.450586
C32 0.750217 6.048801 -1.600602
H22 1.077786 6.739434 -2.407615
H23 -2.272491 5.202312 -2.992636
H24 -0.895464 5.953410 -3.829418
C33 0.575494 3.750712 -4.118248
H25 0.758702 4.615662 -4.799297
C34 0.383795 2.493882 -4.916935
H26 0.308524 2.744772 -6.000840
H27 -2.491549 0.606891 -4.468085
H28 -1.597354 0.760562 -5.986246
C35 0.242999 -0.987771 -5.526156
H29 -0.133778 -0.810606 -6.556859
C36 0.268333 -2.456366 -5.253804
H30 -0.164696 -2.999823 -6.127464
H31 -2.242465 -3.815613 -3.618276
H32 -1.068713 -4.566811 -4.709486
C37 0.858206 -5.078001 -2.657361
H33 0.953448 -6.050913 -3.190929
C38 1.166608 -5.276165 -1.199696
H34 1.472297 -6.334449 -1.019958
H35 -1.455273 -5.544045 0.955565
H36 0.086760 -6.422338 0.972235
C39 1.545340 -4.534421 2.225495
H37 1.872127 -5.584954 2.409270
C40 1.551797 -3.765859 3.518555
H38 1.878074 -4.436182 4.348539
H39 -1.369186 -2.493223 4.861092
H40 0.157894 -2.742696 5.721405
C41 -0.775688 -0.106307 3.996083
O -1.903953 -0.331692 3.560969
N 2.529370 -0.674932 4.619060
H41 1.197579 -0.663967 6.257275
C42 0.998842 1.054097 4.982123
H42 0.822395 1.540277 5.970164
148
CBpath coordinate 3.77
C1 0.019000 -0.723323 0.052250
C2 1.089168 -1.163289 -0.851101
C3 2.184153 -0.457547 -0.482809
C4 1.759266 0.394486 0.650887
O 0.661730 -0.295980 1.215506
H1 0.958193 -1.792904 -1.719845
H2 3.142675 -0.413067 -0.982481
C5 -1.344851 -1.301674 0.297829
H3 -1.497058 -1.477495 1.361787
H4 -1.555267 -2.212388 -0.260094
N -2.370140 -0.250795 -0.141580
H5 -3.229946 -0.310236 0.429317
C6 -1.753175 1.145195 -0.028791
H6 -1.728575 1.381365 1.035890
H7 -2.427000 1.841336 -0.535399
C7 -0.380730 1.038626 -0.631462
C8 0.782283 1.763784 -0.078601
H8 0.568302 2.401361 0.779633
H9 1.403585 2.280007 -0.806203
H10 -0.400594 0.934984 -1.716977

H11 -2.610566 -0.443665 -1.126262
H12 2.462665 0.752527 1.398132
C1 1.212946 -0.417422 5.168976
N 0.112725 -1.055205 4.478260
C2 -0.260646 -2.424380 4.738651
N 0.172323 -3.358768 3.720323
C3 -0.655755 -3.863894 2.732507
O -1.883041 -3.785249 2.698032
N 0.157094 -4.486592 1.794565
C4 -0.355327 -5.409382 0.810121
N -0.092702 -4.970673 -0.539209
C5 -1.112458 -4.709630 -1.447686
O -2.305826 -4.538060 -1.194573
N -0.530787 -4.648144 -2.701825
C6 -1.144101 -3.932291 -3.800550
N -0.568260 -2.641029 -4.082485
C7 -1.160694 -1.443284 -3.695027
O -2.027832 -1.296813 -2.831711
N -0.669872 -0.449671 -4.534730
C8 -1.458891 0.710444 -4.878015
N -0.876227 1.949811 -4.427208
C9 -1.526810 2.836103 -3.585035
O -2.669348 2.714029 -3.144893
N -0.666551 3.904379 -3.376741
C10 -1.156933 5.218037 -3.026445
N -0.645692 5.682681 -1.761704
C11 -1.439482 6.328037 -0.820666
O -2.661104 6.486035 -0.866569
N -0.599865 6.730045 0.206861
C12 -0.985086 6.550228 1.601399
N -0.403627 5.427992 2.307734
C13 -0.987824 4.172974 2.374055
O -1.983777 3.809873 1.755312
N -0.294549 3.430280 3.328910
C14 -0.954883 2.364320 4.058808
N -0.183031 1.143461 4.137516
H1 -1.940678 2.142954 3.580438
H2 -1.201169 2.711886 5.088785
C15 0.697912 4.244131 4.012616
N 2.090196 3.866734 3.848344
C16 2.704590 2.853100 4.664633
N 2.232298 1.523089 4.377299
C17 3.132980 0.486238 4.170937
O 4.271113 0.583400 3.714016
H3 2.537651 3.092861 5.738189
H4 3.811817 2.881919 4.519864
C18 2.845232 4.911157 3.334375
O 4.071777 4.951817 3.247521
N 1.966661 5.889565 2.904449
C19 2.287072 6.637878 1.710844
N 1.764339 6.072827 0.498791
C20 2.325069 5.014019 -0.194261
O 3.262679 4.311440 0.177149
N 1.708585 4.968431 -1.437657
C21 2.441608 4.511135 -2.594900
N 1.750915 3.471249 -3.310346
C22 2.392216 2.312850 -3.723551
O 3.521866 1.957216 -3.393335
N 1.563089 1.690674 -4.645720
C23 2.083010 0.772080 -5.633551
N 1.630726 -0.583280 -5.432140

C24 2.475946 -1.677941 -5.336365
O 3.702490 -1.673306 -5.413724
N 1.674325 -2.788331 -5.121462
C25 2.164741 -4.017997 -4.539263
N 1.861091 -4.153200 -3.137792
C26 2.783675 -3.858810 -2.144877
O 3.859056 -3.282795 -2.302658
N 2.279298 -4.372628 -0.954312
C27 3.095153 -4.477158 0.235619
N 2.500349 -3.828064 1.384896
C28 3.168745 -2.841354 2.103643
O 4.196322 -2.253616 1.766067
N 2.512706 -2.701650 3.318476
C29 3.077965 -1.993399 4.438220
H5 4.186715 -1.930573 4.329367
H6 2.908875 -2.575896 5.370244
H7 4.102723 -4.034136 0.037845
H8 3.289878 -5.549747 0.462771
H9 3.268681 -4.101956 -4.696677
H10 1.724981 -4.876588 -5.096811
H11 3.201921 0.798244 -5.627229
H12 1.789738 1.108912 -6.653858
H13 3.454619 4.150792 -2.286949
H14 2.633289 5.362072 -3.283144
H15 3.390999 6.777645 1.611006
H16 1.873163 7.663330 1.833181
H17 0.450479 4.349642 5.094201
C30 0.610167 5.579755 3.333586
H18 0.276145 6.342823 4.075842
H19 -2.098077 6.484594 1.681222
H20 -0.715928 7.482173 2.148334
C31 0.771384 6.763413 -0.284957
H21 1.086489 7.822280 -0.451287
C32 0.749840 6.046789 -1.598639
H22 1.077473 6.736104 -2.406778
H23 -2.273506 5.197598 -2.988240
H24 -0.897222 5.948378 -3.826624
C33 0.574521 3.747481 -4.113939
H25 0.756417 4.612672 -4.795049
C34 0.382337 2.490730 -4.912420
H26 0.304469 2.741539 -5.996192
H27 -2.492381 0.604078 -4.463510
H28 -1.598294 0.760271 -5.981590
C35 0.241910 -0.989453 -5.524599
H29 -0.135204 -0.811034 -6.554961
C36 0.267208 -2.458334 -5.253820
H30 -0.166663 -3.000733 -6.127699
H31 -2.242205 -3.820159 -3.620566
H32 -1.066841 -4.569678 -4.711218
C37 0.858595 -5.081686 -2.658165
H33 0.953206 -6.055996 -3.189275
C38 1.167241 -5.276511 -1.199949
H34 1.472497 -6.334505 -1.017783
H35 -1.456123 -5.541226 0.954094
H36 0.085889 -6.419664 0.972501
C39 1.544334 -4.529765 2.221937
H37 1.872641 -5.580161 2.403795
C40 1.551236 -3.763544 3.515929
H38 1.880099 -4.434896 4.344101
H39 -1.369225 -2.492243 4.861749
H40 0.158402 -2.741267 5.721173

C41 -0.774299 -0.107277 3.989844
O -1.901261 -0.333714 3.551713
N 2.530037 -0.673720 4.622541
H41 1.194101 -0.661355 6.256824
C42 0.997449 1.054942 4.979729
H42 0.818843 1.541567 5.967191
148
CBpath coordinate 3.56
C1 0.007133 -0.664627 0.023366
C2 1.099681 -1.143558 -0.863983
C3 2.189322 -0.448654 -0.489095
C4 1.750021 0.432526 0.639725
O 0.663085 -0.288894 1.212847
H1 0.969416 -1.787412 -1.724394
H2 3.163885 -0.431033 -0.961463
C5 -1.342297 -1.288065 0.273957
H3 -1.472330 -1.496562 1.334691
H4 -1.541849 -2.185687 -0.309093
N -2.384424 -0.236885 -0.123564
H5 -3.223376 -0.292887 0.479643
C6 -1.747942 1.148774 -0.021727
H6 -1.724670 1.394146 1.041307
H7 -2.393566 1.860532 -0.542143
C7 -0.369566 0.981758 -0.612262
C8 0.809126 1.739948 -0.065600
H8 0.568307 2.403337 0.766614
H9 1.396297 2.268897 -0.812439
H10 -0.400847 0.910101 -1.699498
H11 -2.662869 -0.422129 -1.099754
H12 2.460319 0.773665 1.388280
C1 1.211503 -0.415309 5.168223
N 0.113751 -1.054736 4.475127
C2 -0.260853 -2.422904 4.737544
N 0.170709 -3.359442 3.720644
C3 -0.657496 -3.860956 2.731387
O -1.884874 -3.781716 2.697044
N 0.155042 -4.482040 1.791923
C4 -0.356763 -5.405818 0.808631
N -0.092425 -4.968354 -0.540642
C5 -1.111961 -4.709340 -1.449751
O -2.305207 -4.536166 -1.196985
N -0.530660 -4.652705 -2.704166
C6 -1.143755 -3.937883 -3.803488
N -0.569619 -2.645760 -4.084054
C7 -1.164901 -1.449522 -3.697109
O -2.035497 -1.304747 -2.837147
N -0.672025 -0.454074 -4.533210
C8 -1.459980 0.707461 -4.872903
N -0.874948 1.943292 -4.415932
C9 -1.523744 2.827521 -3.570576
O -2.663943 2.703051 -3.124960
N -0.665577 3.898489 -3.367200
C10 -1.158143 5.212107 -3.020336
N -0.646208 5.680591 -1.757546
C11 -1.439270 6.328879 -0.817833
O -2.660950 6.486801 -0.863683
N -0.599105 6.732724 0.208242
C12 -0.983711 6.554619 1.603199
N -0.403759 5.431806 2.309155
C13 -0.986599 4.175847 2.370838
O -1.980859 3.814010 1.748666

N -0.294361 3.431418 3.324683
C14 -0.955366 2.363232 4.050962
N -0.182065 1.143516 4.130620
H1 -1.939199 2.140954 3.569043
H2 -1.205534 2.709086 5.080556
C15 0.696717 4.244172 4.011975
N 2.089466 3.868050 3.849175
C16 2.703500 2.854808 4.666430
N 2.232562 1.524593 4.378397
C17 3.134548 0.488025 4.176324
O 4.274111 0.585404 3.723039
H3 2.534913 3.094382 5.739743
H4 3.810846 2.884409 4.523052
C18 2.844646 4.913051 3.337147
O 4.071185 4.954414 3.251253
N 1.966135 5.891838 2.907151
C19 2.286984 6.639019 1.713015
N 1.764648 6.073279 0.500912
C20 2.324210 5.012637 -0.190202
O 3.261065 4.309710 0.182287
N 1.707726 4.965512 -1.433648
C21 2.441886 4.507895 -2.590168
N 1.751957 3.468333 -3.306774
C22 2.394379 2.311323 -3.722481
O 3.525548 1.957852 -3.395516
N 1.563553 1.687899 -4.642166
C23 2.081893 0.769303 -5.631005
N 1.629378 -0.585936 -5.429346
C24 2.474544 -1.681077 -5.336316
O 3.700994 -1.676495 -5.414753
N 1.672771 -2.791692 -5.123358
C25 2.162405 -4.023079 -4.543983
N 1.861802 -4.159007 -3.141916
C26 2.786079 -3.864170 -2.150732
O 3.863623 -3.292886 -2.311072
N 2.279889 -4.370839 -0.957913
C27 3.094876 -4.469067 0.233213
N 2.496460 -3.819216 1.379922
C28 3.162097 -2.830057 2.098748
O 4.186941 -2.238400 1.759586
N 2.509173 -2.695951 3.315668
C29 3.076673 -1.991668 4.436980
H5 4.185336 -1.930383 4.326909
H6 2.907932 -2.577262 5.367090
H7 4.100999 -4.022781 0.035414
H8 3.293516 -5.540344 0.463407
H9 3.265812 -4.108689 -4.704139
H10 1.719781 -4.880298 -5.101400
H11 3.200822 0.795023 -5.625904
H12 1.787634 1.106629 -6.650870
H13 3.454368 4.146945 -2.281063
H14 2.634989 5.358826 -3.278107
H15 3.390938 6.778696 1.613317
H16 1.873160 7.664617 1.834395
H17 0.447269 4.347240 5.093303
C30 0.609622 5.581172 3.335625
H18 0.275543 6.342945 4.079210
H19 -2.096738 6.490794 1.683877
H20 -0.712472 7.486368 2.149467
C31 0.772207 6.763535 -0.283764
H21 1.088456 7.821715 -0.452177

C32 0.749584 6.044338 -1.595972
H22 1.076999 6.732009 -2.405666
H23 -2.274732 5.190149 -2.981169
H24 -0.900121 5.940806 -3.822645
C33 0.573591 3.742964 -4.107848
H25 0.752930 4.608409 -4.789395
C34 0.380867 2.486058 -4.905834
H26 0.299032 2.736465 -5.989395
H27 -2.493778 0.601143 -4.459078
H28 -1.598577 0.761797 -5.976386
C35 0.240558 -0.991889 -5.523446
H29 -0.136045 -0.811745 -6.553691
C36 0.265797 -2.461281 -5.255039
H30 -0.168223 -3.002308 -6.129606
H31 -2.242251 -3.827549 -3.624790
H32 -1.064259 -4.574849 -4.714198
C37 0.858959 -5.085619 -2.659173
H33 0.953511 -6.061709 -3.187007
C38 1.167774 -5.275745 -1.200278
H34 1.473399 -6.333073 -1.014812
H35 -1.457720 -5.537084 0.951890
H36 0.084118 -6.415987 0.972629
C39 1.542794 -4.523606 2.217229
H37 1.872912 -5.573891 2.396625
C40 1.550269 -3.760163 3.512338
H38 1.882182 -4.432682 4.338316
H39 -1.369385 -2.489522 4.861612
H40 0.158830 -2.738800 5.720065
C41 -0.770754 -0.108193 3.979648
O -1.895174 -0.336400 3.535419
N 2.531133 -0.671929 4.627799
H41 1.188773 -0.657836 6.256306
C42 0.995816 1.056407 4.976739
H42 0.813661 1.543778 5.963229
148
CBpath coordinate 3.37
C1 -0.007585 -0.619256 0.000937
C2 1.101682 -1.129749 -0.871469
C3 2.185840 -0.434881 -0.496445
C4 1.733120 0.471548 0.624811
O 0.656793 -0.275379 1.208981
H1 0.978206 -1.792850 -1.718701
H2 3.166960 -0.428588 -0.956510
C5 -1.343741 -1.279898 0.263440
H3 -1.461402 -1.502297 1.323151
H4 -1.537184 -2.174152 -0.326408
N -2.392390 -0.233106 -0.114470
H5 -3.225199 -0.286944 0.497775
C6 -1.742455 1.145130 -0.014649
H6 -1.715712 1.396233 1.046471
H7 -2.374186 1.862155 -0.543312
C7 -0.358599 0.932919 -0.596034
C8 0.836429 1.710282 -0.050563
H8 0.571939 2.393452 0.759047
H9 1.400250 2.248828 -0.811058
H10 -0.395404 0.899810 -1.687574
H11 -2.679290 -0.413809 -1.087556
H12 2.451824 0.791297 1.376461
C1 1.211012 -0.414400 5.167861
N 0.114413 -1.054434 4.473827
C2 -0.260912 -2.422224 4.737023

N 0.170311 -3.359876 3.721155
C3 -0.658326 -3.860322 2.731240
O -1.885412 -3.781181 2.697281
N 0.154338 -4.480502 1.791148
C4 -0.357483 -5.404301 0.807885
N -0.092647 -4.967080 -0.541349
C5 -1.112271 -4.709433 -1.450856
O -2.305487 -4.536278 -1.198474
N -0.530844 -4.654637 -2.705397
C6 -1.143725 -3.940628 -3.805354
N -0.570522 -2.648116 -4.085596
C7 -1.166632 -1.452330 -3.698243
O -2.038210 -1.308321 -2.839300
N -0.673001 -0.456116 -4.532857
C8 -1.460367 0.706254 -4.871098
N -0.874292 1.940548 -4.411747
C9 -1.522685 2.824348 -3.565441
O -2.662066 2.699044 -3.118302
N -0.665209 3.896005 -3.363499
C10 -1.158733 5.209667 -3.017750
N -0.646350 5.679735 -1.755702
C11 -1.439031 6.329410 -0.816802
O -2.660657 6.487514 -0.862585
N -0.598561 6.734037 0.208878
C12 -0.983061 6.556676 1.603850
N -0.403787 5.433418 2.309814
C13 -0.986630 4.177423 2.369651
O -1.980388 3.816291 1.746811
N -0.294233 3.431927 3.323020
C14 -0.955474 2.362878 4.047695
N -0.181416 1.143661 4.127887
H1 -1.938370 2.140007 3.564288
H2 -1.207289 2.707958 5.077154
C15 0.696222 4.244257 4.011608
N 2.089240 3.868514 3.849464
C16 2.703061 2.855709 4.667196
N 2.232852 1.525342 4.378945
C17 3.135262 0.488809 4.178599
O 4.275624 0.586387 3.726985
H3 2.533682 3.095289 5.740400
H4 3.810513 2.885613 4.524532
C18 2.844334 4.914031 3.338145
O 4.071042 4.955502 3.252674
N 1.966050 5.892704 2.908295
C19 2.286966 6.639579 1.713857
N 1.764949 6.073440 0.501833
C20 2.323498 5.011913 -0.188524
O 3.260095 4.308421 0.184634
N 1.707292 4.963992 -1.431769
C21 2.441973 4.506564 -2.588132
N 1.752360 3.466969 -3.305068
C22 2.395095 2.310732 -3.721793
O 3.526983 1.958014 -3.395905
N 1.563844 1.686675 -4.640594
C23 2.081481 0.768094 -5.629786
N 1.628749 -0.587107 -5.428123
C24 2.473755 -1.682395 -5.336342
O 3.700264 -1.677832 -5.414738
N 1.671918 -2.793294 -5.124938
C25 2.161215 -4.024892 -4.545798
N 1.861555 -4.160680 -3.143531

C26 2.786204 -3.864865 -2.152931
O 3.863837 -3.294230 -2.314126
N 2.279648 -4.369470 -0.959322
C27 3.094579 -4.465483 0.232012
N 2.494945 -3.815519 1.378156
C28 3.159644 -2.825782 2.096832
O 4.183755 -2.232923 1.757570
N 2.507846 -2.693595 3.314874
C29 3.076226 -1.990941 4.436537
H5 4.184891 -1.930230 4.326038
H6 2.907625 -2.577678 5.365948
H7 4.099993 -4.017708 0.034100
H8 3.294878 -5.536257 0.463150
H9 3.264458 -4.111112 -4.706700
H10 1.717681 -4.881886 -5.102828
H11 3.200424 0.793482 -5.625126
H12 1.786915 1.105603 -6.649497
H13 3.454388 4.145595 -2.278686
H14 2.635551 5.357545 -3.275900
H15 3.390952 6.779287 1.614338
H16 1.873124 7.665181 1.834930
H17 0.446066 4.346304 5.092906
C30 0.609393 5.581869 3.336415
H18 0.275405 6.343063 4.080608
H19 -2.096078 6.493644 1.684901
H20 -0.710925 7.488282 2.149938
C31 0.772692 6.763671 -0.283361
H21 1.089564 7.821530 -0.452646
C32 0.749560 6.043277 -1.594858
H22 1.076911 6.730218 -2.405205
H23 -2.275290 5.186974 -2.977871
H24 -0.901746 5.937786 -3.820915
C33 0.573272 3.741171 -4.105403
H25 0.751690 4.606672 -4.787106
C34 0.380464 2.484197 -4.903250
H26 0.297332 2.734442 -5.986771
H27 -2.494303 0.599882 -4.457654
H28 -1.598569 0.762394 -5.974548
C35 0.239930 -0.992838 -5.523295
H29 -0.136234 -0.811587 -6.553487
C36 0.265005 -2.462521 -5.256304
H30 -0.169051 -3.002702 -6.131373
H31 -2.242441 -3.831197 -3.627529
H32 -1.062924 -4.577673 -4.715919
C37 0.858925 -5.086889 -2.659620
H33 0.953890 -6.063645 -3.186136
C38 1.167724 -5.274974 -1.200439
H34 1.473576 -6.332013 -1.013588
H35 -1.458482 -5.535254 0.950964
H36 0.083139 -6.414540 0.972177
C39 1.542313 -4.521258 2.215444
H37 1.873308 -5.571422 2.393901
C40 1.549972 -3.758876 3.511039
H38 1.883246 -4.431783 4.336201
H39 -1.369417 -2.488115 4.861232
H40 0.158751 -2.737555 5.719760
C41 -0.769305 -0.108495 3.975348
O -1.892534 -0.337277 3.529004
N 2.531760 -0.671032 4.629975
H41 1.186744 -0.656322 6.256047
C42 0.995245 1.057104 4.975526

H42 0.811654 1.544769 5.961624
148
CBpath coordinate 3.19
C1 -0.017016 -0.580551 -0.016759
C2 1.104350 -1.113768 -0.879019
C3 2.185744 -0.417525 -0.502512
C4 1.718637 0.509716 0.609576
O 0.655579 -0.262611 1.207267
H1 0.984053 -1.793736 -1.712574
H2 3.174751 -0.425617 -0.945267
C5 -1.342296 -1.270593 0.248202
H3 -1.442814 -1.506090 1.307034
H4 -1.528411 -2.157933 -0.353012
N -2.402803 -0.225724 -0.104976
H5 -3.223558 -0.279249 0.523690
C6 -1.735229 1.143010 -0.007091
H6 -1.712863 1.395609 1.053672
H7 -2.337992 1.875119 -0.547075
C7 -0.349515 0.884383 -0.577635
C8 0.857518 1.678941 -0.038982
H8 0.572807 2.380567 0.749939
H9 1.402820 2.219911 -0.812950
H10 -0.393695 0.867904 -1.671239
H11 -2.711839 -0.399117 -1.073066
H12 2.442573 0.828188 1.357596
C1 1.210509 -0.413285 5.167240
N 0.114925 -1.054082 4.472003
C2 -0.260979 -2.421340 4.736387
N 0.169512 -3.360260 3.721463
C3 -0.658907 -3.858996 2.730806
O -1.886382 -3.779606 2.697043
N 0.153235 -4.478308 1.789973
C4 -0.358437 -5.402315 0.806970
N -0.093169 -4.965414 -0.542329
C5 -1.112514 -4.709308 -1.452186
O -2.305927 -4.535779 -1.200310
N -0.531187 -4.656825 -2.706970
C6 -1.143685 -3.943883 -3.807615
N -0.571535 -2.650708 -4.087461
C7 -1.168826 -1.455883 -3.700285
O -2.042419 -1.312644 -2.843340
N -0.674017 -0.458356 -4.532643
C8 -1.460717 0.704706 -4.868973
N -0.873438 1.937153 -4.406220
C9 -1.520930 2.819775 -3.558398
O -2.659333 2.693318 -3.108730
N -0.664778 3.892947 -3.358814
C10 -1.159415 5.206575 -3.014655
N -0.646671 5.678674 -1.753653
C11 -1.438735 6.330131 -0.815410
O -2.660446 6.488400 -0.861217
N -0.598000 6.735524 0.209450
C12 -0.982202 6.558961 1.604690
N -0.403914 5.435320 2.310401
C13 -0.985592 4.178604 2.367783
O -1.978414 3.817731 1.742822
N -0.294027 3.432487 3.320690
C14 -0.955568 2.362442 4.043868
N -0.180949 1.143811 4.124509
H1 -1.937551 2.139153 3.558753
H2 -1.209280 2.706766 5.073091

C15 0.695747 4.244293 4.011183
N 2.088926 3.869272 3.849705
C16 2.702610 2.856668 4.668075
N 2.233087 1.526229 4.379325
C17 3.136379 0.489888 4.181161
O 4.277540 0.587755 3.732026
H3 2.532193 3.096198 5.741106
H4 3.810104 2.887087 4.526282
C18 2.844208 4.914941 3.339472
O 4.070760 4.956825 3.254348
N 1.965777 5.893939 2.909536
C19 2.287015 6.640028 1.714879
N 1.765132 6.073254 0.502819
C20 2.322524 5.009974 -0.185821
O 3.257980 4.305743 0.187881
N 1.706238 4.961552 -1.429425
C21 2.441802 4.504447 -2.585249
N 1.752596 3.465105 -3.302660
C22 2.396247 2.309556 -3.720773
O 3.528659 1.958195 -3.396173
N 1.564250 1.685209 -4.638587
C23 2.081223 0.766573 -5.628323
N 1.628114 -0.588413 -5.426815
C24 2.473041 -1.684170 -5.336518
O 3.699456 -1.679555 -5.414971
N 1.670998 -2.795139 -5.126882
C25 2.159870 -4.027099 -4.547907
N 1.861136 -4.162391 -3.145527
C26 2.786005 -3.865185 -2.155404
O 3.863998 -3.295584 -2.317638
N 2.278969 -4.367024 -0.961009
C27 3.094007 -4.460743 0.230483
N 2.492927 -3.810827 1.375729
C28 3.156539 -2.820144 2.094455
O 4.179523 -2.225828 1.754529
N 2.506248 -2.690764 3.313634
C29 3.075797 -1.989951 4.435948
H5 4.184411 -1.930026 4.324906
H6 2.907292 -2.578076 5.364510
H7 4.098703 -4.011426 0.032390
H8 3.296157 -5.530988 0.462536
H9 3.262920 -4.114058 -4.709654
H10 1.715289 -4.883839 -5.104501
H11 3.200139 0.791673 -5.623963
H12 1.786374 1.104500 -6.647817
H13 3.454113 4.143593 -2.275408
H14 2.635677 5.355571 -3.272890
H15 3.390959 6.779828 1.615364
H16 1.873084 7.665696 1.835308
H17 0.444561 4.345120 5.092319
C30 0.609182 5.582561 3.337353
H18 0.275152 6.343106 4.082212
H19 -2.095260 6.497037 1.686107
H20 -0.708940 7.490437 2.150456
C31 0.773366 6.763471 -0.282805
H21 1.091022 7.820894 -0.453351
C32 0.749495 6.041533 -1.593371
H22 1.077058 6.727467 -2.404593
H23 -2.275971 5.183007 -2.974110
H24 -0.903505 5.933948 -3.818904
C33 0.572970 3.738687 -4.102139

H25 0.750440 4.604406 -4.783896
C34 0.380015 2.481745 -4.899929
H26 0.295111 2.731930 -5.983316
H27 -2.494866 0.598406 -4.456001
H28 -1.598431 0.763291 -5.972362
C35 0.239386 -0.993995 -5.523219
H29 -0.136345 -0.811444 -6.553339
C36 0.264186 -2.464019 -5.257840
H30 -0.169996 -3.003105 -6.133456
H31 -2.242708 -3.835557 -3.630935
H32 -1.061218 -4.580961 -4.717982
C37 0.858861 -5.088220 -2.660100
H33 0.954384 -6.065824 -3.184965
C38 1.167585 -5.273671 -1.200604
H34 1.474046 -6.330197 -1.011975
H35 -1.459518 -5.532945 0.949833
H36 0.081918 -6.412586 0.971651
C39 1.541626 -4.518140 2.213140
H37 1.873713 -5.568172 2.390371
C40 1.549666 -3.757155 3.509316
H38 1.884470 -4.430679 4.333362
H39 -1.369541 -2.486523 4.860858
H40 0.158685 -2.735999 5.719295
C41 -0.767349 -0.108758 3.970572
O -1.889800 -0.338307 3.521430
N 2.532423 -0.670042 4.632343
H41 1.184290 -0.654559 6.255515
C42 0.994679 1.057890 4.973929
H42 0.809406 1.545805 5.959605
148
CBpath coordinate 3.03
C1 -0.022988 -0.543986 -0.034964
C2 1.115240 -1.097779 -0.877959
C3 2.190088 -0.397252 -0.499217
C4 1.701838 0.556154 0.594765
O 0.648997 -0.236860 1.204704
H1 0.998794 -1.790241 -1.701931
H2 3.185433 -0.407488 -0.926220
C5 -1.337483 -1.262585 0.226123
H3 -1.421444 -1.539105 1.275942
H4 -1.521989 -2.126537 -0.409575
N -2.408288 -0.209345 -0.077729
H5 -3.203418 -0.256109 0.587126
C6 -1.715114 1.147873 0.002134
H6 -1.677952 1.408855 1.059681
H7 -2.297117 1.891296 -0.543710
C7 -0.337821 0.839507 -0.574257
C8 0.881047 1.652141 -0.045341
H8 0.572263 2.378792 0.712696
H9 1.415094 2.184849 -0.833731
H10 -0.395600 0.833799 -1.667559
H11 -2.763756 -0.371077 -1.029884
H12 2.425330 0.881528 1.340723
C1 1.209268 -0.410492 5.166147
N 0.116801 -1.053064 4.467974
C2 -0.261086 -2.419073 4.734904
N 0.168041 -3.360754 3.722141
C3 -0.660929 -3.856257 2.730278
O -1.888399 -3.776396 2.697370
N 0.150582 -4.472500 1.786991
C4 -0.361162 -5.396714 0.804399

N -0.094515 -4.960705 -0.544781
C5 -1.113646 -4.708718 -1.456225
O -2.307166 -4.534695 -1.205294
N -0.532093 -4.662210 -2.711030
C6 -1.143619 -3.952112 -3.814140
N -0.574135 -2.657895 -4.093236
C7 -1.175010 -1.464844 -3.706250
O -2.053318 -1.325110 -2.854001
N -0.676877 -0.464603 -4.532672
C8 -1.461703 0.701090 -4.864135
N -0.871273 1.928354 -4.393061
C9 -1.517241 2.808981 -3.541700
O -2.653302 2.679678 -3.087085
N -0.663641 3.884894 -3.347262
C10 -1.161228 5.198446 -3.006826
N -0.647444 5.675734 -1.748281
C11 -1.438327 6.331663 -0.812356
O -2.659740 6.491217 -0.858084
N -0.596465 6.739067 0.211129
C12 -0.980143 6.564384 1.606515
N -0.403711 5.439632 2.311930
C13 -0.982951 4.182048 2.362709
O -1.973416 3.822007 1.733432
N -0.292823 3.433503 3.314976
C14 -0.955582 2.361681 4.034060
N -0.179269 1.144215 4.116536
H1 -1.935253 2.137277 3.544836
H2 -1.213879 2.704405 5.062693
C15 0.694670 4.244468 4.009930
N 2.088485 3.870854 3.850811
C16 2.701509 2.859203 4.670346
N 2.233865 1.528446 4.380512
C17 3.138867 0.492509 4.188057
O 4.282086 0.590977 3.744115
H3 2.528595 3.098400 5.743070
H4 3.809264 2.890694 4.530804
C18 2.843833 4.917868 3.342772
O 4.070470 4.960078 3.258842
N 1.965613 5.896763 2.913146
C19 2.287162 6.640934 1.717137
N 1.765614 6.072634 0.505813
C20 2.319764 5.005037 -0.179422
O 3.252481 4.298603 0.196834
N 1.704479 4.955893 -1.423297
C21 2.442056 4.499110 -2.578049
N 1.754169 3.460221 -3.297255
C22 2.399077 2.306628 -3.717919
O 3.532956 1.957280 -3.396358
N 1.565444 1.680885 -4.633544
C23 2.080808 0.762915 -5.624557
N 1.626791 -0.592006 -5.423754
C24 2.471262 -1.688251 -5.337419
O 3.697731 -1.683822 -5.415542
N 1.668884 -2.799982 -5.132288
C25 2.156641 -4.032231 -4.553263
N 1.859855 -4.165806 -3.150340
C26 2.784710 -3.864643 -2.161822
O 3.863095 -3.296185 -2.326254
N 2.277176 -4.360579 -0.965219
C27 3.092454 -4.448518 0.226319
N 2.488058 -3.799015 1.370014

C28 3.149246 -2.807104 2.089062
O 4.169745 -2.208784 1.748222
N 2.502269 -2.683544 3.310712
C29 3.074613 -1.987526 4.434381
H5 4.183182 -1.929290 4.321932
H6 2.906574 -2.578941 5.360913
H7 4.095189 -3.995050 0.027732
H8 3.299246 -5.517446 0.460426
H9 3.259300 -4.121209 -4.716536
H10 1.709654 -4.888672 -5.108380
H11 3.199761 0.787012 -5.620932
H12 1.785557 1.102036 -6.643550
H13 3.453734 4.137887 -2.266571
H14 2.637252 5.350500 -3.264984
H15 3.391101 6.780697 1.617642
H16 1.873196 7.666772 1.835933
H17 0.440748 4.342137 5.090772
C30 0.608776 5.584401 3.339621
H18 0.274416 6.343132 4.086119
H19 -2.093197 6.504596 1.689082
H20 -0.704261 7.495431 2.151678
C31 0.774880 6.762542 -0.281515
H21 1.094800 7.818799 -0.455144
C32 0.749262 6.036938 -1.589770
H22 1.077114 6.720312 -2.403070
H23 -2.277704 5.172552 -2.964442
H24 -0.908040 5.923775 -3.813787
C33 0.572292 3.732386 -4.093872
H25 0.747167 4.598438 -4.775799
C34 0.379083 2.475452 -4.891359
H26 0.289921 2.725447 -5.974474
H27 -2.496424 0.594823 -4.452544
H28 -1.597836 0.766008 -5.967382
C35 0.238012 -0.996771 -5.523642
H29 -0.136236 -0.810959 -6.553639
C36 0.262285 -2.467723 -5.262535
H30 -0.171909 -3.004160 -6.139709
H31 -2.243409 -3.846755 -3.640348
H32 -1.056827 -4.589642 -4.723775
C37 0.858617 -5.090997 -2.661546
H33 0.955957 -6.070650 -3.182164
C38 1.167012 -5.269798 -1.201239
H34 1.475035 -6.325039 -1.007977
H35 -1.462435 -5.526440 0.946774
H36 0.078432 -6.407166 0.970080
C39 1.539845 -4.510430 2.207298
H37 1.874371 -5.560226 2.381168
C40 1.548723 -3.753022 3.505093
H38 1.887300 -4.427993 4.326425
H39 -1.369648 -2.482453 4.860137
H40 0.158785 -2.732336 5.718143
C41 -0.762908 -0.109340 3.959184
O -1.882074 -0.341219 3.503337
N 2.533986 -0.667398 4.637965
H41 1.178622 -0.650238 6.254631
C42 0.993210 1.060061 4.970371
H42 0.803878 1.548764 5.954914
